# Fluorinated Man_9_ as a High Mannose Mimetic to Unravel Its Recognition by DC-SIGN Using NMR

**DOI:** 10.1021/jacs.3c06204

**Published:** 2023-11-18

**Authors:** Adrián Silva-Díaz, Jonathan Ramírez-Cárdenas, Juan C. Muñoz-García, M. Carmen de la Fuente, Michel Thépaut, Franck Fieschi, Javier Ramos-Soriano, Jesús Angulo, Javier Rojo

**Affiliations:** † Instituto de Investigaciones Químicas (IIQ), CSIC − Universidad de Sevilla, Av. Américo Vespucio 49, Seville 41092, Spain; ‡ Univ. Grenoble Alpes, CNRS, CEA, IBS, Grenoble F-38044, France; § Institut Universitaire de France (IUF), Paris 75231, France

## Abstract

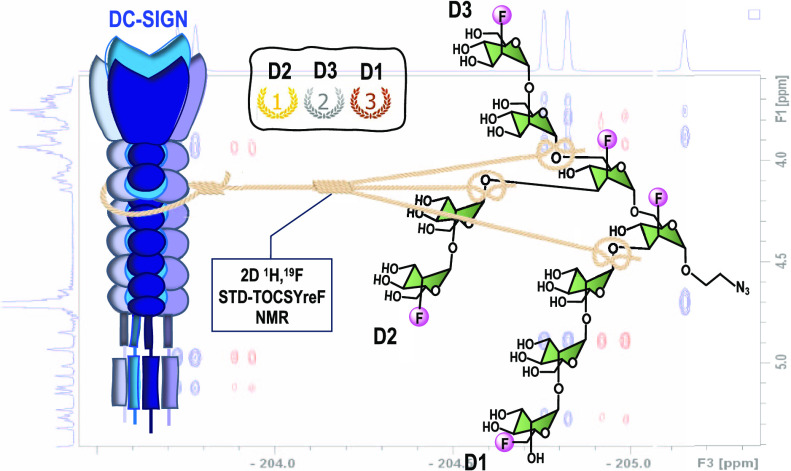

Lectins are capable of reading out the structural information contained in carbohydrates through specific recognition processes. Determining the binding epitope of the sugar is fundamental to understanding this recognition event. Nuclear magnetic resonance (NMR) is a powerful tool to obtain this structural information in solution; however, when the sugar involved is a complex oligosaccharide, such as high mannose, the signal overlap found in the NMR spectra precludes an accurate analysis of the interaction. The introduction of tags into these complex oligosaccharides could overcome these problems and facilitate NMR studies. Here, we show the preparation of the Man_9_ of high mannose with some fluorine tags and the study of the interaction with its receptor, dendritic cell-specific intercellular adhesion molecule-3-grabbing nonintegrin (DC-SIGN). This fluorinated ligand has allowed us to apply heteronuclear two-dimensional (2D) ^1^H,^19^F STD-TOCSYreF NMR experiments, using the initial slope approach, which has facilitated the analysis of the Man_9_/DC-SIGN interaction, unequivocally providing the binding epitope.

## Introduction

Dendritic cell-specific intercellular adhesion molecule-3-grabbing nonintegrin (DC-SIGN)^1^ is a C-type lectin that has attracted the interest of the scientific community during the last two decades due to its key role in pathogen infection and immunomodulation processes.^2^ This lectin recognizes carbohydrates, mainly high mannose (Man_9_GlcNAc_2_) and fucosylated oligosaccharides, in a Ca^2+^-dependent way. Man_9_GlcNAc_2_ decorates several pathogen envelope glycoproteins, such as HIV gp120 or Ebola virus GP1.^3,4^ Understanding how DC-SIGN recognizes Man_9_GlcNAc_2_ is a topic of enormous interest, but at the same time, it remains a big challenge. Although some X-ray crystal structures of complexes of DC-SIGN with oligosaccharides, from Manα1,2Man disaccharide up to Man_6_ hexasaccharide,^5−9^ have been solved, providing relevant structural features at the molecular level of this recognition, the structure of the DC-SIGN/Man_9_ complex has remained elusive despite all of the efforts devoted to its resolution. The size, flexibility, and nature of the Man_9_ oligosaccharide make this a challenging goal. Furthermore, the fixed picture obtained in the solid state for the multiple binding modes exhibited typically by carbohydrates/lectin recognition processes provides only partial information about the event.

NMR has been successfully used to analyze protein/carbohydrate complexes in solution at the molecular level.^10^ Different techniques such as STD NMR, tr-NOE, etc., in combination with CORCEMA-ST, can provide accurate information on the different binding modes that carbohydrates can show in the recognition process by lectins and facilitate the identification of the binding epitope.^11^ For complex oligosaccharides such as Man_9_ with strong NMR signal overlap, isotopic labeling (^15^N for the protein partner and/or ^13^C for the carbohydrate ligand) is required. Recently, the recognition of ^13^C-labeled Man_9_ by DC-SIGN and microvirin has been studied by NMR spectroscopy.^12^ These studies have shown that DC-SIGN recognizes an epitope of Man_9_ that does not agree with the information previously published^7^ based on the X-ray crystallographic analysis of its complex with Man_6_. Therefore, more information is necessary to have a better picture of how Man_9_ is recognized by DC-SIGN.

In this context, new strategies are demanded to develop useful chemical tools for gaining structural information on complex carbohydrate recognition by lectins in solution. Fluorinated mannosyl di- and trisaccharides have been synthesized and have proven to be valuable tools to diminish the signal overlap in ^1^H NMR and also to allow the use of ^19^F NMR.^13^ These fluorinated sugars have provided structural information about their interaction with DC-SIGN by NMR.^14^ For instance, using ^19^F NMR relaxation filter experiments allowed to evaluate the relative affinities and binding modes of several fluorinated monosaccharides (fucose, mannose, glucose, and galactose) toward DC-SIGN.^15^ Based on these precedents, we have synthesized a fluorine-tagged Man_9_ (F-Man_9_) with the aim of gaining fundamental information about the binding epitope of this Man_9_ oligosaccharide with DC-SIGN. The new picture of this recognition event will provide the basis for the design of better and more selective ligands to target this receptor with higher specificity.

## Results and Discussion

**Scheme 1:**
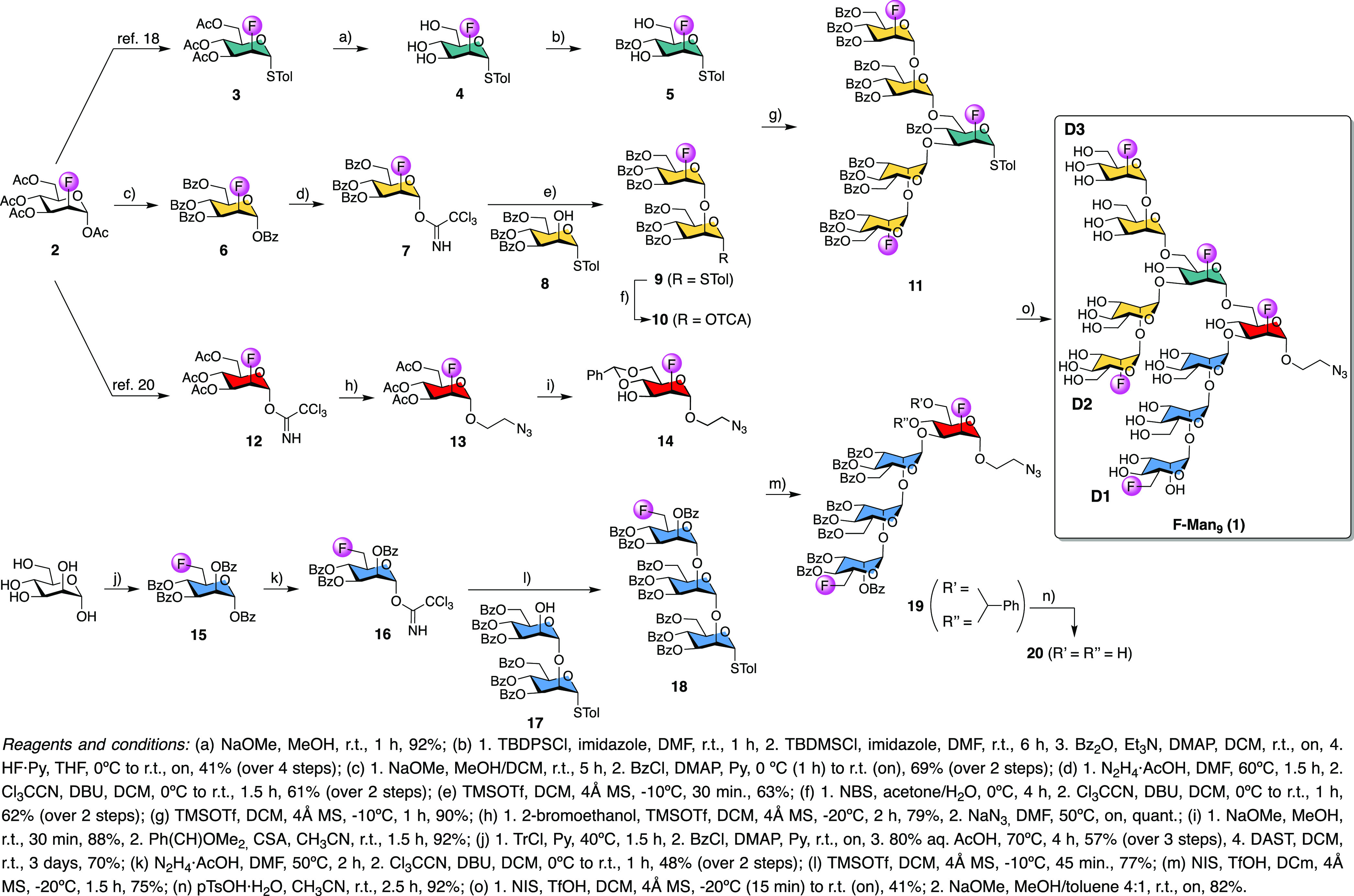
Synthesis of Fluorinated Man_9_ Oligosaccharide **1** (F-Man_9_)

To this end, and considering that the configuration of the reducing end plays only a minor role in DC-SIGN recognition,^16^ F-Man_9_**1** with α configuration at the reducing end, synthetically more accessible than the β-epimer, ^19^F-labeled at C-2 or C-6 positions, was rationally designed (Scheme 1). While the disaccharides (yellow) of the pentasaccharide building block were ^19^F-labeled at the C-2 positions for synthetic simplicity, the internal branching mannosyl unit (green) supporting the D2- and D3-arms was fluorinated at the same position because the C-6 position was glycosylated. Similarly, the reducing end terminal residue (red) was ^19^F-labeled at the C-2 position. C-6 fluorination in the trisaccharide (blue) was selected in order to avoid ^19^F-signal overlap, with the rest of the fluorinated mannosyl units complicating the NMR analysis.

Based on our previous convergent straightforward synthetic strategy to obtain Man_9_ oligosaccharides,^16,17^ we envisioned the synthesis of F-Man_9_ (**1**) in a similar way (Scheme 1). The synthesis of building blocks **5**, **7**, and **14** was afforded from 1,3,4,6-tetra-*O*-acetyl-2-deoxy-2-fluoro-d-mannopyranose **2**.^18^ Briefly, the synthesis of acceptor **5** started with the formation of glycosyl thiol **3**(18) followed by acetyl group removal to yield thioglycoside **4**. After that, four sequential synthetic steps with a final chromatography purification on silica gel afforded acceptor **5** in a 41% yield. This consecutive approach involved the chemoselective protection of C-6 and C-3 hydroxyl groups as silyl ethers, benzoylation at the C-4 position, and, finally, the selective removal of silyl groups. Second, a Zemplén deprotection–benzoylation sequence of **2** followed by the selective removal of the anomeric benzoyl ester and treatment with trichloroacetonitrile and DBU afforded the corresponding trichloroacetimidate **7**. The glycosylation reaction of this compound with acceptor **8**(19) in the presence of a catalytic amount of TMSOTf as a promoter afforded disaccharide **9** in a 76% yield. Disaccharide **9** was converted to the corresponding trichloroacetimidate **10** by the selective removal of the *S*-Tolyl group with NBS/water and subsequent reaction of the C-1 hydroxyl group with trichloroacetonitrile and DBU. Then, a 2 + 1 glycosylation of donor **10** with acceptor **5** yielded in a single step (90% yield) the pentasaccharide **11** using TMSOTf as the glycosylation promoter.

The synthesis of the terminal mannoside **14** started with the glycosylation of trichloroacetimidate **12**(20) with 2-bromoethanol. Next, the substitution of bromine with an azido group gave compound **13** in excellent yield. Zemplén deprotection and subsequent protection of C-4 and C-6 hydroxyl groups as benzylidene acetal provided the desired acceptor **14** in a very good yield.

The 6-deoxy-6-fluoro compound **15** was obtained from d-mannose via one-pot tritylation and benzoylation, followed by trityl removal under acidic conditions. Fluorination at C-6 with DAST was performed as previously described elsewhere to give compound **15**.^21^ This intermediate was subjected to anomeric deprotection with hydrazine acetate and treatment with trichloroacetonitrile in basic media to give trichloroacetimidate **16**, which was glycosylated with disaccharide acceptor **17**(17) to afford trisaccharide **18** in a 77% yield.

Glycosylation of the S-tolyl donor **18** with the hydroxyl group at C-3 of the acceptor **14** using NIS and TfOH at −20 °C gave tetrasaccharide **19** bearing the benzylidene group spanning C-4 and C-6 positions, which was removed in acidic media, with *p*-TsOH in acetonitrile affording tetrasaccharide **20** in high yield.

With the two required building blocks **11** and **20** in hand, the protected nonasaccharide **21** was assembled by glycosylation of the tetrasaccharide acceptor **20**, with pentasaccharide donor **11** in a 41% yield. The final step to obtain F-Man_9_**1** was the global deprotection of nonasaccharide **21** (82% yield) using NaOMe in methanol.

**Figure 1:**
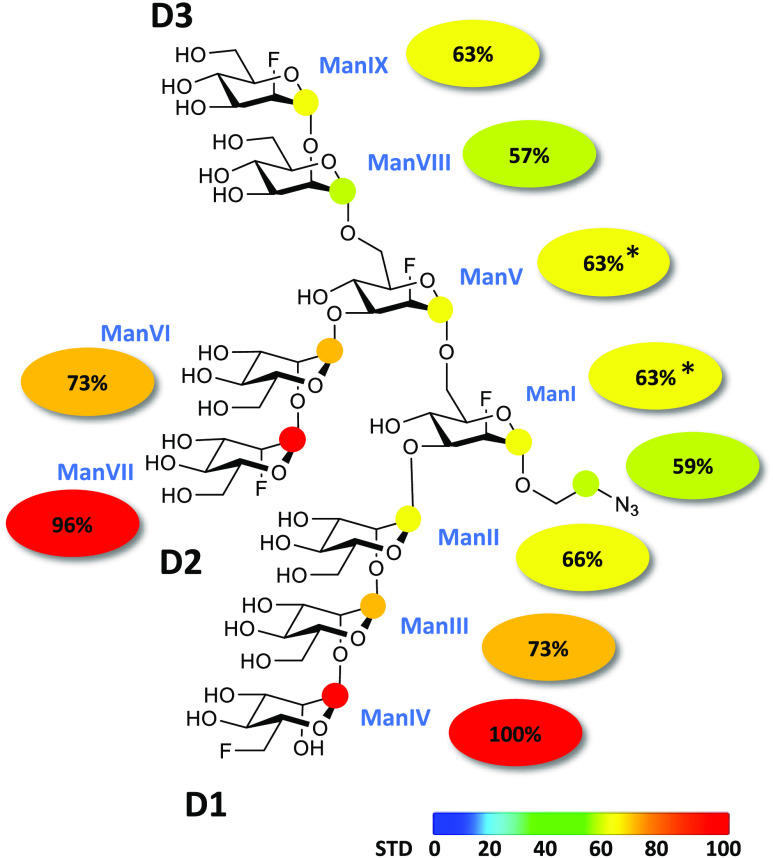
Binding epitope mapping of the F-Man_9_ oligosaccharide from 1D ^1^H STD NMR experiments. Selective protein ^1^H saturation was achieved by irradiation at 0.3 ppm at different saturation times. Colored spheres represent normalized STD NMR intensities, and the values are indicated by the colored ellipses. STD responses are shown only for protons that could accurately be measured (colored spheres). Asterisks indicate averaged STD values due to ^1^H overlap.

The interaction of F-Man_9_**1** with DC-SIGN was analyzed in solution using STD NMR experiments. First, standard one-dimensional (1D) ^1^H STD NMR experiments were carried out. Due to a strong ^1^H signal overlap, only STD signals from the anomeric protons could be accurately integrated (Figure 1). This provided very limited structural information about the oligosaccharide contacts with the protein (binding epitope mapping). This first analysis supported that the nonreducing Man residues at D1 and D2 branches make the most intimate contacts with DC-SIGN (Figure 1). However, to be able to answer the key question of whether there is a preferential recognition of any branch of the nonamannoside by DC-SIGN, a more detailed comparison of the total saturation transferred to each terminal mannose residue, measured as an average of different ring protons, was necessary, which was not possible by standard 1D ^1^H STD NMR.

We demonstrate here that the introduction of fluorine atoms into the F-Man_9_ oligosaccharide, indeed, is instrumental to access a more reliable description of the determinants for the molecular recognition by DC-SIGN. The presence of the spectroscopically orthogonal ^19^F spins allowed us to take advantage of the recently developed 2D ^1^H,^19^F STD-TOCSYreF NMR experiment.^13^

Thanks to the TOCSY mixing element, the information on saturation transfer is extended to further protons within the same spin system for each fluorinated Man residue of F-Man_9_. Besides, the dispersion of signals along the ^1^H and ^19^F frequency axes allowed an accurate integration of STD intensities beyond the limited set of well-dispersed ^1^H signals of the anomeric protons in the 1D ^1^H STD NMR approach (Figure S1). Any potential disadvantage in terms of an intrinsic loss of sensitivity for the heteronuclear transfer in this experiment is well compensated for by the much better spectral resolution, allowing more detailed epitope information to be obtained.^13^

**Figure 2:**
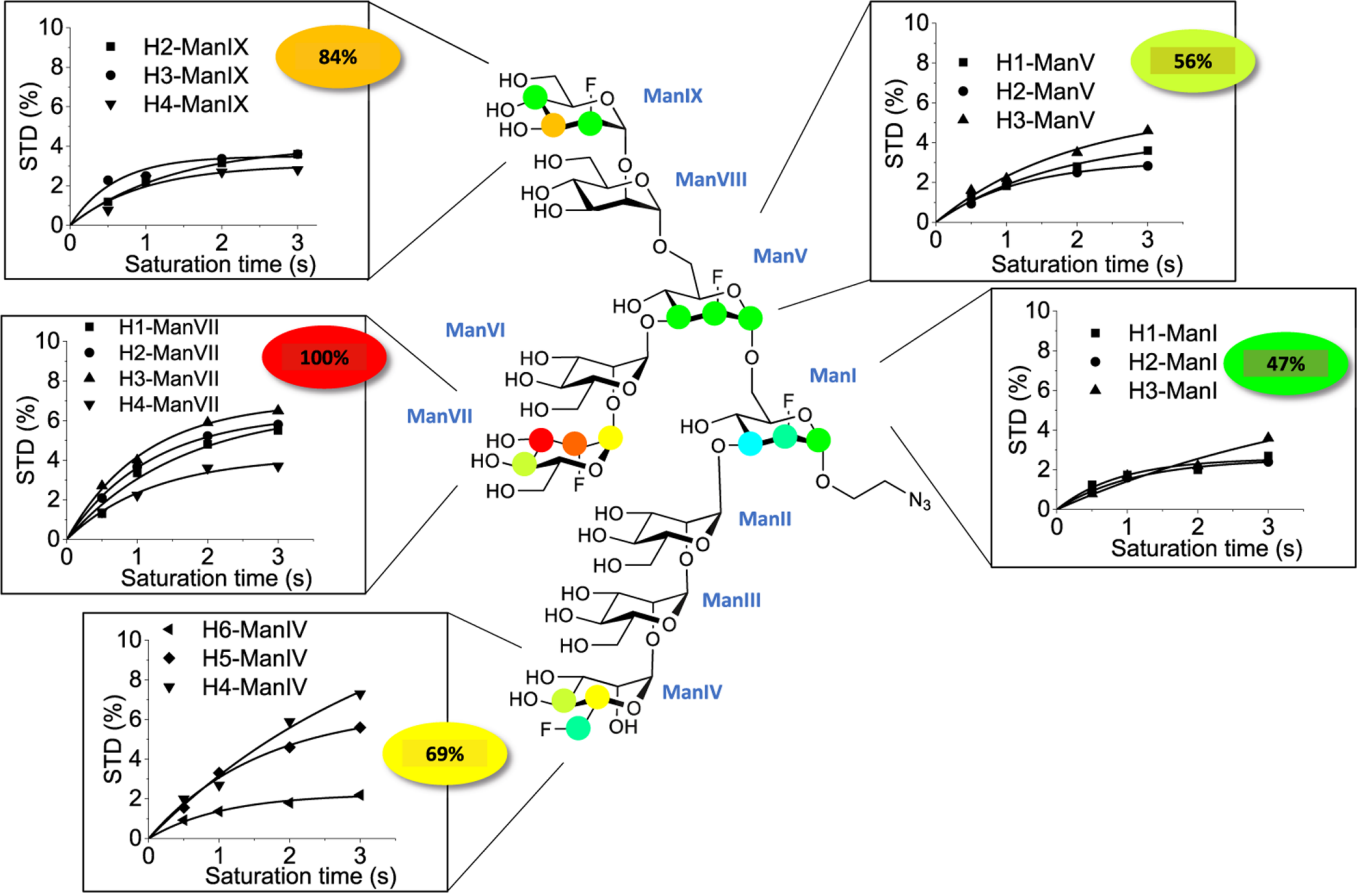
Structural details of the molecular recognition of the F-Man_9_ oligosaccharide by DC-SIGN from 2D ^1^H,^19^F STD-TOCSYreF NMR experiments. Selective protein saturation was achieved by irradiation at 0.3 ppm. Binding epitope mapping of F-Man_9_ from 2D ^1^H,^19^F STD-TOCSYreF NMR experiments, using an initial slope analysis of full STD build-up curves (shown as insets). A one-saturation time analysis was demonstrated to be inaccurate for determining the binding epitope of the oligosaccharide (Figure S4). Colored spheres represent normalized STD NMR intensities, only determined for protons that could accurately be measured. Colored ellipses show the total relative STD per mannose residue (average of initial slopes), using the same color code.

The analysis of the 2D ^1^H,^19^F STD-TOCSYreF NMR experiments led to a more complete and accurate binding epitope mapping of the oligosaccharide upon interaction with DC-SIGN (Figure 2). In fact, the results show a more detailed and significantly different picture of the epitope than in the previous 1D ^1^H STD NMR experiments (Figure 1). We demonstrate here, for the first time, that an initial slope analysis of full 2D ^1^H,^19^F STD-TOCSYreF build-up curves is necessary to obtain a reliable binding epitope mapping of the interaction to avoid T_1_ relaxation bias dominating measurements at one single saturation time (Table S1). Indeed, a simplified 2D ^1^H,^19^F STD-TOCSYreF single saturation time analysis led to an incorrect determination of the binding epitope mapping of the F-Man_9_ oligosaccharide for its binding to DC-SIGN (Figure S4).

Figure 2 shows the 2D ^1^H,^19^F STD-TOCSYreF NMR initial slope analysis, which demonstrates that DC-SIGN indeed preferentially recognizes the D2-arm in the context of the whole nonamannoside in solution. What is more, the recognition of the D3-arm is preferred over that of the D1-arm, in contrast to the results from the 1D ^1^H STD NMR analysis (Figure 1). The results show that both ramifications of the nonamannoside pending from the sugar ring ManV (D2- and D3-arms, from C-3 and C-6 positions of ManV, respectively) are better recognized by DC-SIGN compared to the D1-arm pending from ManI.

We further investigated whether the presence of the fluorine atoms in F-Man_9_ could induce conformational differences relative to natural Man_9_. An extensive conformational study of both ligands was carried out by NOESY and transferred-NOESY experiments.^22^ We analyzed 2D NOESY and tr-NOESY spectra of F-Man_9_ and Man_9_ in the absence and presence of DC-SIGN (Figure S5). No major changes in the conformations of the ligands were observed upon binding to the protein. Besides, for both ligands, an analysis of key strong NOEs from 2D tr-NOESY spectra concluded that there are no conformational differences between F-Man_9_ and the natural Man_9_ (Figure S6).

We also investigated if the presence of fluorine atoms could impact the affinity of the oligosaccharide for DC-SIGN. Competition studies between fluorinated and nonfluorinated Man_9_ were carried out using STD NMR experiments (Figure S7), clearly demonstrating that both F-Man_9_ and Man_9_ have the same affinity for DC-SIGN. All of these results support that fluorine atoms do not impact neither the conformation of the F-Man_9_ ligand nor the affinity of the interaction, validating the use of the fluorinated Man_9_ as a high mannose mimetic to unravel its recognition by DC-SIGN using NMR.

Finally, we used molecular dynamics (MD) simulations to correlate the experimental 2D ^1^H,^19^F STD-TOCSYreF NMR to three-dimensional (3D) molecular models of the complex of the nonamannoside with DC-SIGN. Starting from previous 3D models based on X-ray data,^7^ three molecular models of the F-Man_9_ and three of the natural Man_9_ oligosaccharides were built bound to DC-SIGN through each of the different arms (D1-, D2-, or D3-bound). The six starting protein–ligand 3D models for the D1, D2, and D3 Ca^2+^-coordinated orientations were built by superimposition of the nonreducing and adjacent mannose residues of each branch to the alike residues of the Man_6_ ligand bound to DC-SIGN CRD in the PDB structure 2IT5.^7^ It should be noted that this PDB structure shows two possible orientations for Man_6_, which are the so-called major and minor orientations. For ligand superimposition, we resorted to the experimental ^1^H STD NMR data (Figure 1), which clearly indicated that for the nonamannoside, only a bound conformation with a “minor” orientation of the nonreducing dimannoside on each arm is in agreement with the experimental binding epitope. In particular, ^1^H STD NMR data showed that the anomeric protons of both the nonreducing and adjacent residues point toward the protein surface, whereas they would be solvent-exposed in the “major” orientation. As a result, in the three starting structures, the residue adjacent to the nonreducing terminal of each of the nonamannoside branches (D1, D2, and D3, corresponding to ManIII, ManVI, and ManVIII, respectively) coordinated the Ca^2+^ ion of DC-SIGN CRD. No significant steric clashes were observed between the oligosaccharides and the protein in any of the orientations.

**Figure 3:**
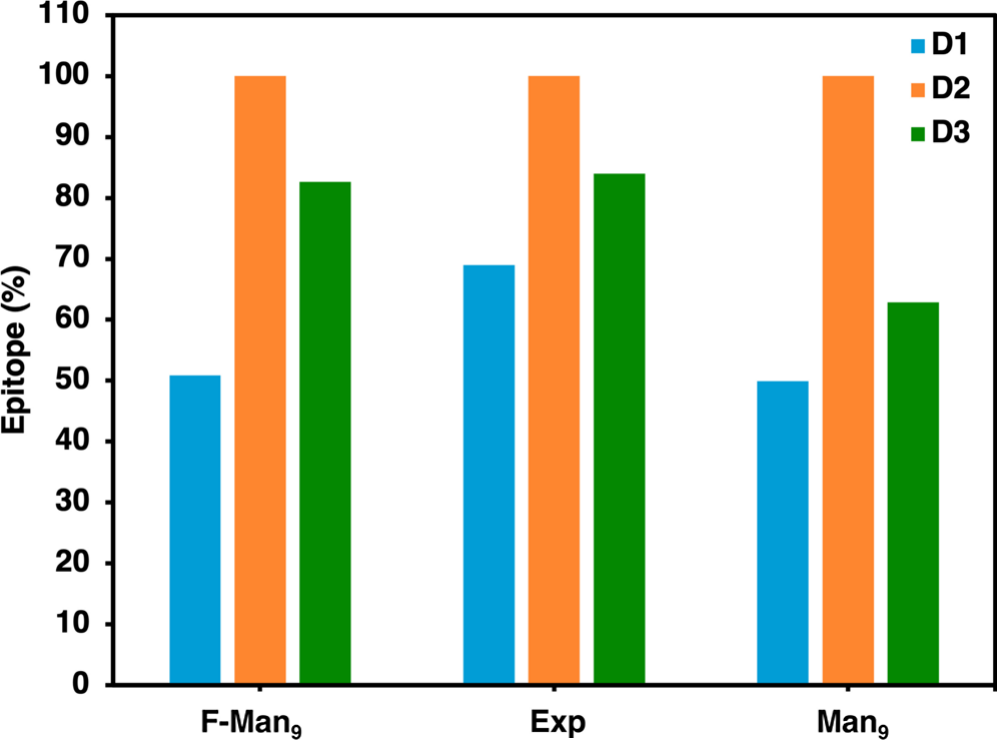
Bar graph comparing the experimental binding epitope mapping of F-Man_9_ (center) with the theoretical time-average contact-based epitopes derived from the MD simulations of F-Man_9_ (left) and Man_9_ (right). The epitope values are individually shown for all three branches: D1 (blue), D2 (orange), and D3 (green). Overall, the pattern D2 > D3 > D1, in terms of the preference of contacts with the protein, is observed in the MD simulations for both the natural and the fluorinated Man_9_, hence in agreement with the experimental epitope.

**Figure 4:**
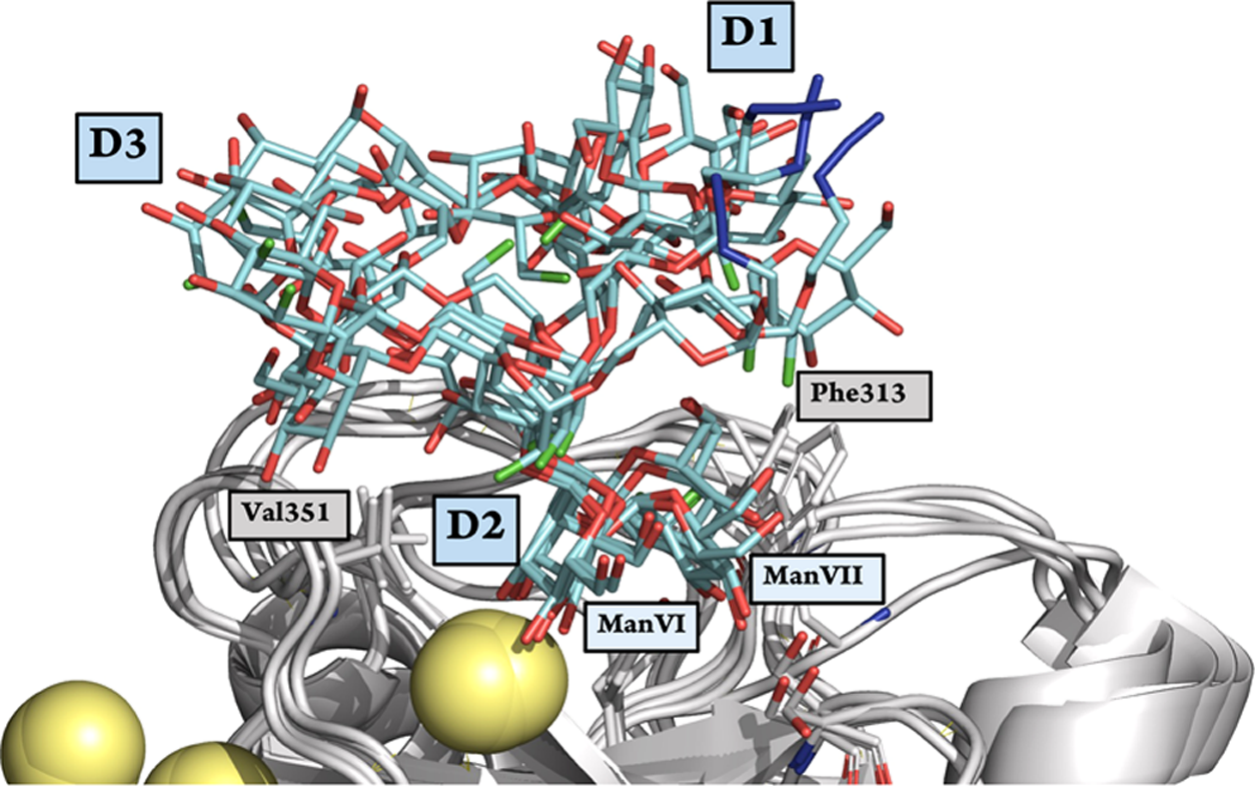
Superposition of four frames of the MD simulation of DC-SIGN CRD bound to F-Man_9_ with the D2 branch coordinating the Ca^2+^ ion. Protein and Ca^2+^ atoms are shown as light gray cartoon and pale-yellow spheres, respectively. The ligand is shown as cyan (carbon atoms), red (oxygen atoms), and green (fluorine atoms) sticks. Key protein residues involved in the interaction with the ligand are shown as light gray sticks and with labels next to them. The three F-Man_9_ branches are labeled as well as the two residues of the D2 branch occupying most of the binding pocket.

A normalized protein–ligand contact analysis along the MD simulations revealed that, globally, the nonamannoside oligosaccharide binds preferentially through the D2-arm, in excellent agreement with the experimental NMR data (Figure 3). The MD simulations showed a well-defined binding mode of the terminal ManVII–ManVI ending disaccharide within the binding pocket, with OH-3 and OH-4 hydroxyl groups of ManVI coordinating the Ca^2+^ ion, whereas the two remaining arms, D1 and D3, where highly flexible (Figure 4).

Overall, our data show that in the context of the whole nonamannoside, Man_9_, each branch is recognized by DC-SIGN CRD at the Ca^2+^ binding site through the residue adjacent to the nonreducing mannose, with a preference for the D2-arm. In the context of previous data in the literature, this mode of binding corresponds to the so-called “minor” orientation.^7,23^ Our experimental evidence, and theoretical data, are consistent with the existence of only one orientation (Figure 4) for each nonreducing dimannoside terminal in Man_9_, excluding the presence of the so-called “major” orientation.^7^ This highly relevant result indicates that, compared to the binding of smaller oligosaccharides mimicking different segments of Man_9_,^14,23^ the whole nonamannoside does not show multiple binding modes for the recognition of its nonreducing terminal mannose residues by DC-SIGN.

This is the first time that such a detailed study of the preferential branch recognition of Man_9_ by DC-SIGN has been performed in solution, and the results further underscore the importance of using ^19^F labeling, along with an initial slope analysis of the heteronuclear ^19^F-edited STD NMR experiments for such branched large oligosaccharides to elucidate the populations of each branch in the protein-bound state.

## Supporting Information Available

Experimental procedures, characterization data (^1^H, ^13^C, and ^19^F NMR and HRMS), copies of NMR spectra of new compounds, and NMR and computational details (PDF)
