# Supplementary material for: Fluorinated Man9 as a High Mannose Mimetic to Unravel Its Recognition by DC-SIGN Using NMR
Source: J Am Chem Soc. 2023 Nov 18;145(48):26009–15. doi: 10.1021/jacs.3c06204 (PMC10852354; doi:10.1021/jacs.3c06204)
Supplement: Supplementary file 1 — ja3c06204_si_001.pdf [file ja3c06204_si_001.pdf]

Fluorinated Man$_9$ as a high mannose mimetic to unravel its recognition by DC-SIGN using NMR.

Adrián Silva-Díaz,[a,‡] Jonathan Ramírez-Cárdenas,[a,‡] Juan C. Muñoz-García,[a] M. Carmen de la Fuente,[a] Michel Thépaut,[b] Franck Fieschi,[b,c] Javier Ramos-Soriano,*[,a] Jesús Angulo,*[,a] Javier Rojo.*[,a]

[a] Instituto de Investigaciones Químicas (IIQ), CSIC – Universidad de Sevilla, Av. Américo Vespucio 49, Seville 41092, Spain; [b] Univ. Grenoble Alpes, CNRS, CEA, IBS, Grenoble, F-38044, France; [c] Institut Universitaire de France (IUF), Paris, 75231, France.

[‡]These authors contributed equally to this work.

*Corresponding authors: fj.ramos@iiq.csic.es; j.angulo@iiq.csic.es; javier.rojo@iiq.csic.es

1. General methods……………………………………………………………………..S2
2. Synthesis and characterization……………………………………………………............... S3
3. ^{1}H, ^{13}C and ^{19}F NMR spectra…………………………………………………........... S19
4. DC-SIGN sample preparation…………………………………………………………S39
5. NMR experiments with DC-SIGN………………………………………………..........S39
6. Molecular dynamics…………………………………………………………………….S46
7. References…………………………………………………………………………........... S48

1. General methods.

Reagents and solvents were purchased as reagent grade and used without further purification. When anhydrous DMF, pyridine, acetonitrile and methanol were used, commercial grade solvents were dried in the presence of activated 4Å molecular sieves (3Å in the case of methanol). Anhydrous DCM was obtained from distillation of commercial DCM over CaH_2. When dry solvents were employed, reactions were performed under Ar or N_2 atmosphere. MiliQ H_2O was purified with a Milli-Q purification system from Millipore (18.3 Ω). 1,3,4,6-Tetra-*O*-acetyl-2-deoxy-2-fluoro-D-mannopyranose (**2**),[1] *p*-methylphenyl 3,4,6-tri-*O*-acetyl-2-deoxy-2-fluoro-1-thio-α-D-mannopyranoside (**3**),[1] *p*-methylphenyl 3,4,6-tri-*O*-benzoyl-1-thio-α-D-mannopyranoside (**8**),[2] 3,4,6-tri-*O*-acetyl-2-fuoro-2-deoxy-α-D-manno-pyranosyl trichloroacetimidate (**12**),[3] and *p*-methylphenyl 3,4,6-tri-*O*-benzoyl-α-D-mannopyranosyl-(1→2)-3,4,6-tri-*O*-benzoyl-1-thio-α-D-mannopyranoside (**17**)[2] were prepared according to previously reported procedures. Thin layer chromatography (TLC) analyses were performed on silica gel 60 F_{254} precoated on aluminium plates (Merck), with detection by UV light (λ = 254 nm) and charred with Mostain or anisaldehyde as development reagents. Column chromatography was carried out on silica gel 60 (0.040–0.063 mm or 0.015–0.04 mm; Merck) or by Sephadex LH20 or G25 from GE Healthcare (Barcelona, Spain) gel filtration. ^1H, ^{19}F and ^{13}C NMR spectra were obtained for solutions in CD_3OD, $CDCl_3$, or D_2O at 298K on Bruker DRX400, Bruker DRX-500 and Bruker 600 MHz NMR spectrometers with solvent peaks as reference. All chemical shifts were reported in ppm (δ) and coupling constants (J) are reported in Hertz (Hz). All the assignments were confirmed by one- and two-dimensional NMR experiments (COSY, HSQC and HMBC). Electrospray mass spectra (ESI MS) were registered at the Mass Spectrometry Service of the Institute for Chemical Research (IIQ, CSIC-US) using an Elute UHPLC system coupled to a Bruker Amazon SL spectrometer instrument. High resolution mass spectra (HR MS) were carried out at Mass Spectrometry Service of CITIUS (University of Seville) using a Thermo Scientific Orbitrap Elite.

ABBREVIATIONS. ACN: acetonitrile, Bz_2O: benzoic anhydride, BzCl: benzoyl chloride, CSA: camphorsulfonic acid, DAST: (diethylamino)sulfur trifluoride, DBU: 1,8-diazabicyclo[5.4.0]undec-7-ene, DCM: dichloromethane, DC-SIGN: Dendritic Cell Specific Intercellular adhesion molecule 3 Grabbing Non-integrin, DMAP: 4-(dimethylamino)pyridine, DMF: dimethylformamide, ECD: extra-cellular domain, MS: molecular sieves, NBS: *N*-bromosuccinimide, NIS: *N*-iodosuccinimide, NOE: Nuclear Overhauser Effect, NOESY: Nuclear Overhauser Enhacement SpectroscopY, on: overnight, PTSA·H_2O: *p*-toluenesulfonic acid monohydrate, Py: pyridine, r.t.: room temperature, STD: Saturation Transfer Difference, STD-TOCSYreF, Saturation Transfer Difference with relay to ^{19}F and TOCSY transfer, TBDMSCl: *tert*-butyldimethylsilyl chloride, TDBPSCl: *tert*-butyl(chloro)diphenylsilane chloride, TfOH: trilic acid, THF: tetrahydrofuran, TMSOTf: trimethysilyl triflate, TOCSY: Total Correlation Spectroscopy, TrCl: trytil chloride, tr-NOESY: transferred NOESY.

2. Synthesis and characterization.

p-Methylphenyl 2-deoxy-2-fluoro-1-thio-α-D-mannopyranoside (4)

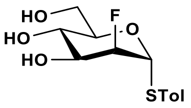

To a solution of compound **3** (704 mg, 1.70 mmol) in MeOH (13 mL), NaOMe (62 mg, 1.02 mmol) was added. After 1 h of stirring at r.t., the reaction mixture was neutralized with Amberlite IR-120 (H^+) resin, filtered and concentrated to give **4** (450 mg, 92%) as a white amorphous solid. ^{1}H–NMR (400 MHz, CD_3OD) *δ:* 7.43 (d, *J* = 8.3, 2H, $H_{o\text{-Tol}}$), 7.16 (d, *J* = 8.3, 2H, $H_{m\text{-Tol}}$), 5.52 (ddd, $^3J_{H1\text{-}F}$ = 14.5, $^3J_{H1\text{-}H2}$ = 1.8, *J* = 0.5, 1H, H-1), 4.88 (ddd, $^2J_{H2\text{-}F}$ = 50.2, $^3J_{H2\text{-}H3}$ = 2.7, $^3J_{H2\text{-}H1}$ = 1.8, 1H, H-2), 4.05 (m, 1H, H-5), 3.84 (dd, $^2J_{H6a\text{-}H6b}$ = 11.9, $^3J_{H6a\text{-}H5}$ = 2.5, H-6a), 3.79 – 3.68 (m, 3H, H-3, H-4, H-6b), 2.32 (s, 3H, CH_3); ^{13}C–NMR (101 MHz, CD_3OD) *δ:* 139.4 ($C_{p\text{-Tol}}$), 133.8 ($C_{o\text{-Tol}}$), 131.2 ($C_{ipso\text{-Tol}}$), 130.9 ($C_{m\text{-Tol}}$), 92.7 (d, $^1J_{C2\text{-}F}$ = 184.2, C-2), 88.1 (d, $^2J_{C1\text{-}F}$ = 22.7, C-1), 75.8 (C-5), 72.3 (d, $^2J_{C3\text{-}F}$ = 18.5, C-3), 68.7 (C-4), 62.4 (C-6), 21.1 (CH_3); ^{19}F–NMR (376 MHz, CD_3OD) *δ:* -191.4; ESI–MS *m/z* calcd. for $C_{13}H_{17}N_3SO_4F$: 288.9; found: 311.1 $[M+Na]^+$; ESI–HRMS *m/z* calcd. for $C_{13}H_{17}N_3SO_4FNa$ $[M+Na]^+$: 311.0724; found: 311.0721.

p-Methylphenyl 4-*O*-benzoyl-2-deoxy-2-fluoro-1-thio-α-D-mannopyranoside (5)

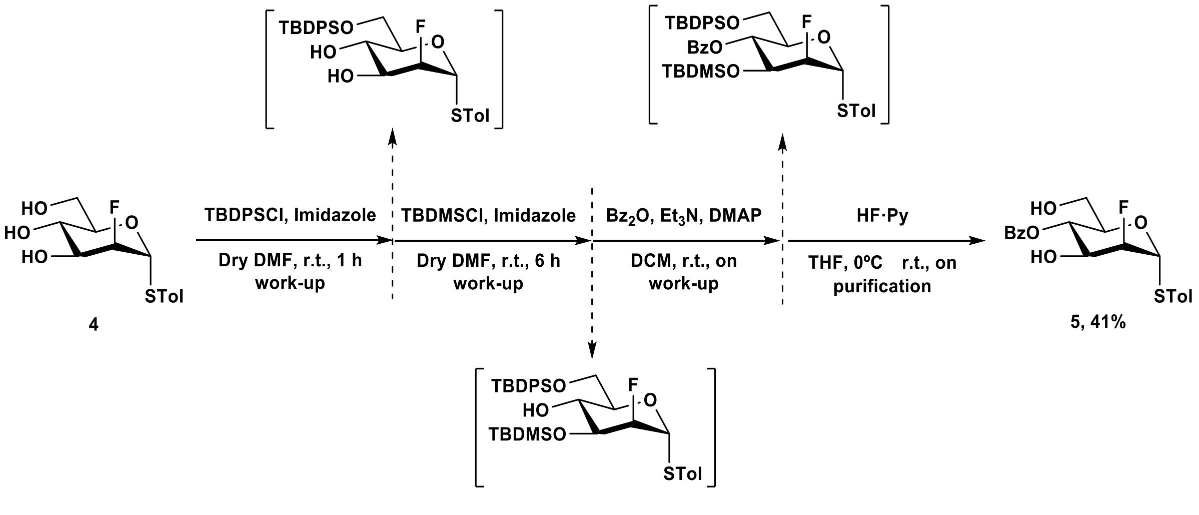

To a solution of compound **4** (95 mg, 0.33 mmol) and imidazole (44 mg, 0.64 mmol) in dry DMF (1.3 mL), TBDPSCl (114 µL, 0.43 mmol) was added. After 1 h of stirring at r.t., the solution was diluted with DCM (15 mL) and washed with brine (3 x 10 mL). The aqueous phase was extracted with DCM (10 mL), and both organic phases were dried over anh. $MgSO_4$, filtered and concentrated. The resulting

crude containing 6-TBDPS derivative and imidazole (42 mg, 0.65 mmol) were dissolved in dry DMF (2 mL), and TBDMSCl (112 mg, 0.72 mmol) was added. The reaction mixture was stirred at r.t. for 6 h. The mixture was diluted with DCM (15 mL) and washed with brine (3 x 10 mL). The organic phase was dried over anh. $MgSO_4$, filtered and concentrated. To a stirred solution of the crude containing 4,6-protected derivative in DCM (5 mL), Bz_2O (224 mg, 0.99 mmol), Et_3N (150 µL, 1.08 mmol) and DMAP (cat.) were subsequently added and the reaction mixture was stirred overnight at r.t. The reaction mixture was washed with 1 M HCl (10 mL), sat. $NaHCO_3$ aq. soln. (10 mL) and brine (10 mL). The organic phase was dried over anh. $MgSO_4$, filtered and concentrated under vacuum. To the resulting mixture containing fully protected mannopyranoside in THF (1 mL) was slowly added a cold solution of HF·Py complex (0.2 mL, 7.25 mmol). After that, the reaction mixture was stirred overnight at r.t. The reaction mixture was diluted with DCM (15 mL) and washed successively with sat. $NaHCO_3$ aq. soln. (3 x 10 mL) and H_2O (10 mL). The organic phase was dried over anh. $MgSO_4$, filtered and concentrated. The crude was purified by silica gel column chromatography (*n*-hexane/EtOAc 4:1 → 3:1), affording the key intermediate **5** (102 mg, 41%) as a yellowish foam. ^1H–NMR (400 MHz, $CDCl_3$) *δ:* 8.09 (d, *J* = 8.1, 2H, $H_{o\text{-Ph}}$), 7.62 (m, 1H, $H_{p\text{-Ph}}$), 7.49 (m, 2H, $H_{m\text{-Ph}}$), 7.40 (d, *J* = 8.3, 2H, $H_{o\text{-Tol}}$), 7.16 (d, *J* = 8.3, 2H, $H_{m\text{-Tol}}$), 5.65 (m, 1H, H-1), 5.40 (td, $^3J_{H4\text{-}H3} = {}^3J_{H4\text{-}H5} = 9.9$, *J* = 0.7, 1H, H-4), 5.06 (ddd, $^2J_{H2\text{-}F} = 49.5$, $^3J_{H2\text{-}H3} = 2.7$, $^3J_{H2\text{-}H1} = 1.7$, 1H, H-2), 4.44 – 4.39 (m, 1H, H-5), 4.15 (ddd, $^3J_{H3\text{-}F} = 28.5$, $^3J_{H3\text{-}H4} = 9.7$, $^3J_{H3\text{-}H2} = 2.6$, 1H, H-3), 3.77 (dd, $^3J_{H6a\text{-}H6b} = 12.8$, $^3J_{H6a\text{-}H5} = 2.3$, 1H, H-6a), 3.71 (dd, $^3J_{H6b\text{-}H6a} = 12.6$, $^3J_{H6b\text{-}H5} = 4.5$, 1H, H-6b), 2.58 (br s, 1H, OH-3), 2.35 (s, 3H, CH_3), 2.15 (br s, 1H, OH-6); ^{13}C–NMR (101 MHz, $CDCl_3$) *δ:* 167.2 (CO), 138.9 ($C_{p\text{-Tol}}$), 133.9 ($C_{p\text{-Ph}}$), 132.9 ($C_{o\text{-Tol}}$), 130.3 ($C_{m\text{-Tol}}$), 130.1 ($C_{o\text{-Ph}}$), 129.1 ($C_{ipso\text{-Ph}}$), 128.7 ($C_{ipso\text{-Tol}}$), 128.7 ($C_{m\text{-Ph}}$), 91.7 (d, $^1J_{C2\text{-}F} = 184.6$, C-2), 86.3 (d, $^2J_{C1\text{-}F} = 23.9$, C-1), 71.8 (C-5), 70.2 (C-4), 70.1 (d, $^2J_{C3\text{-}F} = 17.9$, C-3), 61.5 (C-6), 21.3 (CH_3); ^{19}F–NMR (376 MHz, $CDCl_3$) *δ:* -191.7; ESI–MS *m/z* calcd. for $C_{20}H_{21}SO_5F$: 392.1; found: 415.1 $[M+Na]^+$; ESI–HRMS *m/z* calcd. for $C_{20}H_{21}SO_5FNa$ $[M+Na]^+$: 415.0985; found: 415.0985.

1,3,4,6-Tetra-*O*-benzoyl-2-deoxy-2-fluoro-α-D-mannopyranose (6)

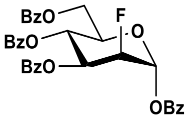

To a solution of 1,3,4,6-tetra-*O*-acetyl-2-deoxy-2-fluoro-D-mannopyranose (**2**) (3.64 g, 10.38 mmol) in a mixture of DCM/MeOH (4:1, 125 mL), 1 M methanolic NaOMe solution was added until reaching pH = 9 (~2 mL). The reaction mixture was stirred at r.t. for 5 h, then neutralized with Amberlite IR-120 (H^+) resin, filtered and concentrated. To a solution of the resulting crude containing fully deprotected derivative in dry Py (22 mL) cooled to 0ºC, BzCl (6.8 mL, 58.00 mmol) and DMAP (cat.) were added. The reaction mixture was allowed to warm to r.t., and stirred overnight. After removal of the solvent,

the residue was diluted with DCM (200 mL) and washed with H_2O (50 mL), sat. $NaHCO_3$ aq. soln. (2 x 100 mL) and brine (2 x 100 mL). The organic phase was dried over anh. $MgSO_4$, filtered and concentrated. The resulting crude was purified by silica gel column chromatography (toluene/acetone 80:1), to give compound **6** (4.30 g, 69%) as a white foam. ^{1}H–NMR (400 MHz, $CDCl_3$) *δ:* 8.17 – 8.13 (m, 2H, H_{Ar}), 8.03 – 7.98 (m, 4H, H_{Ar}), 7.97 – 7.93 (m, 2H, H_{Ar}), 7.70 – 7.65 (m, 1H, H_{Ar}), 7.58 – 7.48 (m, 5H, H_{Ar}), 7.43 – 7.33 (m, 6H, H_{Ar}), 6.66 (dd, $^3J_{H1-F}$ = 6.6, $^3J_{H1-H2}$ = 1.9, 1H, H-1), 6.11 (t, $^3J_{H4-H3}$ = $^3J_{H4-H5}$ =10.2, 1H, H-4), 5.84 (ddd, $^3J_{H3-F}$ = 27.8, $^3J_{H3-H4}$ = 10.6, $^3J_{H3-H2}$ = 2.5, 1H, H-3), 5.24 – 5.10 (m, 1H, H-2), 4.62 (dd, $^2J_{H6a-H6b}$ = 11.7, $^3J_{H6a-H5}$ = 2.5, 1H, H-6a), 4.55 – 4.45 (m, 2H, H-5, H-6b); ^{13}C–NMR (101 MHz, $CDCl_3$) *δ:* 166.3 (CO), 166.0 (CO), 165.3 (CO), 163.9 (CO), 134.3 (C_{Ar}), 133.8 (C_{Ar}), 133.7 (C_{Ar}), 133.2 (C_{Ar}), 130.2 (C_{Ar}), 130.1 (C_{Ar}), 130.0 (C_{Ar}), 129.9 (C_{Ar}), 129.7 (C_{Ar}), 129.0 (C_{Ar}), 128.9 (C_{Ar}), 128.8 (C_{Ar}), 128.7 (C_{Ar}), 128.6 (C_{Ar}), 128.5 (C_{Ar}), 91.1 (d, $^2J_{C1-F}$ = 30.8, C-1), 86.5 (d, $^1J_{C2-F}$ = 182.4, C-2), 71.4 (C-5), 70.7 (d, $^2J_{C3-F}$ = 17.5, C-3), 66.1 (C-4), 62.8 (C-6); ^{19}F–NMR (376 MHz, $CDCl_3$) *δ:* -203.9; ESI–MS *m/z* calcd. for $C_{34}H_{27}O_9F$: 598.2; found: 616.3 $[M+NH_4]^+$; ESI–HRMS *m/z* calcd. for $C_{34}H_{21}O_9FNa$ $[M+Na]^+$: 621.1531; found: 621.1531.

3,4,6-Tri-*O*-benzoyl-2-deoxy-2-fluoro-α-D-mannopyranosyl trichloroacetimidate (7)

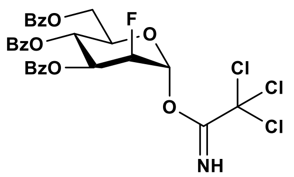

Monosaccharide **6** (2.01 g, 3.35 mmol) was dissolved in dry DMF (30 mL) and N_2H_4·AcOH (481 mg, 5.07 mmol) was added. The reaction mixture was stirred at 60ºC for 1.5 h. After removal of the solvent, the crude was diluted with DCM (100 mL) and washed with brine (100 mL). The organic phase was dried over anh. $MgSO_4$, filtered and concentrated. The residue was purified by silica gel column chromatography (*n*-hexane/EtOAc 3:1→2:1), yielding the corresponding hemiacetal (1.38 g, 83%) as a white foam. To a solution of the resulting hemiacetal in dry DCM (53 mL) cooled to 0ºC, trichloroacetonitrile (1.7 mL, 16.62 mmol) and DBU (0.2 mL, 1.34 mmol) were added. The reaction mixture was stirred at r.t. for 1.5 h. After removal of the solvent, the crude was purified by silica gel column chromatography (*n*-hexane/EtOAc 4:1) to afford the trichloroacetimidate **7** (1.31 g, 74%) as a yellowish foam. ^{1}H–NMR (400 MHz, $CDCl_3$) *δ:* 8.86 (s, 1H, NH), 8.04 – 7.94 (m, 6H, H_{Ar}), 7.57 – 7.49 (m, 3H, H_{Ar}), 7.44 – 7.34 (m, 6H, H_{Ar}), 6.60 (dd, $^3J_{H1-F}$ = 6.2, $^3J_{H1-H2}$ = 2.0, 1H, H-1), 6.06 (t, $^3J_{H4-H3}$ = $^3J_{H4-H5}$ = 10.2, 1H, H-4), 5.75 (ddd, $^3J_{H3-F}$ = 27.6, $^3J_{H3-H4}$ = 10.4, $^3J_{H3-H2}$ = 2.6, 1H, H-3), 5.29 – 5.14 (m, 1H, H-2), 4.64 (dd, $^2J_{H6a-H6b}$ = 11.9, $^3J_{H6a-H5}$ = 2.7, 1H, H-6a), 4.57 (m, 1H, H-5), 4.48 (dd, $^2J_{H6b-H6a}$ = 12.1, $^3J_{H6b-H5}$ = 4.9, 1H, H-6b); ^{13}C–NMR (101 MHz, $CDCl_3$) *δ:* 166.2 (CO), 165.9 (CO), 165.3 (CO), 160.0 (CN), 133.8 (C_{Ar}), 133.7 (C_{Ar}), 133.3 (C_{Ar}), 130.1 (C_{Ar}), 130.0 (C_{Ar}), 129.9 (C_{Ar}), 129.7 (C_{Ar}), 128.8 (C_{Ar}),

128.7 (C_{Ar}), 128.6 (C_{Ar}), 128.5 (C_{Ar}), 94.3 (d, $^{2}J_{C1-F}$ = 31.2, C-1), 90.6 (CCl_3), 85.8 (d, $^{1}J_{C2-F}$ = 182.0, C-2), 71.7 (C-5), 70.6 (d, $^{2}J_{C3-F}$ = 17.0, C-3), 65.9 (C-4), 62.8 (C-6); ^{19}F–NMR (376 MHz, $CDCl_3$) *δ:* -204.9; ESI–MS *m/z* calcd. for $C_{29}H_{23}NCl_3O_8F$: 637.0; found: 662.1 $[M+Na]^+$; ESI–HRMS *m/z* calcd. for $C_{29}H_{23}NCl_3O_8FNa$ $[M+Na]^+$: 660.0366; found: 660.0367.

***p*-Methylphenyl 3,4,6-tri-*O*-benzoyl-2-deoxy-2-fluoro-α-D-mannopyranosyl-(1→2)-3,4,6-tri-*O*-benzoyl-1-thio-α-D-mannopyranoside (9)**

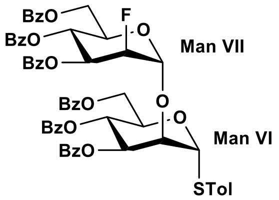

A mixture containing glycosyl donor **7** (75 mg, 0.13 mmol), monosaccharide **8** (108 mg, 0.17 mmol) and 4Å MS (powder, 0.5 g) in dry DCM (5 mL) was stirred at -10ºC for 15 min. Then, TMSOTf (7 µL, 0.04 mmol) was added and the resulting mixture was stirred at -10ºC for 30 min. The reaction mixture was quenched with Et_3N (0.1 mL), filtered through Celite and washed with DCM. After removal of the solvent, the crude was purified by silica gel column chromatography (*n*-hexane/EtOAc 4:1) to give the disaccharide **9** (85 mg, 63%) as a white foam. ^{1}H–NMR (400 MHz, $CDCl_3$) *δ:* 8.05 – 7.96 (m, 10H, H_{Ar}), 7.92 (d, *J* = 8.4, 2H, H_{Ar}), 7.56 – 7.44 (m, 6H, H_{Ar}), 7.43 – 7.29 (m, 14H, H_{Ar}), 6.96 (d, *J* = 8.0, 2H, $H_{m\text{-}Tol}$), 5.97 (t, $^{3}J_{H4-H3}$ = $^{3}J_{H4-H5}$ = 9.9, 1H, H-4_{VI}), 5.93 (t, $^{3}J_{H4-H3}$ = $^{3}J_{H4-H5}$ = 10.0, 1H, H-4_{VII}), 5.85 – 5.72 (m, 3H, H-1_{VI}, H-3_{VI}, H-3_{VII}), 5.30 (dd, $^{3}J_{H1-F}$ = 7.4, $^{3}J_{H1-H2}$ = 1.8, 1H, H-1_{VII}), 5.21 – 5.06 (m, 1H, H-2_{VII}), 5.00 – 4.94 (m, 1H, H-5_{VI}), 4.70 (dd, $^{3}J_{H2-H3}$ = 3.3, $^{3}J_{H2-H1}$ = 1.9, 1H, H-2_{VI}), 4.64 – 4.47 (m, 4H, H-6_{VI}, H-5_{VII}, H-$6a_{VII}$), 4.41 (dd, $^{2}J_{H6b-H6a}$ = 12.2, $^{3}J_{H6b-H5}$ = 5.3, 1H, H-$6b_{VII}$), 2.26 (s, 3H, CH_3); ^{13}C–NMR (101 MHz, $CDCl_3$) *δ:* 166.3 (CO), 166.3 (CO), 165.6 (CO), 165.5 (2 CO), 165.4 (CO), 138.5 ($C_{p\text{-}Tol}$), 133.8 (C_{Ar}), 133.6 (C_{Ar}), 133.5 (C_{Ar}), 133.2 (C_{Ar}), 132.7 (C_{Ar}), 130.2 ($C_{m\text{-}Tol}$), 130.0 (C_{Ar}), 129.9 (C_{Ar}), 129.8 (C_{Ar}), 129.6 (C_{Ar}), 129.1 (C_{Ar}), 128.9 (C_{Ar}), 128.8 (C_{Ar}), 128.7 (C_{Ar}), 128.6 (C_{Ar}), 128.5 (C_{Ar}), 128.5 (C_{Ar}), 99.1 (d, $^{2}J_{C1-F}$ = 30.3, C-1_{VII}), 87.1 (C-1_{VI}), 86.9 (d, $^{1}J_{C2-F}$ = 181.1, C-2_{VII}), 77.8 (C-2_{VI}), 71.6 (C-3_{VI}), 70.4 (d, $^{2}J_{C3-F}$ = 17.0, C-3_{VII}), 70.0 (C-5_{VII}), 69.9 (C-5_{VI}), 67.6 (C-4_{VI}), 66.5 (C-4_{VII}), 63.7 (C-6_{VI}), 63.1 (C-6_{VII}), 21.2 (CH_3); ^{19}F–NMR (376 MHz, $CDCl_3$) *δ:* -203.4; ESI–MS *m/z* calcd. for $C_{61}H_{51}SO_{15}F$: 1074.3; found: 1092.5 $[M+NH_4]^+$; ESI–HRMS *m/z* calcd. for $C_{61}H_{51}SO_{15}FNa$ $[M+Na]^+$: 1097.2825; found: 1097.2818.

3,4,6-Tri-*O*-benzoyl-2-deoxy-2-fluoro-α-D-mannopyranosyl-(1→2)-3,4,6-tri-*O*-benzoyl-α-D-mannopyranosyl trichloroacetimidate (10)

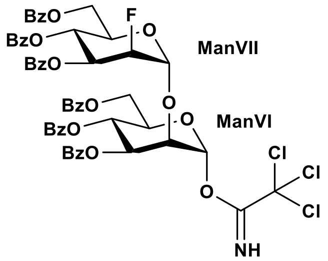

To a solution of disaccharide **9** (553 mg, 0.51 mmol) in a mixture of acetone/H_2O (9:1, 10 mL) cooled to 0ºC, NBS (277 mg, 1.54 mmol) was added. After 4 h of stirring at 0ºC, the mixture was diluted with EtOAc (30 mL) and washed with sat. $NaHCO_3$ aq. soln. (15 mL). The organic phase was dried over anh. $MgSO_4$, filtered, and the solvent was evaporated. The crude was purified by silica gel column chromatography (*n*-hexane/EtOAc 2:1), giving the corresponding hemiacetal (379 mg, 76%) as a white foam. The hemiacetal was dissolved in dry DCM (8 mL) and the solution was cooled at 0ºC. Trichloroacetonitrile (0.21 mL, 2.06 mmol) and DBU (31 µL, 0.21 mmol) were added, and the reaction mixture was stirred at r.t. for 1 h. After solvent removal, the crude was purified by silica gel column chromatography (*n*-hexane/EtOAc 1:1) to yield the trichloroacetimidate **10** (355 mg, 82%) as a white foam. ^{1}H–NMR (400 MHz, $CDCl_3$) *δ:* 8.69 (s, 1H, NH), 8.09 – 8.01 (m, 6H, H_{Ar}), 8.01 – 7.90 (m, 6H, H_{Ar}), 7.57 – 7.48 (m, 5H, H_{Ar}), 7.44 – 7.31 (m, 13H, H_{Ar}), 6.61 (d, $^3J_{H1-H2}$ = 2.1, 1H, H-1$_{VI}$), 6.09 (t, $^3J_{H4-H3}$ = $^3J_{H4-H5}$ = 9.8, 1H, H-4$_{VI}$), 5.97 (t, $^3J_{H4-H3}$ = $^3J_{H4-H5}$ = 10.2, 1H, H-4$_{VII}$), 5.87 (dd, $^3J_{H3-H4}$ = 9.7, $^3J_{H3-H2}$ = 3.3, 1H, H-3$_{VI}$), 5.79 (ddd, $^3J_{H3-F}$ = 27.8, $^3J_{H3-H4}$ = 10.2, $^3J_{H3-H2}$ = 2.4, 1H, H-3$_{VII}$), 5.37 (dd, $^3J_{H1-F}$ = 7.6, $^3J_{H1-H2}$ = 1.5, 1H, H-1$_{VII}$), 5.26 – 5.11 (m, 1H, H-2$_{VII}$), 4.73 – 4.61 (m, 4H, H-2$_{VI}$, H-5$_{VI}$, H-6a$_{VI}$, H-6a$_{VII}$), 4.60 – 4.54 (m, 2H, H-5$_{VII}$, H-6b$_{VII}$), 4.47 (dd, $^2J_{H6b-H6a}$ = 12.1, $^3J_{H6b-H5}$ = 5.0, 1H, H-6b$_{VI}$); ^{13}C–NMR (101 MHz, $CDCl_3$) *δ:* 166.3 (CO), 166.3 (CO), 165.7 (CO), 165.5 (CO), 165.4 (CO), 165.4 (CO), 160.0 (CN), 133.9 (C_{Ar}), 133.7 (C_{Ar}), 133.6 (C_{Ar}), 133.2 (C_{Ar}), 133.2 (C_{Ar}), 130.1 (C_{Ar}), 130.0 (C_{Ar}), 130.0 (C_{Ar}), 129.9 (C_{Ar}), 129.8 (C_{Ar}), 129.8 (C_{Ar}), 129.7 (C_{Ar}), 129.1 (C_{Ar}), 128.9 (C_{Ar}), 128.7 (C_{Ar}), 128.6 (C_{Ar}), 128.5 (C_{Ar}), 99.0 (d, $^2J_{C1-F}$ = 30.7, C-1$_{VII}$), 96.2 (C-1$_{VI}$), 90.6 (CCl_3), 86.8 (d, $^1J_{C2-F}$ = 180.8, C-2$_{VII}$), 74.3 (C-2$_{VI}$), 71.8 (C-5$_{VI}$), 71.0 (C-3$_{VI}$), 70.5 (d, $^2J_{C3-F}$ = 17.0, C-3$_{VII}$), 70.2 (C-5$_{VII}$), 66.6 (C-4$_{VI}$), 66.3 (C-4$_{VII}$), 63.2 (C-6$_{VII}$), 63.0 (C-6$_{VI}$); ^{19}F–NMR (376 MHz, $CDCl_3$) *δ:* -203.6; ESI–MS *m/z* calcd. for $C_{56}H_{45}NCl_3O_{16}F$: 1111.2; found: 1134.3 $[M+Na]^+$; ESI–HRMS *m/z* calcd. for $C_{56}H_{45}NCl_3O_{16}FNa$ $[M+Na]^+$: 1134.1680; found: 1134.1674.

Pentasaccharide 11

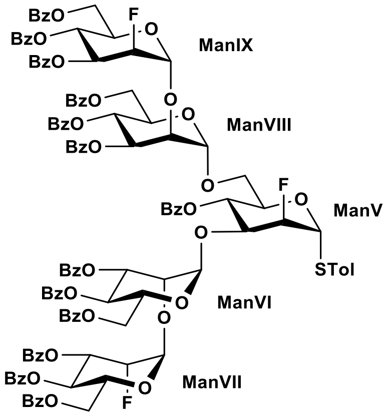

A solution of monosaccharide **5** (96 mg, 0.25 mmol), disaccharide **9** (604 mg, 0.54 mmol) and 4Å MS (powder, 0.8 g) in dry DCM (8 mL) was stirred at -15ºC for 15 min. Then, TMSOTf (13 µL, 0.07 mmol) was added and the reaction mixture was stirred at -15ºC for 1 h. The reaction mixture was quenched with Et_3N (0.1 mL), filtered through Celite and washed with DCM. After removal of the solvent, the crude was purified by silica gel column chromatography (toluene/acetone 40:1) to give pentasaccharide **11** (503 mg, 90%) as a white foam. ^{1}H–NMR (500 MHz, $CDCl_3$) *δ:* 8.12 – 8.07 (m, 4H, H_{Ar}), 8.05 – 8.00 (m, 13H, H_{Ar}), 7.98 – 7.90 (m, 8H, H_{Ar}), 7.55 – 7.27 (m, 42H, H_{Ar}, $H_{o\text{-Tol}}$), 7.15 (d, *J* = 8.1, 2H, $H_{m\text{-Tol}}$), 5.99 – 5.69 (m, 9H, H-3$_{VI}$, H-3$_{VII}$, H-3$_{VIII}$, H-3$_{IX}$, 5 x H-4), 5.48 (dd, $^3J_{H1\text{-}F}$ = 14.3, $^3J_{H1\text{-}H2}$ = 1.4, 1H, H-1$_V$), 5.45 – 5.43 (m, 1H, H-1$_{VI}$), 5.40 – 5.28 (m, 1H, H-2$_V$), 5.22 (d, $^3J_{H1\text{-}H2}$ = 1.5, 1H, H-1$_{VIII}$), 5.17 (dd, $^3J_{H1\text{-}F}$ = 7.4, $^3J_{H1\text{-}H2}$ = 1.7, 1H, H-1$_{VII}$ or H-1$_{IX}$), 5.15 – 5.07 (m, 1H, H-2$_{IX}$), 5.06 – 4.96 (m, 1H, H-2$_{VII}$), 4.85 (dd, $^3J_{H1\text{-}F}$ = 7.6, $^3J_{H1\text{-}H2}$ = 1.6, 1H, H-1$_{VII}$ or H-1$_{IX}$), 4.73 – 4.32 (m, 15H, H-3$_V$, H-2$_{VIII}$, 5 x H-5, 8 x H-6), 4.15 (dd, $^3J_{H2\text{-}H3}$ = 3.3, $^3J_{H2\text{-}H1}$ = 1.9, 1H, H-2$_{VI}$), 4.03 (dd, $^2J_{H6a\text{-}H6b}$ = 10.9, $^3J_{H6a\text{-}H5}$ = 5.7, 1H, H-6a$_V$), 3.68 (dd, $^2J_{H6b\text{-}H6a}$ = 11.3, $^3J_{H6b\text{-}H5}$ = 2.3, 1H, H-6b$_V$), 2.19 (s, 3H, CH_3); ^{13}C–NMR (126 MHz, $CDCl_3$) *δ:* 166.4 (CO), 166.2 (CO), 166.1 (CO), 165.6 (CO), 165.5 (CO), 165.5 (CO), 165.4 (CO), 165.4 (CO), 165.4 (CO), 165.3 (CO), 165.1 (CO), 138.6 ($C_{p\text{-Tol}}$), 133.8 (C_{Ar}), 133.5 (C_{Ar}), 133.4 (C_{Ar}), 133.4 (C_{Ar}), 133.2 (C_{Ar}), 133.1 (C_{Ar}), 132.1 (C_{Ar}), 130.3 (C_{Ar}), 130.1 (C_{Ar}), 130.1 (C_{Ar}), 130.0 (C_{Ar}), 130.0 (C_{Ar}), 129.9 (C_{Ar}), 129.9 (C_{Ar}), 129.8 (C_{Ar}), 129.8 (C_{Ar}), 129.8 (C_{Ar}), 129.7 (C_{Ar}), 129.6 (C_{Ar}), 129.2 (C_{Ar}), 129.2 (C_{Ar}), 129.2 (C_{Ar}), 129.1 (C_{Ar}), 129.1 (C_{Ar}), 129.0 (C_{Ar}), 129.0 (C_{Ar}), 128.9 (C_{Ar}), 128.7 (C_{Ar}), 128.7 (C_{Ar}), 128.6 (C_{Ar}), 128.5 (C_{Ar}), 128.5 (C_{Ar}), 128.5 (C_{Ar}), 128.4 (C_{Ar}), 100.9 (C-1$_{VI}$), 99.4 (d, $^2J_{C1\text{-}F}$ = 24.0, C-1$_{VII}$ or C-1$_{IX}$), 99.2 (d, $^2J_{C1\text{-}F}$ = 24.4, C-1$_{VII}$ or C-1$_{IX}$), 98.5 (C-1$_{VIII}$), 89.9 (d, $^1J_{C2\text{-}F}$ = 189.8, C-2$_V$), 86.9 (d, $^1J_{C2\text{-}F}$ = 180.9, C-2$_{VII}$ or C-2$_{IX}$), 86.7 (d, $^1J_{C2\text{-}F}$ = 180.9, C-2$_{VII}$ or C-2$_{IX}$), 86.2 (d, $^2J_{C1\text{-}F}$ = 22.3, C-1$_V$), 78.6 (d, $^2J_{C3\text{-}F}$ = 18.1, C-3$_V$), 77.4 (C-2$_{VIII}$), 77.3 (C-2$_{VI}$), 71.4, 70.8, 70.6, 70.5 (d, $^2J_{C3\text{-}F}$ = 17.1, C-3$_{VII}$ or C-3$_{IX}$), 70.2 (d, $^2J_{C3\text{-}F}$ =16.7, C-3$_{VII}$ or C-3$_{IX}$), 69.9, 69.8, 69.4, 68.8, 68.2 (C-4$_V$), 67.1 (C-6$_V$), 66.8, 66.5, 63.8, 63.5, 63.1, 21.1 (CH_3); ^{19}F–NMR (376 MHz, $CDCl_3$) *δ:* -187.1 (F_V),

-203.3 (F_{IX}), -203.4 (F_{VII}); ESI–MS *m/z* calcd. for $C_{128}H_{107}SO_{35}F_3$: 2292.6; found: 1164.9 $[M+2NH_4]^{2+}$; ESI–HRMS *m/z* calcd. for $C_{128}H_{107}SO_{35}F_3Na$ $[M+Na]^+$: 2315.6158; found: 2315.6196.

2-Bromoethyl 3,4,6-tri-*O*-acetyl-2-deoxy-2-fluoro-α-D-mannopyranoside (22)

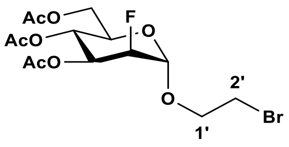

A mixture of trichloroacetimidate derivative **12** (602 mg, 1.33 mmol) and 4Å MS (powder, 2.0 g) in dry DCM (11 mL) was cooled to -20ºC. After 15 min of stirring at -20ºC, 2-bromoethanol (0.13 mL, 1.74 mmol) and TMSOTf (5 µL, 0.03 mmol) were added. The reaction mixture was stirred at -20ºC for 2 h. The reaction was quenched with Et_3N (0.1 mL), filtered through Celite and concentrated under vacuum. The crude was purified by silica gel column chromatography (*n*-hexane/EtOAc 3:1), affording **22** (438 mg, 79%) as a yellow oil. ^{1}H–NMR (400 MHz, $CDCl_3$) *δ:* 5.33 (t, $^3J_{H4-H3} = {}^3J_{H4-H5} = 10.0$, 1H, H-4), 5.25 (ddd, $^3J_{H3-F} = 27.5$, $^3J_{H3-H4} = 10.1$, $^3J_{H3-H2} = 2.6$, 1H, H-3), 5.05 (dd, $^3J_{H1-F} = 7.3$, $^3J_{H1-H2} = 1.8$, 1H, H-1), 4.78 (m, 1H, H-2), 4.25 (dd, $^2J_{H6a-H6b} = 11.8$, $^3J_{H6a-H5} = 5.1$, 1H, H-6a), 4.16 – 4.08 (m, 2H, H-6b, H-5), 4.02 (dt, $^2J_{H1'a-H1'b} = 11.4$, $^3J_{H1'a-H2'} = 5.9$, 1H, H-1'a), 3.90 (dt, $^2J_{H1'b-H1'a} = 11.4$, $^3J_{H1'b-H2} = 5.9$, 1H, H-1'b), 3.51 (t, $^3J_{H2'-H1'a} = {}^3J_{H2'-H1'b} = 5.9$, 2H, H-2'), 2.09 (s, 6H, 2 x CH_3 of OAc), 2.04 (s, 3H, CH_3 of OAc); ^{13}C–NMR (101 MHz, $CDCl_3$) *δ:* 170.8 (CO), 170.2 (CO), 169.6 (CO), 97.5 (d, $^2J_{C1-F} = 29.6$, C-1), 86.8 (d, $^1J_{C2-F} = 179.4$, C-2), 69.9 (d, $^2J_{C3-F} = 16.7$, C-3), 69.2 (C-5), 68.7 (C-1'), 65.8 (C-4), 62.2 (C-6), 29.8 (C-2'), 20.9 (2 x CH_3 of OAc), 20.8 (CH_3 of OAc); ^{19}F–NMR (376 MHz, $CDCl_3$) *δ:* -204.4; ESI–MS *m/z* calcd. for $C_{14}H_{20}BrO_8F$: 414.0; found: 439.0 $[M+Na]^+$; ESI–HRMS *m/z* calcd. for $C_{14}H_{20}{}^{79}BrO_8FNa$ $[M+Na]^+$: 437.0218; found: 437.0230, *m/z* calcd. for $C_{14}H_{20}{}^{81}BrO_8FNa$ $[M+Na]^+$: 439.0197; found: 439.0208.

2-Azidoethyl 3,4,6-tri-*O*-acetyl-2-deoxy-2-fluoro-α-D-mannopyranoside (13)

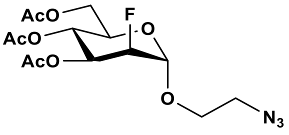

To a solution of monosaccharide **22** (402 mg, 0.97 mmol) in DMF (3 mL), NaN_3 (154 mg, 2.37 mmol) was added, and the reaction mixture was stirred overnight at 50ºC. After evaporation of the solvent, the crude was diluted with DCM (20 mL) and washed with H_2O (2 x 20 mL) and brine (20 mL). The organic phase was dried over anh. $MgSO_4$, filtered and concentrated, to give compound **13** (365 mg, quant.) as

a yellow oil. ^{1}H–NMR (400 MHz, $CDCl_3$) δ: 5.36 (td, $^3J_{H4-H3} = {}^3J_{H4-H5}$ = 10.1, J = 1.6, 1H, H-4), 5.27 (ddd, $^3J_{H3-F}$ = 28.5, $^3J_{H3-H4}$ = 10.1, $^3J_{H3-H2}$ = 2.6, 1H, H-3), 5.05 (dd, $^3J_{H1-F}$ = 7.1, $^3J_{H1-H2}$ = 1.9, 1H, H-1), 4.87 – 4.72 (m, 1H, H-2), 4.28 (dd, $^2J_{H6a-H6b}$ = 12.2, $^3J_{H6a-H5}$ = 5.0, 1H, H-6a), 4.14 (dd, $^2J_{H6b-H6a}$ = 12.4, $^3J_{H6b-H5}$ = 2.4, 1H, H-6b), 4.03 (ddd, $^3J_{H5-H4}$ = 9.9, $^3J_{H5-H6a}$ = 4.9, $^3J_{H5-H6b}$ = 2.4, 1H, H-5), 3.92 (dt, $^2J_{H1'a-H1'b}$ = 10.8, $^3J_{H1'a-H2'}$ = 5.1, 2H, H-1'a), 3.70 (dt, $^2J_{H1'b-H1'a}$ = 10.8, $^3J_{H1'b-H2'}$ = 5.1, 2H, H-1'b), 3.47 (t, $^3J_{H2'-H1'a} = {}^3J_{H2'-H1'b}$ = 5.1, 2H, H-2'), 2.10 (s, 3H, CH_3), 2.09 (s, 6H, 2 x CH_3 of OAc), 2.04 (s, 3H, CH_3 of OAc); ^{13}C–NMR (101 MHz, $CDCl_3$) δ: 170.6 (CO), 170.0 (CO), 169.5 (CO), 97.3 (d, $^2J_{C1-F}$ = 30.2, C-1), 86.7 (d, $^1J_{C2-F}$ = 179.6, C-2), 69.7 (d, $^2J_{C3-F}$ = 17.2, C-3), 68.9 (C-5), 67.2 (C-1'), 65.7 (C-4), 62.1 (C-6), 50.3 (C-2'), 20.7 (2 x CH_3 of OAc), 20.6 (CH_3 of OAc); ^{19}F–NMR (376 MHz, $CDCl_3$) δ: -204.6; ESI–MS *m/z* calcd. for $C_{14}H_{20}N_3O_8F$: 377.1; found: 400.1 $[M+Na]^+$; ESI–HRMS *m/z* calcd. for $C_{14}H_{20}N_3O_8FNa$ $[M+Na]^+$: 400.1127; found: 400.1125.

2-Azidoethyl 2-deoxy-2-fluoro-α-D-mannopyranoside (23)

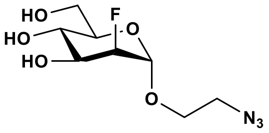

Azide derivative **13** (624 mg, 1.65 mmol) was dissolved in MeOH (11 mL), and NaOMe (56 mg, 0.99 mmol) was added. After 30 min of stirring at r.t., the reaction mixture was neutralized with Amberlite IR-120 (H^+) resin, filtered and concentrated, to give **23** (367 mg, 88%) as a yellow oil. ^{1}H–NMR (400 MHz, CD_3OD) δ: 5.01 (dd, $^3J_{H1-F}$ = 7.6, $^2J_{H1-H2}$ = 1.8, 1H, H-1), 4.69 – 4.53 (m, 1H, H-2), 3.95 (dt, $^2J_{H1'a-H1'b}$ = 10.7, $^3J_{H1'a-H2'}$ = 5.2, 1H, H-1'a), 3.86 (dd, $^2J_{H6a-H6b}$ = 12.0, $^3J_{H6a-H5}$ = 1.6, 1H, H-6a), 3.77 (ddd, $^3J_{H3-F}$ = 31.0, $^3J_{H3-H4}$ = 9.8, $^3J_{H3-H2}$ = 2.6, 1H, H-3), 3.73 – 3.64 (m, 2H, H-1'b, H-6b), 3.64 – 3.59 (m, 2H, H-4, H-5), 3.43 (t, $^3J_{H2'-H1'a} = {}^3J_{H2'-H1'b}$ = 5.0, 2H, H-2'); ^{13}C–NMR (101 MHz, CD_3OD) δ: 98.9 (d, $^2J_{C1-F}$ = 30.1, C-1), 91.0 (d, $^1J_{C2-F}$ = 174.6, C-2), 75.0 (C-4 or C-5), 71.5 (d, $^2J_{C3-F}$ = 17.9, C-3), 68.6 (C-4 or C-5), 67.9 (C-1'), 62.7 (C-6), 51.6 (C-2'); ^{19}F–NMR (376 MHz, CD_3OD) δ: -206.9; ESI–MS *m/z* calcd. for $C_8H_{14}N_3O_5F$: 251.1; found: 274.1 $[M+Na]^+$; ESI–HRMS *m/z* calcd. for $C_{14}H_{20}N_3O_8FNa$ $[M+Na]^+$: 274.0810; found: 274.0813.

2-Azidoethyl 2-deoxy-2-fluoro-4,6-*O*-(phenylmethylene)-α-D-mannopyranoside (14)

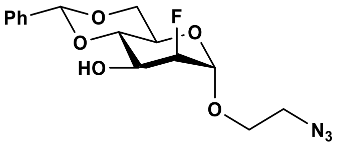

To a solution of unprotected monosaccharide **23** (81 mg, 0.32 mmol) in dry ACN (2 mL), CSA (20 mg, 0.08 mmol) and benzaldehyde dimethyl acetal (0.12 mL, 0.79 mmol) were added. The reaction mixture was stirred at r.t. for 1.5 h and then quenched with Et_3N (0.1 mL). After solvent removal, the resulting crude was purified by silica gel column chromatography (*n*-hexane/EtOAc 2:1), giving **14** (101 mg, 92%) as a yellow oil. ^{1}H–NMR (400 MHz, $CDCl_3$) *δ:* 7.52 – 7.47 (m, 2H, $H_{m\text{-Ph}}$), 7.41 – 7.35 (m, 3H, $H_{o\text{-Ph}}$, $H_{p\text{-Ph}}$), 5.60 (s, 1H, H_{acetal}), 5.04 (dd, $^3J_{H1\text{-}F}$ = 7.9, $^3J_{H1\text{-}H2}$ = 1.7, 1H, H-1), 4.82 (ddd, $^2J_{H2\text{-}F}$ = 49.0, $^3J_{H2\text{-}H3}$ = 2.8, $^3J_{H2\text{-}H1}$ = 1.8, 1H, H-2), 4.3 (dd, $^2J_{H6a\text{-}H6b}$ = 8.7, $^3J_{H6a\text{-}H5}$ = 3.1, 1H, H-6a), 4.16 (dddd, $^3J_{H3\text{-}F}$ = 27.8, $^3J_{H3\text{-}H4}$ = 9.4, $^3J_{H3\text{-}OH}$ = 5.7, $^3J_{H3\text{-}H2}$ = 2.8, 1H, H-3), 3.97 – 3.80 (m, 4H, H-6b, H-1'a, H-4, H-5), 3.69 – 3.63 (m, 1H, H-1'b), 3.47 – 3.41 (m, 2H, H-2'), 2.40 (d, $^3J_{OH\text{-}H3}$ = 5.7, 1H, OH); ^{13}C–NMR (101 MHz, $CDCl_3$) *δ:* 137.1 ($C_{ipso\text{-Ph}}$), 129.4 ($C_{p\text{-Ph}}$), 128.5 ($C_{o\text{-Ph}}$), 126.4 ($C_{m\text{-Ph}}$), 102.4 (C_{acetal}), 98.5 (d, $^2J_{C1\text{-}F}$ = 31.1, C-1), 89.5 (d, $^1J_{C2\text{-}F}$ = 177.5, C-2), 78.8 (C-4), 68.7 (C-6), 67.9 (d, $^2J_{C3\text{-}F}$ = 17.7, C-3), 67.1 (C-1'), 64.0 (C-5), 50.5 (C-2'); ^{19}F–NMR (376 MHz, $CDCl_3$) *δ:* -205.9; ESI–MS *m/z* calcd. for $C_{15}H_{18}N_3O_5F$: 339.1; found: 340.2 $[M+H]^+$; ESI–HRMS *m/z* calcd. for $C_{15}H_{19}N_3O_8F$ $[M+H]^+$: 340.1303; found: 340.1304.

1,2,3,4-Tetra-*O*-benzoyl-α-D-mannopyranoside (24)

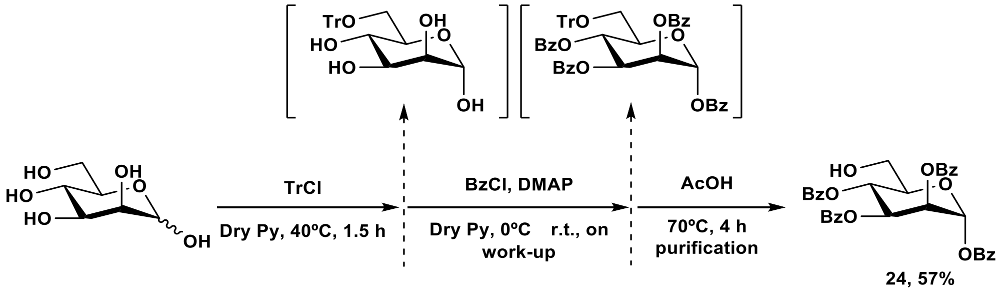

To a solution of D-mannose (1.50 g, 8.33 mmol) in dry Py (15 mL), trityl chloride (2.60 g, 9.16 mmol) was added. After 1.5 h of stirring at 40ºC, the reaction mixture was cooled to 0ºC and BzCl (7.7 mL, 60.61 mmol) and DMAP (cat.) were added. The reaction mixture was stirred overnight at r.t. The mixture was then poured into an ice bath and extracted with DCM (3 x 100 mL). The organic phase was washed with 1 M HCl (100 mL), brine (100 mL) and H_2O (100 mL) and then dried over anh. $MgSO_4$, filtered and concentrated. The crude was dissolved in 80% AcOH (100 mL) and the reaction mixture was stirred at 70ºC for 4 h. After removal of the solvent, the crude was purified by silica gel column chromatography (*n*-hexane/EtOAc 3:1→1:1) to afford **24** (2.82 g, 57%) as a white foam. ^{1}H–NMR (400 MHz, $CDCl_3$) *δ:* 8.19 (d, *J* = 8.0, 2H, H_{Ar}), 8.13 (d, *J* = 8.0, 2H, H_{Ar}), 8.00 (d, *J* = 8.0, 2H, H_{Ar}), 7.85 (d, *J* = 8.1, 2H, H_{Ar}), 7.70 – 7.61 (m, 2H, H_{Ar}), 7.59 – 7.49 (m, 5H, H_{Ar}), 7.46 (t, *J* = 7.4, 1H, H_{Ar}), 7.40 (t, *J* = 8.0, 2H, H_{Ar}), 7.30 (t, *J* = 7.9, 2H, H_{Ar}), 6.62 (d, $^3J_{H1\text{-}H2}$ = 1.7, 1H, H-1), 6.12 (dd, $^3J_{H3\text{-}H4}$ = 10.2, $^3J_{H3\text{-}H2}$ = 3.3, 1H, H-3), 6.01 (t, $^3J_{H4\text{-}H3}$ = $^3J_{H4\text{-}H5}$ =10.1, 1H, H-4), 5.88 (dd, $^3J_{H2\text{-}H3}$ = 3.4, $^3J_{H2\text{-}H1}$ = 2.0, 1H, H-2), 4.24 (m, 1H, H-5), 3.87 (ddd, $^2J_{H6a\text{-}H6b}$ = 12.9, $^3J_{H6a\text{-}OH}$ = 8.2, $^3J_{H6a\text{-}H5}$ = 1.9, 1H, H-6a), 3.78 (ddd,

${}^2J_{H6b-H6a}$ = 13.0, ${}^3J_{H6b-OH}$ = 6.4, ${}^3J_{H6b-H5}$ = 4.1, 1H, H-6b), 2.54 (dd, ${}^3J_{OH-H6a}$ = 8.1, ${}^3J_{OH-H6b}$ = 6.3, 1H, OH); ^{13}C–NMR (101 MHz, $CDCl_3$) *δ:* 166.2 (CO), 165.7 (CO), 165.3 (CO), 164.0 (CO), 134.1 (C_{Ar}), 133.8 (C_{Ar}), 133.5 (C_{Ar}), 130.2 (C_{Ar}), 130.1 (C_{Ar}), 129.9 (C_{Ar}), 129.8 (C_{Ar}), 129.0 (C_{Ar}), 128.9 (C_{Ar}), 128.8 (C_{Ar}), 128.8 (C_{Ar}), 128.7 (C_{Ar}), 128.6 (C_{Ar}), 128.5 (C_{Ar}), 91.5 (C-1), 73.6 (C-5), 69.7 (C-2), 69.6 (C-3), 66.6 (C-4), 61.2 (C-6); ESI–MS *m/z* calcd. for $C_{34}H_{28}O_{10}$: 596.2; found: 614.2 $[M+NH_4]^+$; ESI–HRMS *m/z* calcd. for $C_{34}H_{28}O_{10}Na$ $[M+Na]^+$:619.1575; found 619.1575.

1,2,3,4-Tetra-*O*-benzoyl-6-deoxy-6-fluoro-α-D-mannopyranoside (15)

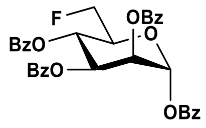

To a solution of monosaccharide **24** (3.99 g, 6.70 mmol) in dry DCM (80 mL) cooled to -40ºC, DAST (5.6 mL, 40.23 mmol) was added dropwise. The reaction mixture was stirred at r.t for 3 days. After cooling to -20ºC, MeOH (12 mL) was slowly added and the solvent was removed. The resulting crude was diluted with DCM (150 mL) and washed with sat. $NaHCO_3$ aq. soln. (2 x 100 mL) and H_2O (2 x 100 mL). The organic phase was dried over anh. $MgSO_4$, filtered and concentrated. The crude was purified by silica gel column chromatography (*n*-hexane/EtOAc 3:1), giving 6-fluorinated compound **15** (2.00 g, 50%; 70% based on recovered starting material) as a white foam. ^{1}H–NMR (400 MHz, $CDCl_3$) *δ:* 8.20 (d, *J* = 7.8, 2H, H_{Ar}), 8.12 (d, *J* = 7.9, 2H, H_{Ar}), 7.99 (d, *J* = 8.2, 2H, H_{Ar}), 7.85 (d, *J* = 8.1, 2H, H_{Ar}), 7.71 – 7.61 (m, 2H, H_{Ar}), 7.59 – 7.49 (m, 5H, H_{Ar}), 7.46 (t, *J* = 7.4, 1H, H_{Ar}), 7.40 (t, *J* = 8.0, 2H, H_{Ar}), 7.29 (t, *J* = 7.9, 2H, H_{Ar}), 6.63 (d, ${}^3J_{H1-H2}$ = 2.0, 1H, H-1), 6.12 – 6.03 (m, 2H, H-4, H-3), 5.88 (dd, ${}^3J_{H2-H3}$ = 3.1, ${}^3J_{H2-H1}$ = 2.1, 1H, H-2), 4.72 – 4.56 (m, 2H, H-6), 4.52 – 4.40 (m, 1H, H-5); ^{13}C–NMR (101 MHz, $CDCl_3$) *δ:* 165.7 (CO), 165.3 (CO), 165.3 (CO), 163.8 (CO), 134.2 (C_{Ar}), 133.8 (C_{Ar}), 133.7 (C_{Ar}), 133.5 (C_{Ar}), 130.2 (C_{Ar}), 130.1 (C_{Ar}), 129.9 (C_{Ar}), 129.8 (C_{Ar}), 129.0 – 128.4 (C_{Ar}), 91.34 (C-1), 81.2 (d, ${}^1J_{C6-F}$ = 176.4, C-6), 72.0 (d, ${}^2J_{C5-F}$ = 19.3, C-5), 69.9 (C-3), 69.4 (C-2), 65.7 (d, ${}^3J_{C4-F}$ = 6.8, C-4); ^{19}F–NMR (376 MHz, $CDCl_3$) *δ:* -233.0; ESI–MS *m/z* calcd. for $C_{34}H_{27}O_9F$: 598.2; found: 616.2 $[M+NH_4]^+$; ESI–HRMS *m/z* calcd. for $C_{34}H_{27}O_9FNa$ $[M+Na]^+$: 621.1531; found 621.1532.

2,3,4-Tri-*O*-benzoyl-6-deoxy-6-fluoro-α-D-mannopyranosyl trichloroacetimidate (16)

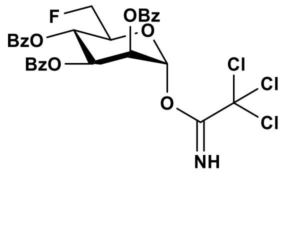

Fluorine derivative **15** (1.00 g, 1.67 mmol) was dissolved in dry DMF (15 mL) and N_2H_4·AcOH (190 mg, 2.00 mmol) was added. The reaction mixture was stirred at 50ºC for 2 h. After removal of the solvent, the crude was diluted with EtOAc (40 mL) and washed with H_2O (40 mL) and brine (40 mL). The organic phase was dried over anh. $MgSO_4$, filtered and concentrated. The crude was purified by silica gel column chromatography (*n*-hexane/EtOAc 3:1), giving the corresponding hemiacetal (0.58 g, 70%) as a white foam. To a solution of the resulting hemiacetal in dry DCM (18 mL) cooled to 0ºC, trichloroacetonitrile (0.6 mL, 5.86 mmol) and DBU (70 µL, 0.47 mmol) were added. The reaction mixture was stirred at r.t. for 1 h. After removal of the solvent, the crude was purified by silica gel column chromatography (*n*-hexane/EtOAc 4:1) to afford trichloroacetimidate **16** (516 mg, 69%) as a yellowish foam. ^{1}H–NMR (400 MHz, $CDCl_3$) *δ:* 8.89 (s, 1H, NH), 8.11 (d, *J* = 8.4, 2H, H_{Ar}), 7.98 (d, *J* = 8.3, 2H, H_{Ar}), 7.83 (d, *J* = 8.2, 2H, H_{Ar}), 7.64 (t, *J* = 7.4, 1H, H_{Ar}), 7.57 – 7.49 (m, 3H, H_{Ar}), 7.47 – 7.37 (m, 3H, H_{Ar}), 7.29 (d, *J* = 8.0, 2H, H_{Ar}), 6.58 (d, $^3J_{H1-H2}$ = 1.9, 1H, H-1), 6.05 (t, $^3J_{H4-H3}$ = $^3J_{H4-H5}$ = 10.1, 1H, H-4), 5.97 (dd, $^3J_{H3-H4}$ = 10.2, $^3J_{H3-H2}$ = 3.3, 1H, H-3), 5.91 (dd, $^3J_{H2-H3}$ = 3.3, $^3J_{H2-H1}$ = 2.1, 1H, H-2), 4.72 – 4.58 (m, 2H, H-6), 4.52 – 4.41 (m, 1H, H-5); ^{13}C–NMR (101 MHz, $CDCl_3$) *δ:* 165.6 (CO), 165.5 (CO), 165.4 (CO), 159.9 (CN), 133.9 (C_{Ar}), 133.8 (C_{Ar}), 133.5 (C_{Ar}), 130.2 (C_{Ar}), 130.0 (C_{Ar}), 129.9 (C_{Ar}), 129.0 (C_{Ar}), 128.9 (C_{Ar}), 128.8 (C_{Ar}), 128.7 (C_{Ar}), 128.5 (C_{Ar}), 94.8 (C-1), 82.1 (CCl_3), 81.2 (d, $^1J_{C6-F}$ = 177.1, C-6), 72.4 (d, $^2J_{C5-F}$ = 19.0, C-5), 69.8 (C-3), 68.8 (C-2), 65.6 (d, $^3J_{C4-F}$ = 6.6, C-4); ^{19}F–NMR (376 MHz, $CDCl_3$) *δ:* -232.7; ESI–MS *m/z* calcd. for $C_{29}H_{23}NCl_3O_8F$: 637.0; found: 662.1 $[M+Na]^+$; ESI–HRMS *m/z* calcd. for $C_{29}H_{23}NCl_3O_8FNa$ $[M+Na]^+$: 660.0366; found: 660.0368.

Trisaccharide 18

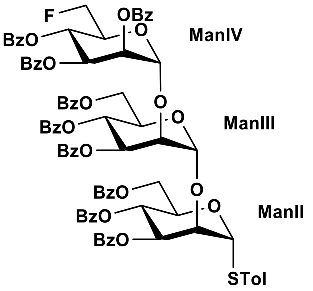

A solution of donor **16** (330 mg, 0.52 mmol), acceptor **17** (428 mg, 0.40 mmol) and 4Å MS (powder, 1.6 g) in dry DCM (16 mL) was stirred at -10ºC for 15 min. Then, TMSOTf (11 µL, 0.06 mmol) was added, and the reaction mixture was stirred at -10ºC for 45 min. The reaction was neutralized with Et_3N (1 mL), filtered through Celite and washed with DCM. After removal of the solvent, the crude was purified by silica gel column chromatography (toluene/EtOAc 30:1) to give the trisaccharide **18** (471 mg, 77%) as a white foam. ^{1}H–NMR (400 MHz, $CDCl_3$) *δ:* 8.10 – 7.92 (m, 14H, H_{Ar}), 7.87 – 7.82 (m, 4H, H_{Ar}), 7.61, (t, *J* = 7.5, 1H, H_{Ar}), 7.52 – 7.21 (m, 26H, $H_{o\text{-Tol}}$, H_{Ar}), 7.13 (t, *J* = 7.3, 1H, H_{Ar}), 6.96 (d, *J* = 8.1, 1H, $H_{m\text{-Tol}}$), 6.05 (t, $^3J_{H4-H3}$ = $^3J_{H4-H5}$ = 10.1, 1H, H-4_{III}), 5.98 (t, $^3J_{H4-H3}$ = $^3J_{H4-H5}$ = 9.2, 1H, H-4_{II}),

5.95 (dd, $^{3}J_{H3\text{-}H4}$ = 10.3, $^{3}J_{H3\text{-}H2}$ = 3.3, 1H, H-3$_{IV}$), 5.90 (dd, $^{3}J_{H3\text{-}H4}$ = 10.2, $^{3}J_{H3\text{-}H2}$ = 3.3, 1H, H-3$_{III}$), 5.80 – 5.72 (m, 4H, H-3$_{II}$, H-4$_{IV}$, H-1$_{II}$, H-2$_{IV}$), 5.43 (d, $^{3}J_{H1\text{-}H2}$ = 1.4, 1H, H-1$_{III}$), 5.01 – 4.93 (m, 2H, H-5$_{II}$, H-1$_{IV}$), 4.73 – 4.70 (m, 1H, H-2$_{II}$), 4.68 – 4.44 (m, 6H, H-6$_{II}$, H-2$_{III}$, H-6$_{III}$, H-5$_{IV}$), 4.39 – 4.17 (m, 3H, H-5$_{IV}$, H-6$_{IV}$), 2.26 (s, 3H, CH_3); ^{13}C–NMR (101 MHz, $CDCl_3$) *δ:* 166.4 (CO), 166.3 (CO), 165.8 (CO), 165.6 (CO), 165.5 (CO), 165.4 (CO), 165.3 (CO), 165.0 (CO), 164.9 (CO), 138.4 (C_{Ar}), 133.6 (C_{Ar}), 133.6 (C_{Ar}), 133.5 (C_{Ar}), 133.4 (C_{Ar}), 133.2 (C_{Ar}), 133.1 (C_{Ar}), 133.1 (C_{Ar}), 132.7 (C_{Ar}), 130.3 – 129.7 (C_{Ar}), 129.3 (C_{Ar}), 129.3 (C_{Ar}), 129.2 (C_{Ar}), 129.1 (C_{Ar}), 129.0 (C_{Ar}), 128.9 (C_{Ar}), 128.8 – 128.3 (C_{Ar}), 100.2 (C-1$_{III}$), 99.7 (C-1$_{IV}$), 87.1 (C-1$_{II}$), 81.3 (d, $^{1}J_{C6\text{-}F}$ = 175.6, C-6$_{IV}$), 77.7 (C-2$_{III}$), 77.3 (C-2$_{II}$), 71.6 (C-3$_{III}$), 70.4 (d, $^{2}J_{C5\text{-}F}$ = 18.1, C-5$_{IV}$), 70.3, 70.0, 69.8, 69.8, 69.6, 67.7 (C-4$_{II}$), 67.2 (C-4$_{III}$), 65.9 (d, $^{3}J_{C4\text{-}F}$ = 7.4, C-4$_{IV}$), 63.7 (C-6$_{II}$), 63.6 (C-6$_{III}$), 21.2 (CH_3); ^{19}F–NMR (376 MHz, $CDCl_3$) *δ:* -231.2; ESI–MS *m/z* calcd. for $C_{88}H_{73}SO_{23}F$: 1548.4; found: 1566.6 $[M+NH_4]^+$; ESI–HRMS *m/z* calcd. for $C_{88}H_{73}SO_{23}FNa$ $[M+Na]^+$: 1571.4140; found: 1571.4172.

Tetrasaccharide 19

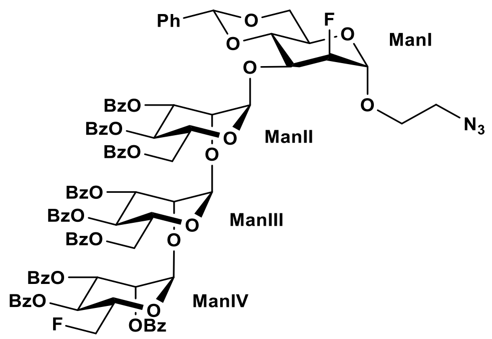

A solution of trisaccharide **18** (84 mg, 0.05 mmol), monosaccharide **14** (14 mg, 0.04 mmol) and 4Å MS (powder, 0.3 g) in dry DCM (2.5 mL) was stirred at -20ºC for 15 min. Then, NIS (27 mg, 0.11 mmol) and TfOH (2 µL, 0.02 mmol) were added, and the reaction was stirred at -20ºC for 1.5 h. The reaction was quenched with sat. $NaHCO_3$ aq. soln. (0.2 mL), filtered through Celite and washed with DCM. The organic phase was washed with sat. $Na_2S_2O_3$ aq. soln. (10 mL) and then dried over anh. $MgSO_4$, filtered and concentrated. Purification of the crude by silica gel column chromatography (*n*-hexane/EtOAc 2:1) gave tetrasaccharide **19** (55 mg, 75%) as a white foam. ^{1}H–NMR (500 MHz, $CDCl_3$) *δ:* 8.14 – 8.07 (m, 4H, H_{Ar}), 8.04 (d, *J* = 7.9, 2H, H_{Ar}), 8.00 (d, *J* = 8.3, H_{Ar}), 7.97 – 7.90 (m, 6H, H_{Ar}), 7.85 (d, *J* = 7.9, 2H, H_{Ar}), 7.79 (d, *J* = 8.2, 2H, H_{Ar}), 7.60 (t, *J* = 7.5, 1H H_{Ar}), 7.54 – 7.08 (m, 36H, H_{Ar}), 6.88 (t, *J* = 7.5, 1H, H_{Ar}), 6.06 – 5.84 (m, 5H, H-4$_{III}$, H-4$_{II}$, H-3$_{IV}$, H-3$_{III}$, H-3$_{II}$), 5.81 – 5.72 (m, 2H, H-4$_{IV}$, H-2$_{IV}$), 5.59 – 5.57 (m, 1H, H-1$_{II}$), 5.54 (br s, 1H, H_{acetal}), 5.44 – 5.42 (m, 1H, H-1$_{III}$), 5.01 – 4.85 (m, 3H, H-1$_{I}$, H-2$_{I}$, H-1$_{IV}$), 4.71 – 4.66 (m, 1H, H-6a$_{II}$), 4.60 – 4.53 (m, 3H, H-6b$_{II}$, H-2$_{II}$, H-5$_{II}$), 4.47 – 4.12 (m, 10H, H-3$_{I}$, H-4$_{I}$, H-6a$_{I}$, H-2$_{III}$, H-5$_{III}$, H-6$_{III}$, H-5$_{IV}$, H-6$_{IV}$), 3.94 – 3.75 (m, 3H, H-5$_{I}$, H-1'a, H-6b$_{I}$), 3.56 (ddd, $^{2}J_{H1'b\text{-}H1'a}$ = 10.6, $^{3}J_{H1'b\text{-}H2'}$ = 6.4, $^{3}J_{H1'b\text{-}H2'}$ = 4.0, 1H, H-1'b), 3.36 – 3.31 (m, 1H, H-2'a), 3.30 – 3.27 (m, 1H,

H-2'b); ^{13}C–NMR (126 MHz, $CDCl_3$) *δ:* 166.3 (CO), 166.3 (CO), 165.8 (CO), 165.6 (CO), 165.5 (CO), 165.5 (CO), 165.3 (CO), 165.0 (CO), 164.9 (CO), 138.0 (C_{Ar}), 137.1 (C_{Ar}), 133.6 (C_{Ar}), 133.5 (C_{Ar}), 133.5 (C_{Ar}), 133.4 (C_{Ar}), 133.4 (C_{Ar}), 133.2 (C_{Ar}), 133.1 (C_{Ar}), 133.0 (C_{Ar}), 130.2 – 129.8 (C_{Ar}), 129.3 (C_{Ar}), 129.3 (C_{Ar}), 129.2 (C_{Ar}), 129.2 (C_{Ar}), 129.1 (C_{Ar}), 129.0 (C_{Ar}), 128.8 (C_{Ar}), 128.7 – 128.4 (C_{Ar}), 128.36 (C_{Ar}), 128.28 (C_{Ar}), 101.9 (C_{acetal}), 100.2 (C-1_{I}), 99.8 (C-1_{III} and C-1_{IV}), 98.4 (d, $^{2}J_{C1-F}$ = 30.8, C-1_{I}), 89.1 (d, $^{1}J_{C2-F}$ = 180.3, C-2_{I}), 81.3 (d, $^{1}J_{C6-F}$ = 174.6, C-6_{IV}), 77.9 (C-4_{I}), 77.4 (C-2_{III}), 76.2 (C-2_{II}), 73.5 (d, $^{2}J_{C3-F}$ = 14.8, C-3_{I}), 71.0 (C-3_{II}), 70.5 (C-3_{IV}), 70.4 (d, $^{2}J_{C5-F}$ = 19.1, C-5_{IV}), 70.1 (C-2_{IV}), 69.7 (C-3_{III}), 69.6 (C-5_{II}, C-5_{III}), 68.7 (C-6_{I}), 67.8 (C-4_{II}), 67.3 (C-1'), 67.2 (C-4_{III}), 65.9 (d, $^{3}J_{C4-F}$ = 7.4, C-4_{IV}), 64.3 (C-5_{I}), 63.8 (C-6_{II}), 63.3 (C-6_{III}), 50.3 (C-2'); ^{19}F–NMR (376 MHz, $CDCl_3$) *δ:* -202.0 (F_{I}), -231.5 (F_{IV}); ESI–MS *m/z* calcd. for $C_{96}H_{83}N_3O_{28}F_2$: 1763.5; found: 1782.5 $[M+NH_4]^+$; ESI–HRMS *m/z* calcd. for $C_{96}H_{83}N_3O_{28}F_2Na$ $[M+Na]^+$: 1787.5023; found: 1787.5054.

Tetrasaccharide 20

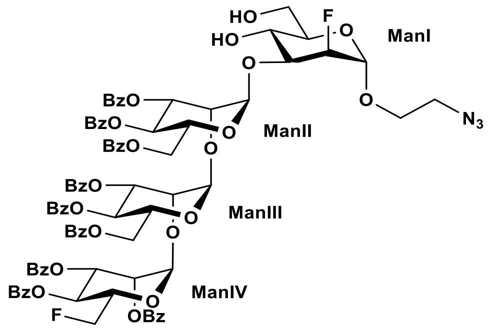

PTSA·H_2O (122 mg, 0.63 mmol) was added to a stirred solution of tetrasaccharide **19** (556 mg, 0.32 mmol) in ACN (20 mL). After 2.5 h of stirring at r.t., the reaction mixture was quenched with Et_3N (50 µL) and concentrated under vacuum. Purification of the crude by silica gel column chromatography (DCM/MeOH 50:1) afforded tetrasaccharide **20** (486 mg, 92%) as a white foam. ^{1}H–NMR (400 MHz, $CDCl_3$) *δ:* 8.10 – 7.91 (m, 14H, H_{Ar}), 7.89 – 7.33 (m, 4H, H_{Ar}), 7.61 (t, *J* = 7.4, 1H, H_{Ar}), 7.52 – 7.23 (m, 21H, H_{Ar}), 7.20 – 7.15 (m, 5H, H_{Ar}), 6.02 (t, $^{3}J_{H4-H3}$ = $^{3}J_{H4-H5}$ = 9.8, 1H, H-4_{III}), 5.96 – 5.90 (m, 2H, H-3_{III}, H-3_{IV}), 5.87 – 5.78 (m, 4H, H-4_{II}, H-4_{IV}, H-2_{IV}, H-3_{II}), 5.48 (d, $^{3}J_{H1-H2}$ = 1.5, 1H, H-1_{III}), 5.45 (d, $^{3}J_{H1-H2}$ = 3.1, 1H, H-1_{II}), 5.07 (br s, 1H, H-1_{IV}), 5.01 – 4.85 (m, 1H, H-2_{I}), 4.72 (dd, $^{3}J_{H1-F}$ = 7.3, $^{3}J_{H1-H2}$ = 1.7, 1H, H-1_{I}), 4.69 – 4.48 (m, 7H, H-2_{II}, H-5_{II}, H-6_{II}, H-5_{III}, H-6_{III}), 4.44 – 4.26 (m, 4H, H-2_{III}, H-5_{IV}, H-6_{IV}), 4.16 – 4.00 (m, 2H, H-4_{I}, H-3_{I}), 3.94 – 3.78 (m, 3H, H-6_{I}, H-1'a), 3.73 (m, 1H, H-5_{I}), 3.52 – 3.45 (m, 1H, H-1'b), 3.31 (m, 2H, H-2'); ^{13}C–NMR (101 MHz, $CDCl_3$) *δ:* 166.9 (CO), 166.3 (CO), 165.9 (CO), 165.7 (CO), 165.6 (CO), 165.5 (2 CO), 165.1 (CO), 165.0 (CO), 133.7 – 133.1 (C_{Ar}), 132.4 (C_{Ar}), 132.3 (C_{Ar}), 132.3 (C_{Ar}), 130.2 – 129.8 (C_{Ar}), 129.7 (C_{Ar}), 129.3 (C_{Ar}), 129.3 (C_{Ar}), 129.1 (C_{Ar}), 129.0 (C_{Ar}), 128.9 (C_{Ar}), 128.8 (C_{Ar}), 128.7 – 128.4 (C_{Ar}), 101.2 (C-1_{II}), 99.6 (C-1_{IV}), 98.8 (C-1_{III}), 97.7 (d, $^{2}J_{C1-F}$ = 29.6, C-1_{I}), 88.6 (d, $^{1}J_{C2-F}$ = 178.4, C-2_{I}), 81.4 (d, $^{1}J_{C6-F}$ = 174.4, C-6_{IV}), 79.4 (d, $^{2}J_{C3-F}$ = 16.7,

C-3$_{I}$), under $CDCl_3$ (C-2$_{III}$), 74.7 (C-2$_{II}$), 72.8 (C-5$_{IV}$), 70.7 (C-5$_{IV}$), 70.6, 70.5, 70.4, 70.1, 69.7, 69.6, 69.5, 67.9 (C-4$_{III}$), 67.0 (C-1'), 66.8 (C-4$_{I}$), 66.0, 66.0, 63.9 (C-6$_{II}$ and C-6$_{III}$), 62.5 (C-6$_{I}$), 50.4 (C-2'); ^{19}F–NMR (376 MHz, $CDCl_3$) *δ:* -203.2 (F$_{I}$), -232.1 (F$_{IV}$); ESI–MS *m/z* calcd. for $C_{89}H_{79}N_3O_{28}F_2$: 1675.5; found: 1694.5 $[M+NH_4]^+$; ESI–HRMS *m/z* calcd. for $C_{89}H_{79}N_3O_{28}F_2Na$ $[M+Na]^+$: 1698.4710; found: 1698.4693.

Nonasaccharide 21

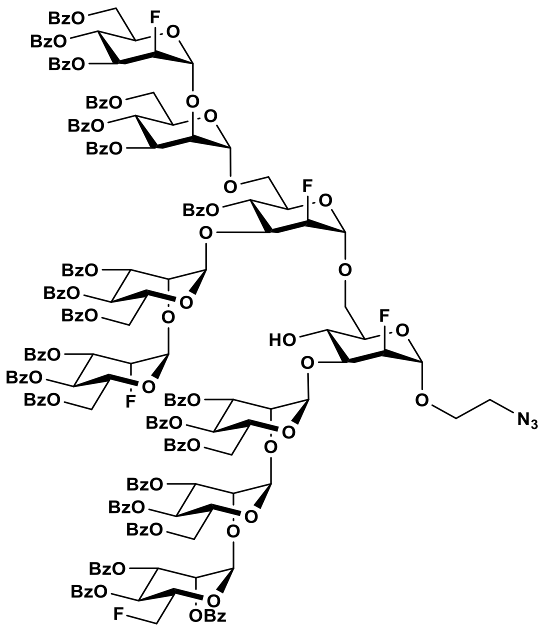

A solution of acceptor **20** (287 mg, 0.17 mmol), donor **11** (503 mg, 0.22 mmol) and 4Å MS (powder, 1.2 g) in dry DCM (12 mL) was stirred at -20ºC for 15 min. Then, NIS (58 mg, 0.11 mmol) and TfOH (11.1 µL, 0.10 mmol) were added. The reaction mixture was allowed to warm to r.t., and stirred overnight. The reaction was quenched with sat. $NaHCO_3$ aq. soln. (1 mL), filtered through Celite and washed with DCM. The organic phase was washed with sat. $Na_2S_2O_3$ aq. soln. (30 mL) and then dried over anh. $MgSO_4$, filtered and concentrated. The crude was purified by silica gel column chromatography (toluene/acetone 16:1) to give nonasaccharide **21** (269 mg, 41%) as a white foam. ^{1}H–NMR (500 MHz, $CDCl_3$) *δ:* 8.10 – 7.82 (m, 42H, H$_{Ar}$), 7.77 (d, *J* = 7.9, 2H, H$_{Ar}$), 7.61 (t, *J* = 7.4, 1H, H$_{Ar}$), 7.55 – 7.23 (m, 62H, H$_{Ar}$), 7.18 (t, *J* = 7.2, 2H, H$_{Ar}$), 7.11 – 7.05 (m, 1H, H$_{Ar}$), 6.09 (t, $^3J_{H4\text{-}H3}$ = $^3J_{H4\text{-}H5}$ = 9.9, 1H, H-4), 6.02 – 5.86 (m, 8H), 5.84 – 5.60 (m, 7H), 5.48 – 5.44 (m, 1H, H-1), 5.42 – 5.37 (m, 2H, H-1), 5.28 – 5.16 (m, 1H, H-2), 5.28 – 5.22 (m, 2H, H-1), 5.19 – 5.06 (m, 1H, H-2), 5.12 – 5.06 (m, 1H, H-1), 5.12 – 4.99 (m, 1H, H-2), 5.04 – 4.91 (m, 1H, H-2), 5.04 – 4.91 (m, 1H, H-2), 4.97 – 4.91 (m, 1H, H-2), 4.87 – 4.82 (m, 1H, H-1), 4.78 – 4.73 (m, 1H, H-1), 4.71 – 3.91 (m, 32H), 3.84 – 3.78 (m, 1H, H-1'a), 3.66 – 3.60 (m, 1H, H-6b), 3.54 – 3.47 (m, 1H, H-1'b), 3.30 – 3.20 (m, 2H, H-2'); ^{13}C–NMR (126 MHz, $CDCl_3$) *δ:* 166.6 (CO), 166.4 (CO), 166.3 (CO), 166.2 (CO), 166.2 (CO), 166.2 (CO), 166.0 (CO), 165.7

(CO), 165.6 (CO), 165.6 (CO), 165.5 (CO), 165.5 (CO), 165.5 (CO), 165.4 (CO), 165.4 (CO), 165.4 (CO), 165.1 (CO), 165.1 (CO), 164.9 (CO), 138.0 (C_{Ar}), 136.0 (C_{Ar}), 133.8 (C_{Ar}), 133.6 (C_{Ar}), 133.6 – 133.1 (C_{Ar}), 130.2 – 129.6 (C_{Ar}), 129.3 (C_{Ar}), 129.2 (C_{Ar}), 129.2 (C_{Ar}), 129.1 (C_{Ar}), 129.1 (C_{Ar}), 129.1 (C_{Ar}), 129.1 (C_{Ar}), 129.0 (C_{Ar}), 129.0 (C_{Ar}), 128.8 (C_{Ar}), 128.7 (C_{Ar}), 128.7 – 128.4 (C_{Ar}), 128.3 (C_{Ar}), 125.4 (C_{Ar}), 101.6 (C-1), 100.6 (C-1), 100.0 (C-1), 99.8 (C-1), 99.5 (d, $^{2}J_{C1\text{-}F}$ = 31.0, C-1), 99.1 (d, $^{2}J_{C1\text{-}F}$ = 31.0, C-1), 98.1 (C-1), 97.5 (d, $^{2}J_{C1\text{-}F}$ = 29.5, C-1), 88.4 (d, $^{1}J_{C2\text{-}F}$ = 177.3, C-2), 88.4 (d, $^{1}J_{C2\text{-}F}$ = 180.4, C-2), 86.9 (d, $^{1}J_{C2\text{-}F}$ = 180.04, C-2), 86.7 (d, $^{1}J_{C2\text{-}F}$ = 180.8, C-2), 82.0, 81.3 (d, $^{1}J_{C6\text{-}F}$ = 174.5, C-6), 80.4 (d, $^{2}J_{C3\text{-}F}$ = 17.3, C-3), 77.9, 77.8, 77.7, 76.1, 72.3, 71.3, 71.3, 70.8, 70.7, 70.6, 70.5, 70.5 – 70.3, 70.3, 70.1, 69.9, 69.8, 69.7, 69.6, 69.4, 68.9, 68.3, 67.7, 67.3, 67.1, 67.1, 67.0, 66.5, 66.3, 66.2, 66.2, 66.0, 65.9, 63.7, 63.6, 63.3, 63.2, 62.9, 50.3 (C-2'); ^{19}F–NMR (471 MHz, $CDCl_3$) *δ:* -202.4 (F_I), -202.6 (F_V), -203.2 (F_{IX}), -203.3 (F_{VII}), -230.4 (F_I); ESI–MS *m/z* calcd. for $C_{210}H_{178}N_3O_{63}F_5$: 3844.1; found: 1941.0 $[M+Na+NH_4]^{2+}$; ESI–HRMS *m/z* calcd. for $C_{210}H_{178}N_3O_{63}F_5Na$ $[M+Na]^+$: 3867.0629; found: 3867.0881.

Nonasaccharide 1

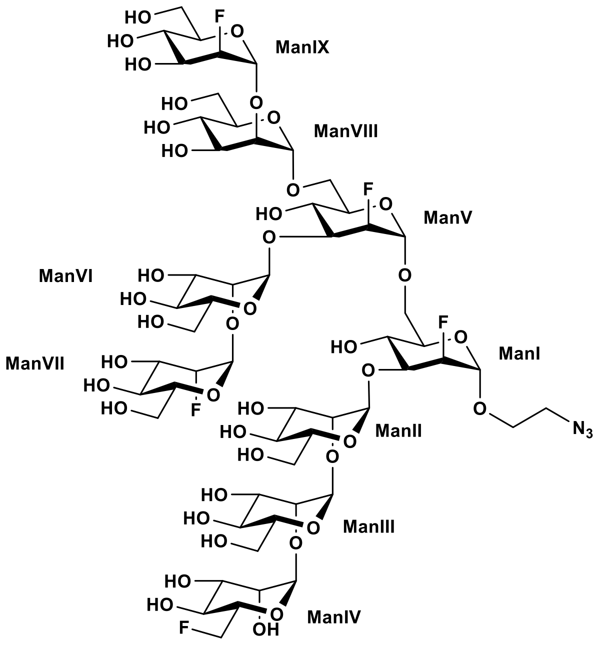

To a solution of nonasaccharide **21** (100 mg, 0.026 mmol) in MeOH/toluene (4:1, 3.5 mL), NaOMe (41 mg, 0.721 mmol) was added. The reaction mixture was stirred overnight at r.t., then was neutralized with Amberlite IR-120 (H^+) resin, filtered and concentrated *in vacuo*. The crude was purified by size-exclusion chromatography (Sephadex G25, H_2O/MeOH 9:1) to give unprotected nonasaccharide **1** (33 mg, 82%) as a white foam. ^{1}H–NMR (600 MHz, D_2O) *δ:* 5.50 – 5.48 (m, 1H, H-1_{VI}), 5.41 – 5.39 (m, 1H, H-1_{II}), 5.36 (dd, $^{3}J_{H1\text{-}F}$ = 7.7, $^{3}J_{H1\text{-}H2}$ = 1.7, 1H, H-1_{VII}), 5.34 (dd, $^{3}J_{H1\text{-}F}$ = 7.7, $^{3}J_{H1\text{-}H2}$ = 1.7, 1H, H-1_{IX}), 5.31 (d, $^{3}J_{H1\text{-}H2}$ = 1.6, 1H, H-1_{III}), 5.23 (d, $^{3}J_{H1\text{-}H2}$ = 1.5, 1H, H-1_{VIII}), 5.22 – 5.19 (m, 2H, H-1_I, H-

1_V), 5.14 (d, $^3J_{H1-H2}$ = 1.6, 1H, H-1_{IV}), 5.11 – 5.00 (m, 2H, H-2_I, H-2_{II}), 5.03 – 4.92 (m, 2H, H-2_{IX}, H-2_{VII}), under H_2O (2H, H-6_{IV}), 4.23 (dd, $^3J_{H2-H3}$ = 3.3, $^3J_{H2-H1}$ = 1.8, 1H, H-2_{VI}), 4.20 – 4.10 (m, 6H), 4.08 – 3.75 (m, 45H), 3.62 (m, 1H, H-2'a), 3.57 (m, 1H, H-2'b); ^{13}C–NMR (126 MHz, D_2O) δ: 102.3 (C-1_{IV}), 100.9 (C-1_{II}), 100.6 (C-1_{III}), 100.5 (C-1_{VI}), 99.3 (d, $^2J_{C1-F}$ = 30.1, C-1_{VII}), 99.2 (d, $^2J_{C1-F}$ = 30.1, C-1_{IX}), 98.0 (C-1_{VIII}), 97.1 (d, J_{C-F} = 29.6, C-1_I or C-1_V), 96.7 (d, J_{C-F} = 29.5, C-1_I or C-1_V), 89.4 (d, $^1J_{C2-F}$ = 172.6, C-2_{IX} or C-2_{VII}), 89.4 (d, $^1J_{C2-F}$ = 172.1, C-2_{IX} or C-2_{VII}), 88.8 (d, $^1J_{C2-F}$ = 174.0, C-2_I or C-2_V), 88.7 (d, $^1J_{C2-F}$ = 173.6, C-2_I or C-2_V), 82.4 (d, $^1J_{C6-F}$ = 167.9, C-6_{IV}), 78.9 (C-2_{VIII}), 78.8 (C-2_{VI}), 78.6, 78.4, 78.1, 78.0, 77.8, 77.6, 73.3, 73.3, 73.2, 73.2, 72.7, 71.9, 71.8, 71.1, 71.0, 70.1, 69.9, 69.9, 69.8, 69.6, 69.5, 67.0 (C-1'), 66.8, 66.8, 66.8, 66.8, 66.7, 65.5, 65.5, 65.5, 65.4, 65.3, 65.2, 60.9, 60.8, 60.6, 60.5, 50.2 (C-2'); ^{19}F–NMR (565 MHz, D_2O) δ: -203.7 (F_I), -203.8 (F_V), -204.8 (F_{IX}), -204.8 (F_{VII}), -233.7 (F_{IV}); ESI–MS *m/z* calcd. for $C_{56}H_{90}N_3O_{41}F_5$: 1555.5; found: 1578.7 $[M+Na]^+$; ESI–HRMS *m/z* calcd. for $C_{56}H_{90}N_3O_{41}F_5Na$ $[M+Na]^+$: 1578.4862; found: 1578.4833.

3. ^{1}H, ^{13}C and ^{19}F NMR spectra.

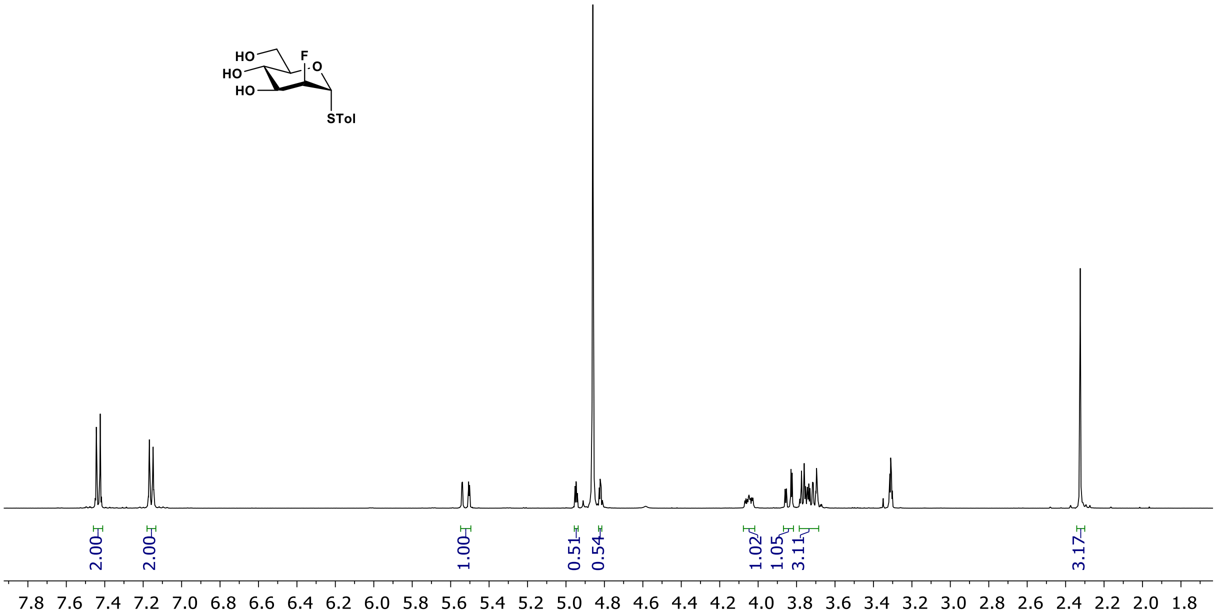

^{1}H-NMR spectrum of **4** (400 MHz, CD_3OD)

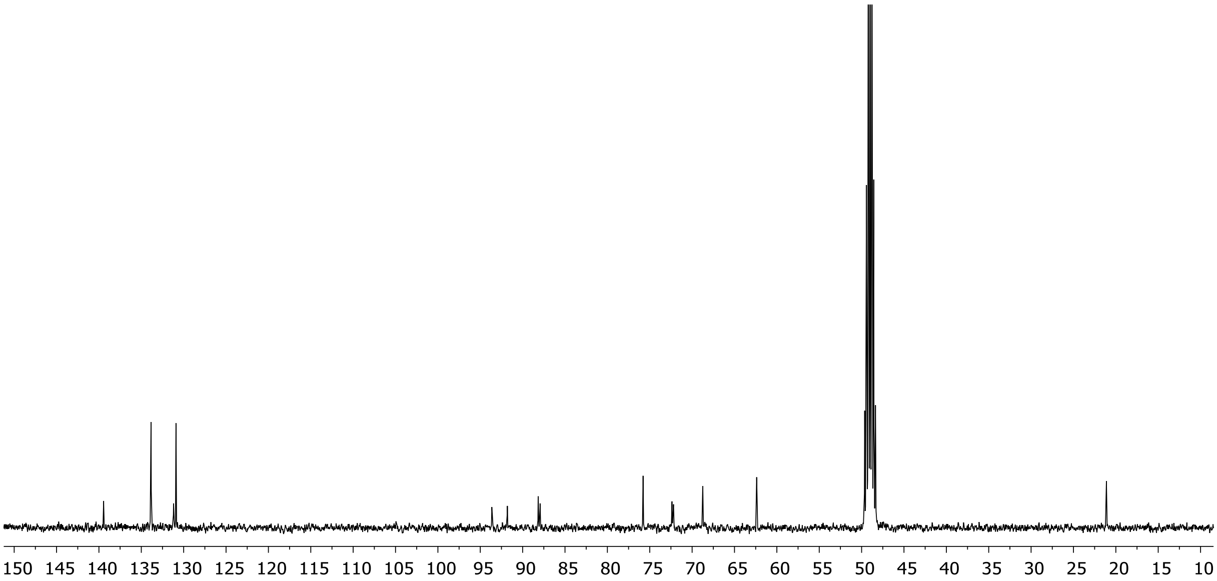

^{13}C-NMR spectrum of **4** (101 MHz, CD_3OD)

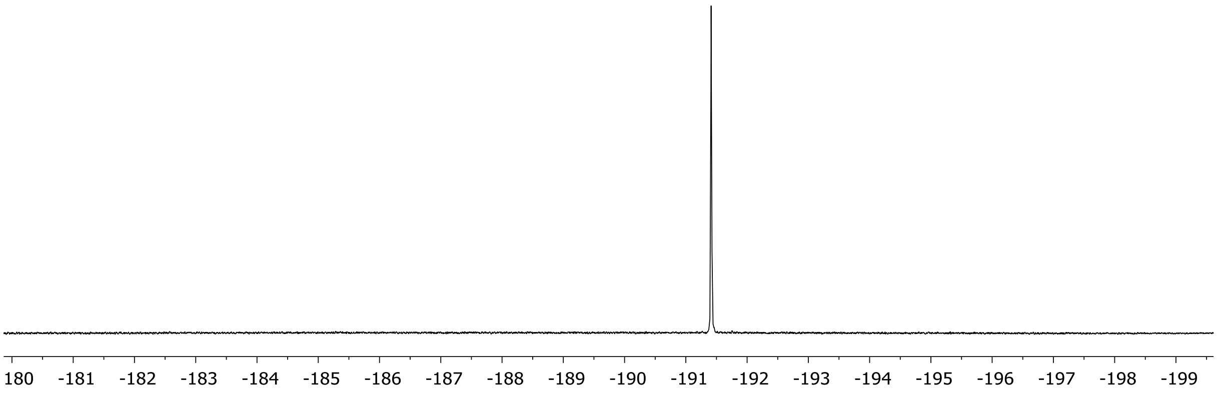

^{19}F-NMR spectrum of **4** (376 MHz, CD_3OD)

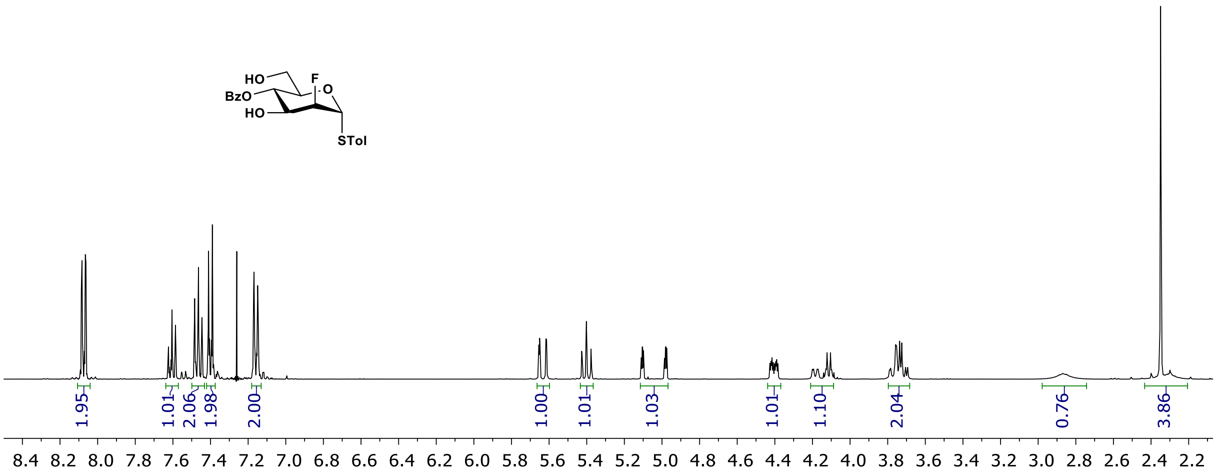

^{1}H-NMR spectrum of **5** (400 MHz, $CDCl_3$)

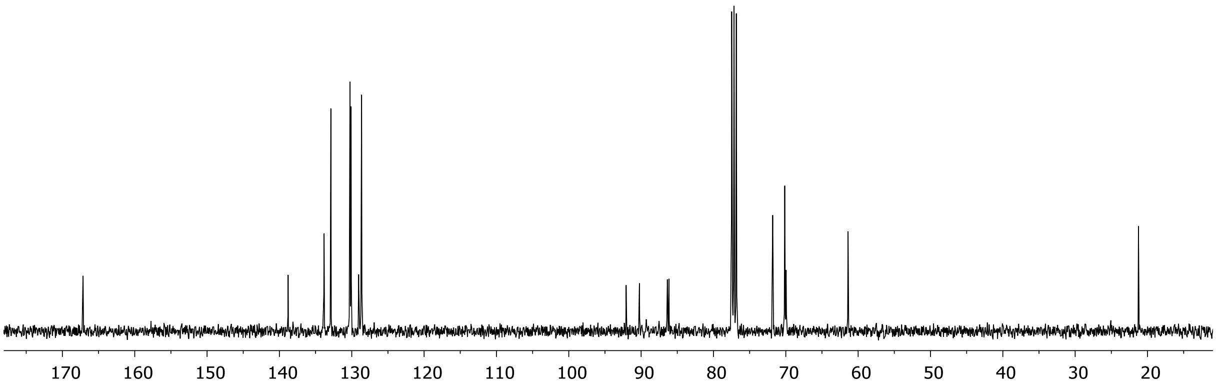

^{13}C-NMR spectrum of **5** (101 MHz, $CDCl_3$)

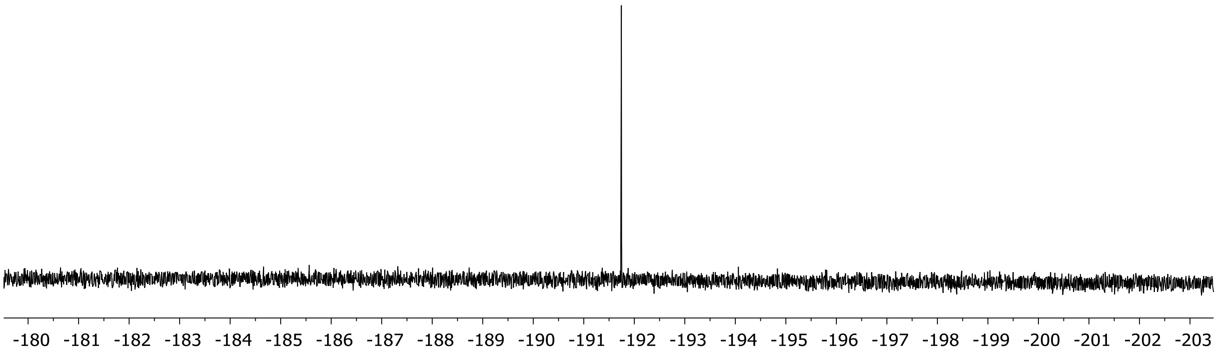

^{19}F-NMR spectrum of **5** (376 MHz, $CDCl_3$)

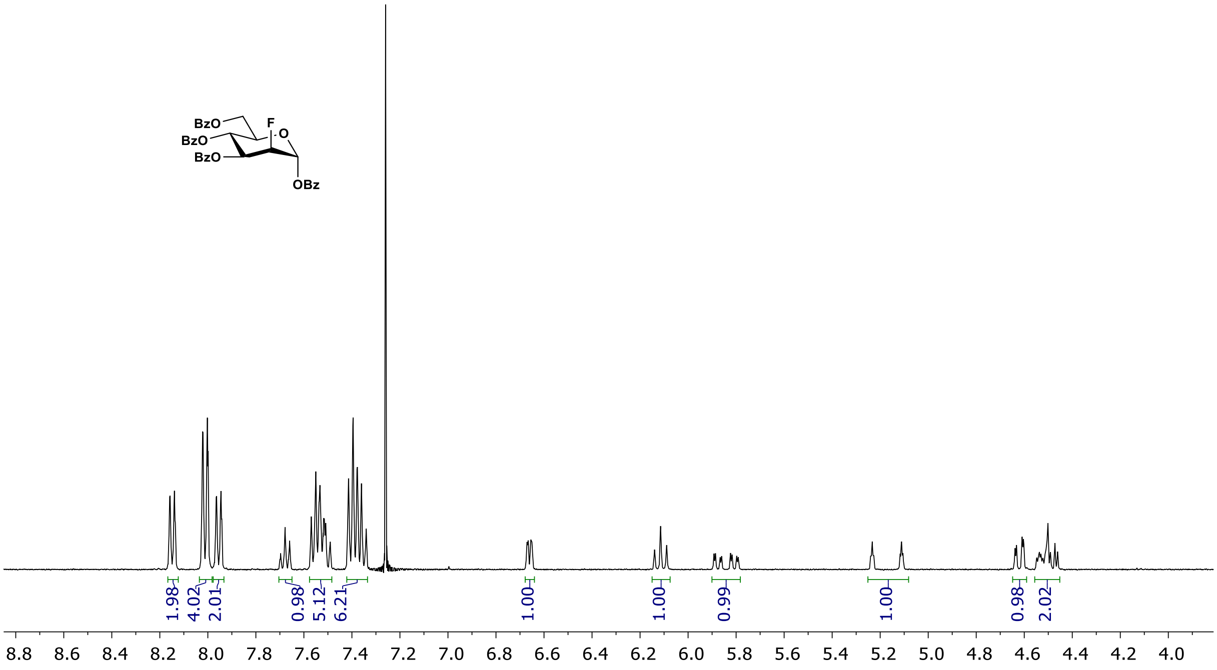

^{1}H-NMR spectrum of **6** (400 MHz, $CDCl_3$)

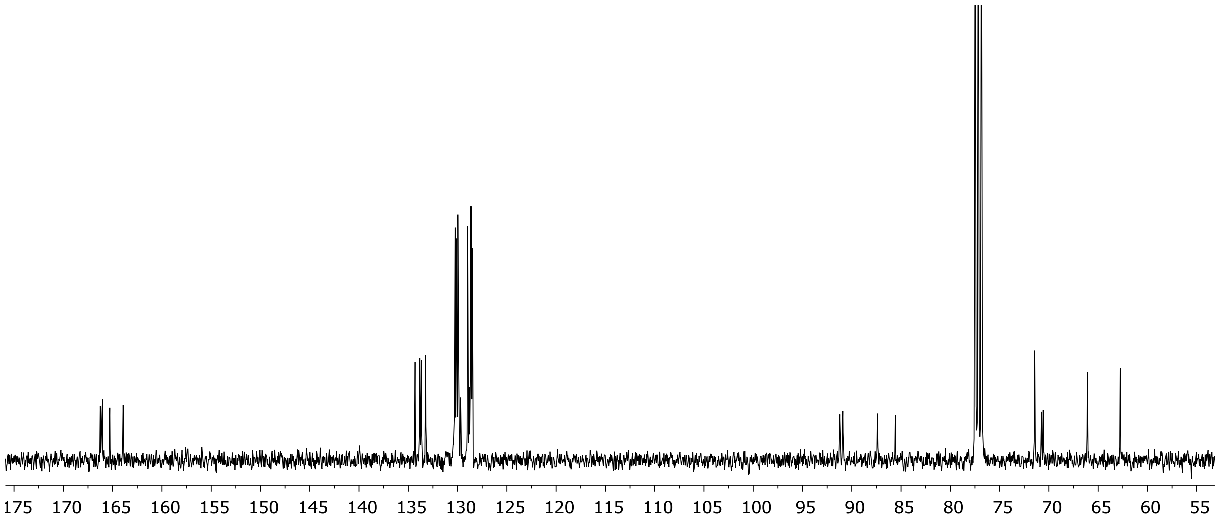

^{13}C-NMR spectrum of **6** (101 MHz, $CDCl_3$)

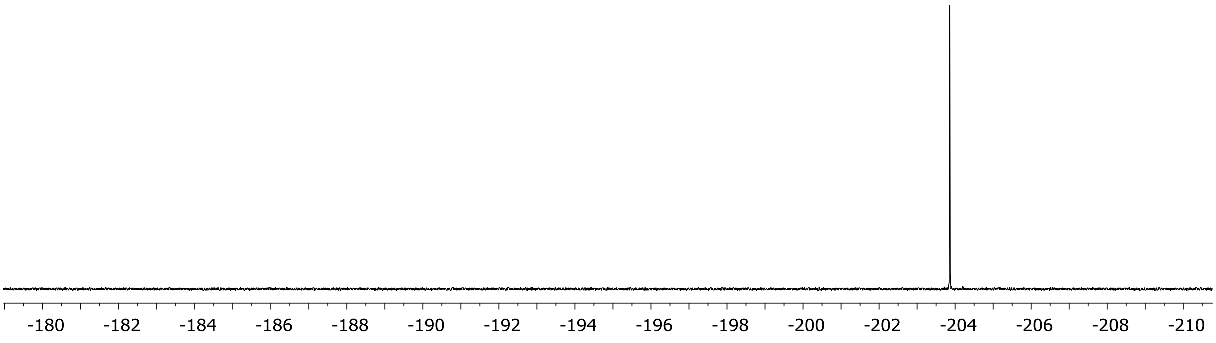

^{19}F-NMR spectrum of **6** (376 MHz, $CDCl_3$)

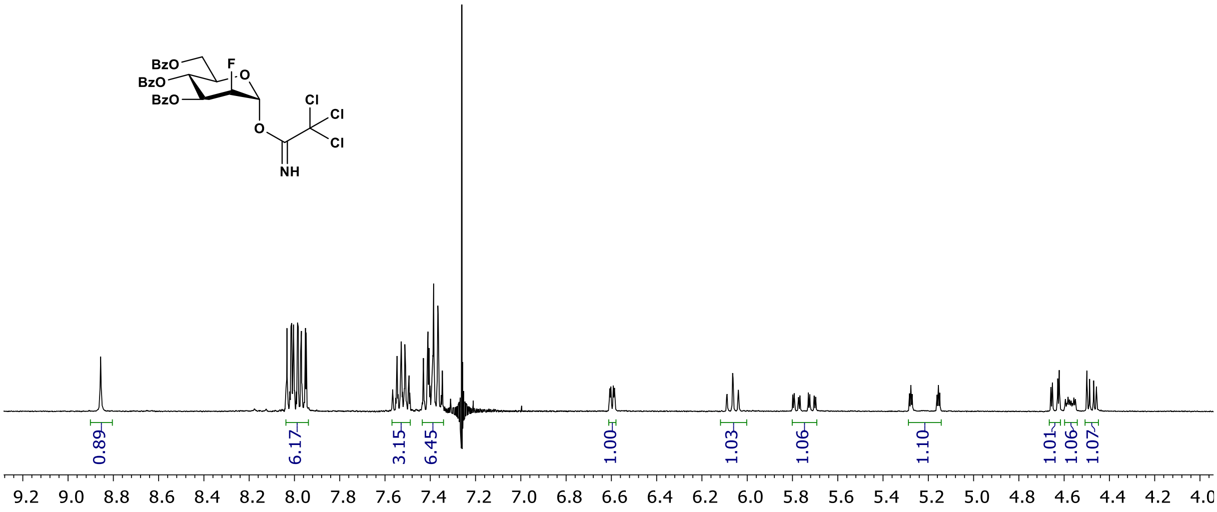

^{1}H-NMR spectrum of **7** (400 MHz, $CDCl_3$)

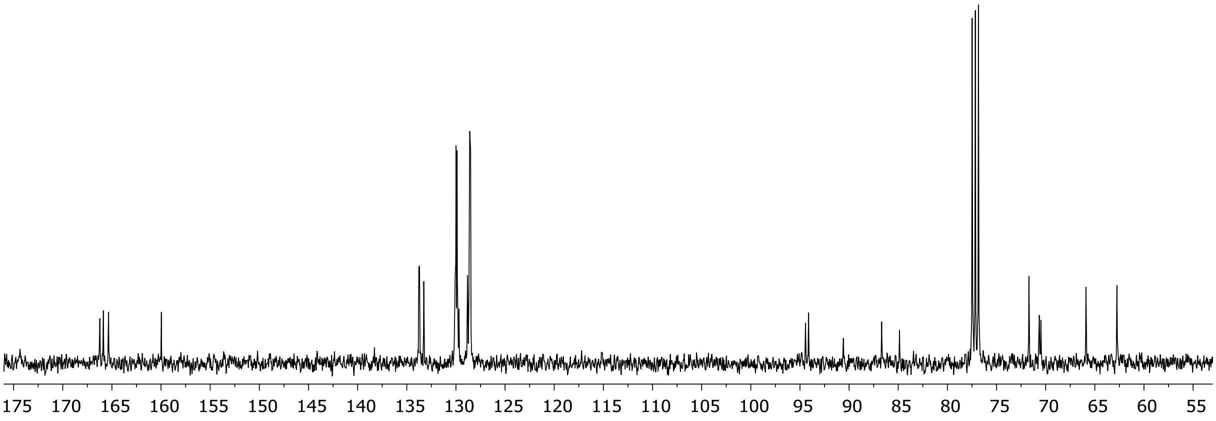

^{13}C-NMR spectrum of **7** (101 MHz, $CDCl_3$)

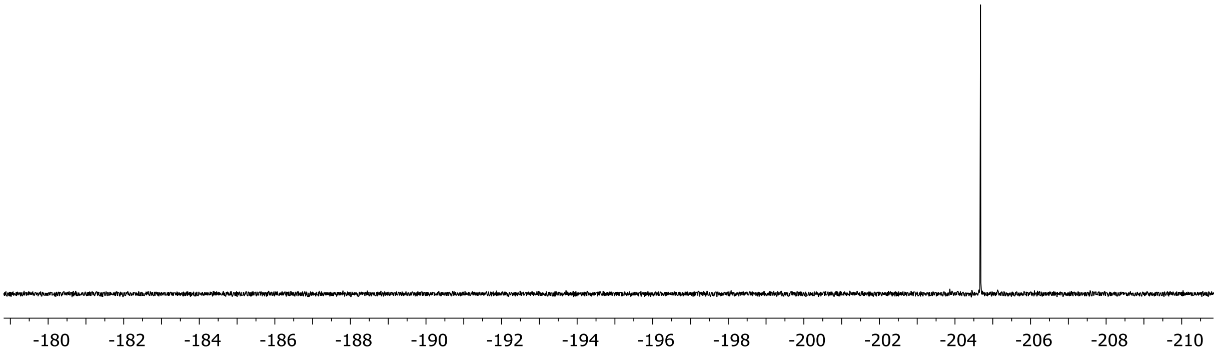

^{19}F-NMR spectrum of **7** (376 MHz, $CDCl_3$)

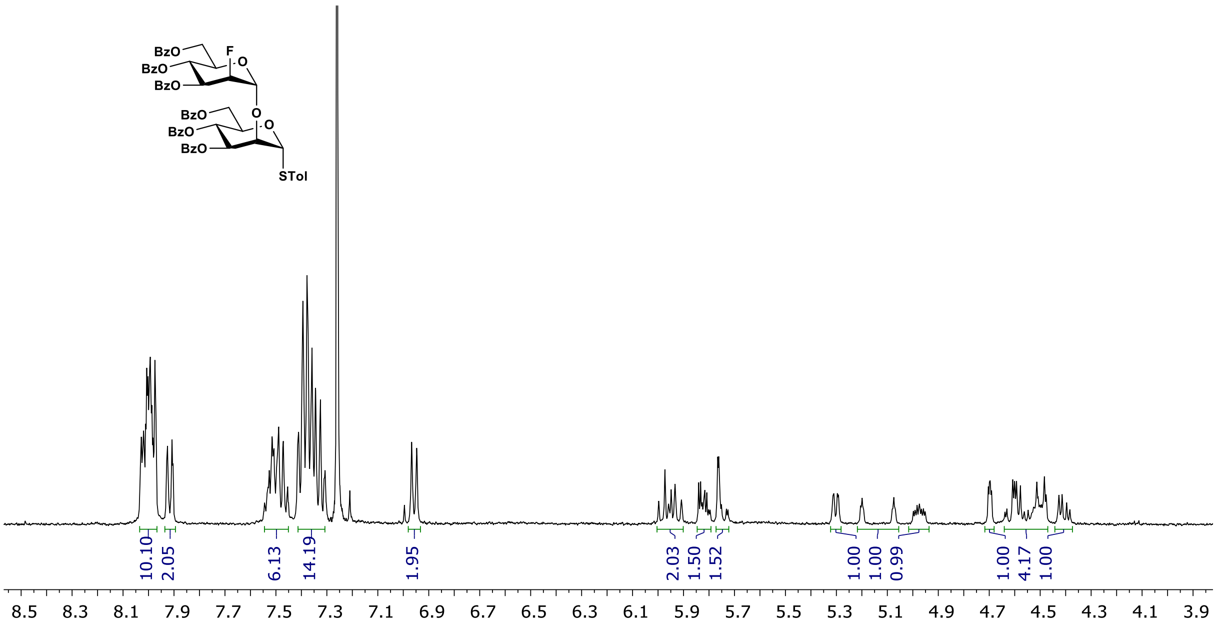

^{1}H-NMR spectrum of **9** (400 MHz, $CDCl_3$)

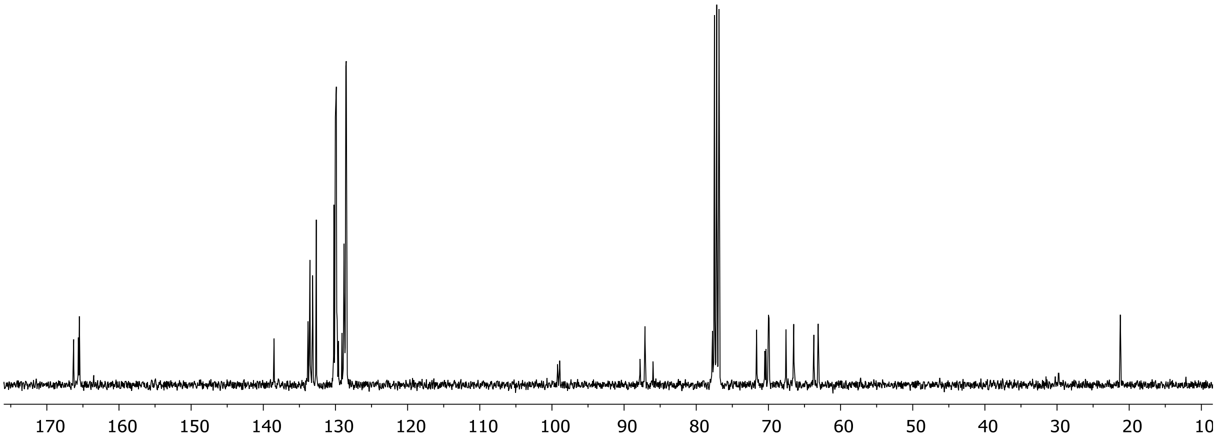

^{13}C-NMR spectrum of **9** (101 MHz, $CDCl_3$)

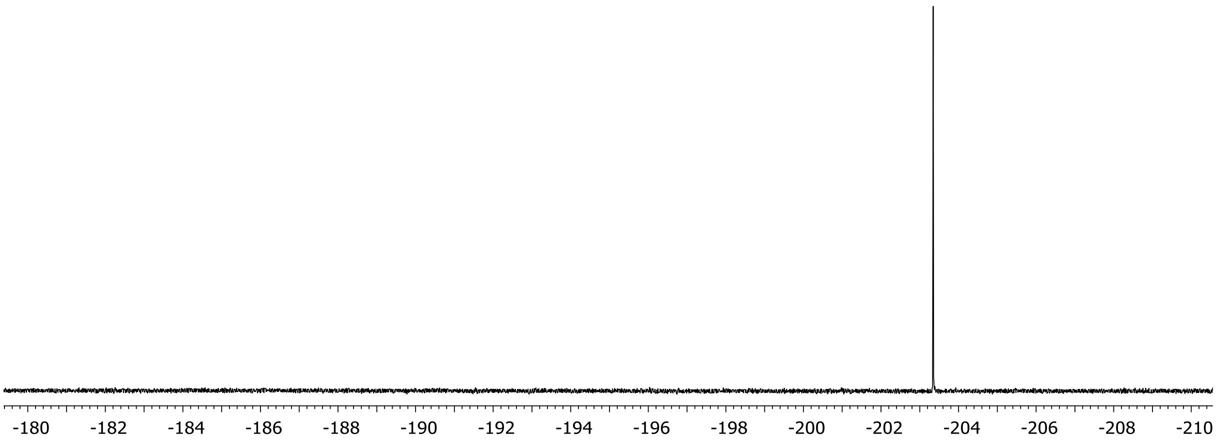

^{19}F-NMR spectrum of **9** (376 MHz, $CDCl_3$)

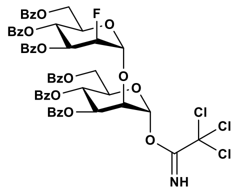

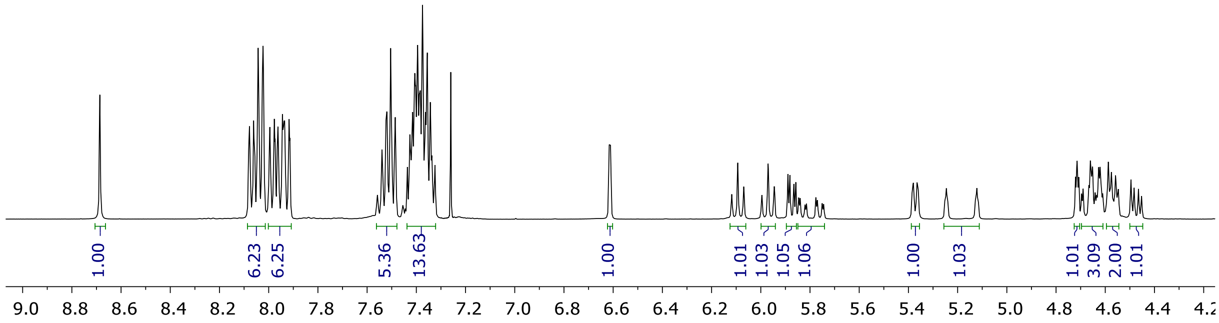

^{1}H-NMR spectrum of **10** (400 MHz, $CDCl_3$)

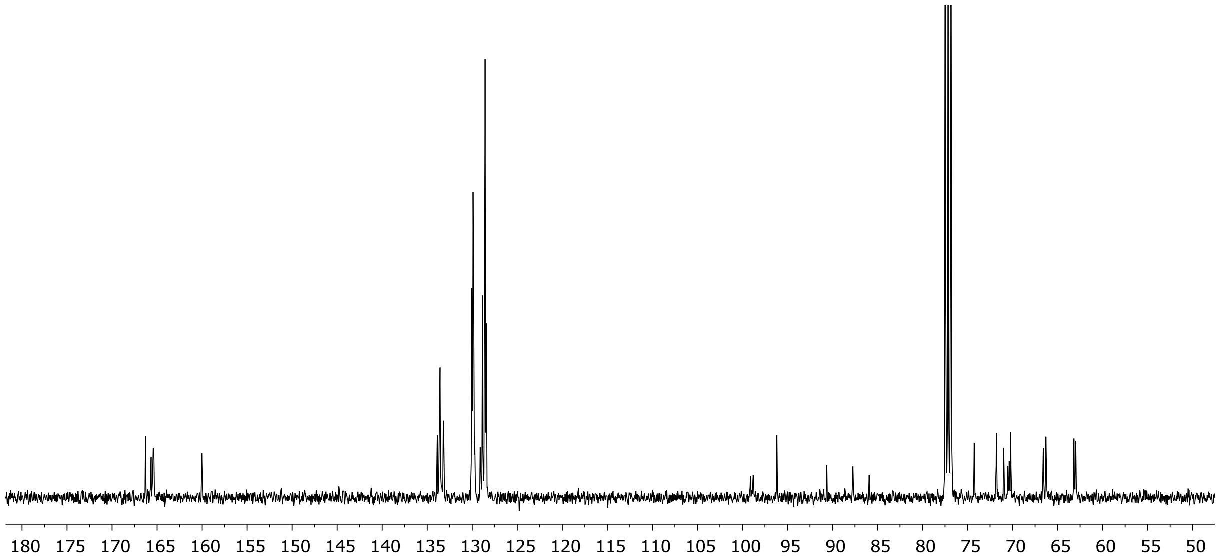

^{13}C-NMR spectrum of **10** (101 MHz, $CDCl_3$)

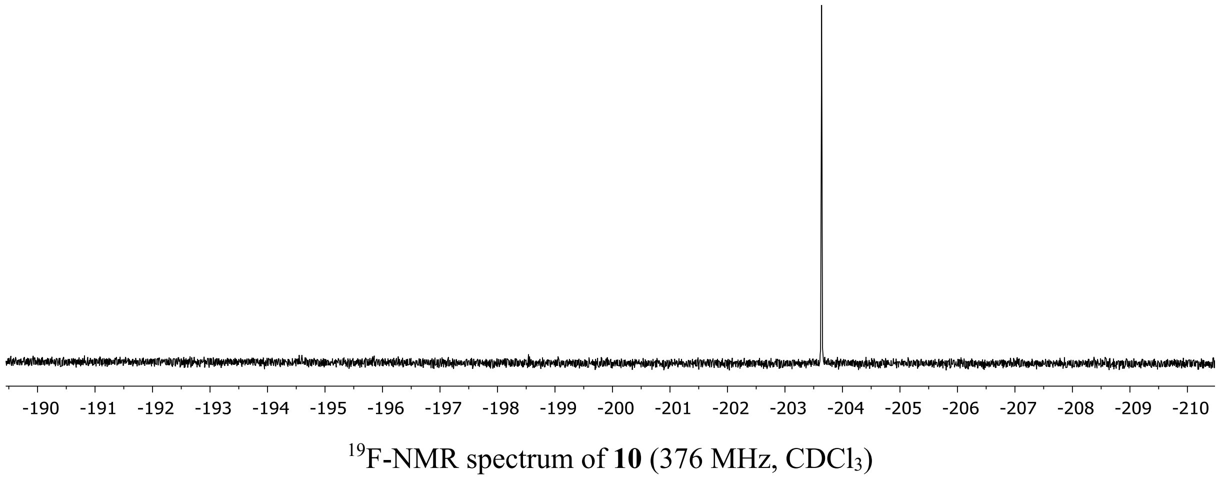

^{19}F-NMR spectrum of **10** (376 MHz, $CDCl_3$)

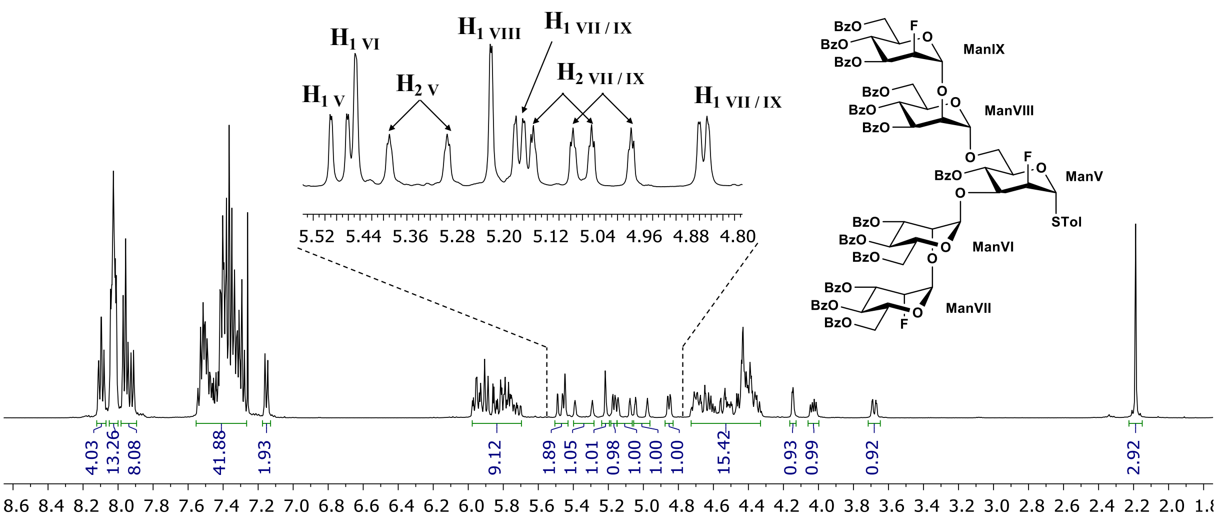

^{1}H-NMR spectrum of **11** (500 MHz, $CDCl_3$)

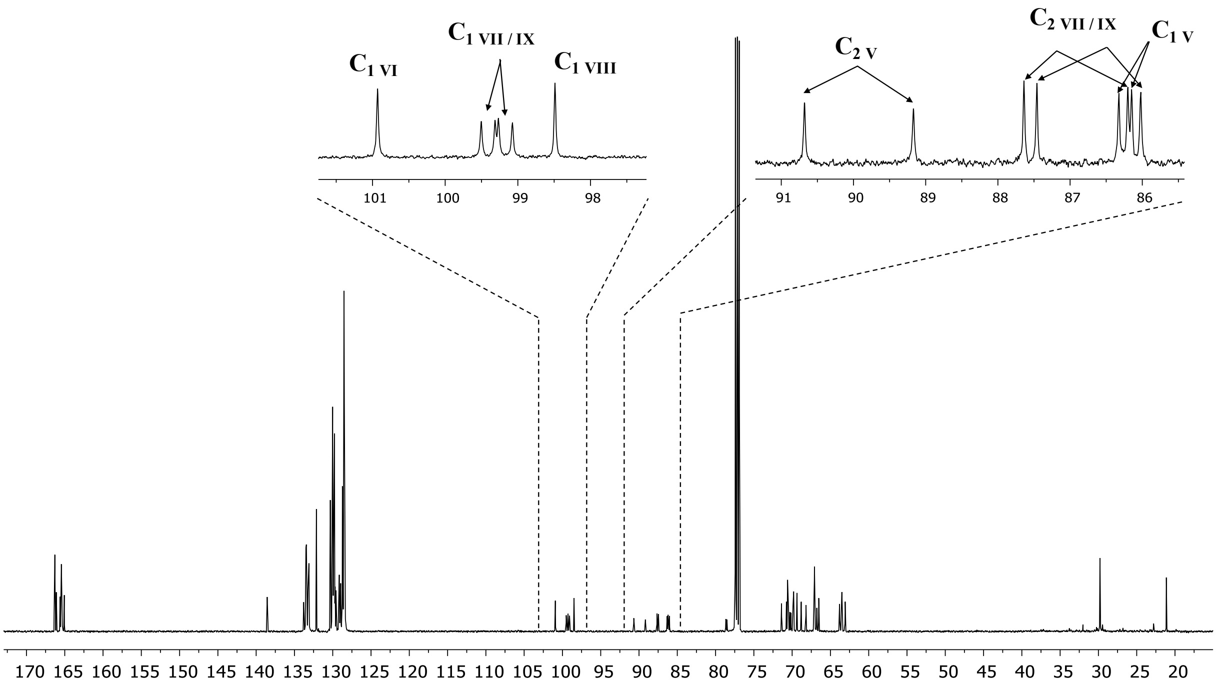

^{13}C-NMR spectrum of **11** (126 MHz, $CDCl_3$)

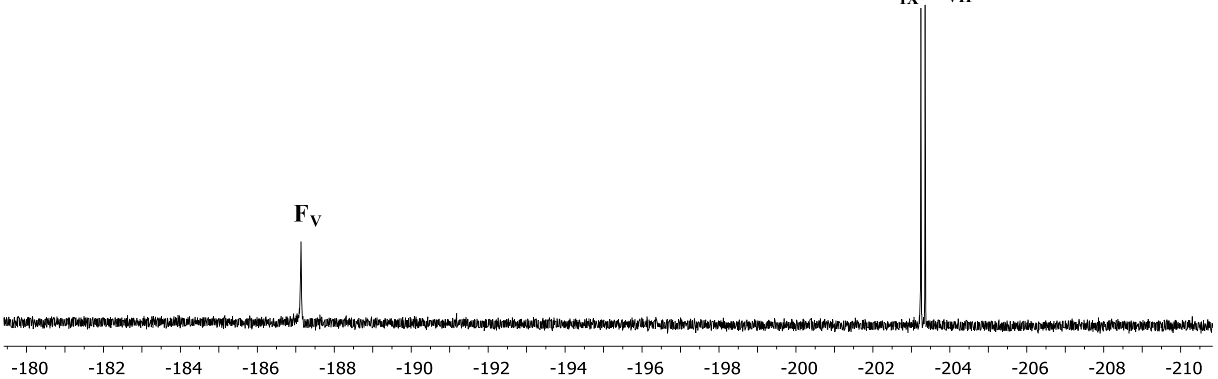

^{19}F-NMR spectrum of **11** (376 MHz, $CDCl_3$)

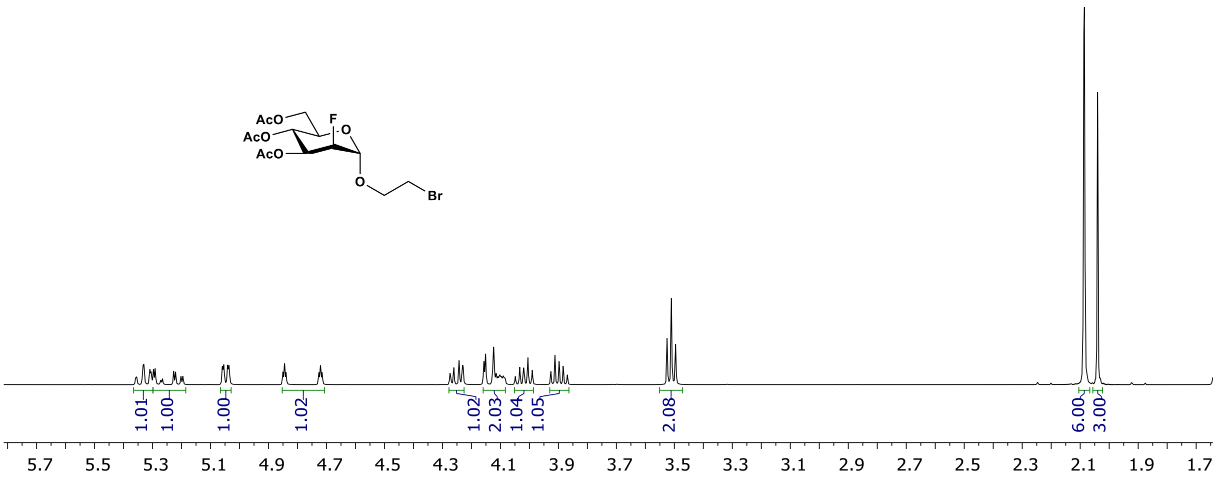

^{1}H-NMR spectrum of **22** (400 MHz, $CDCl_3$)

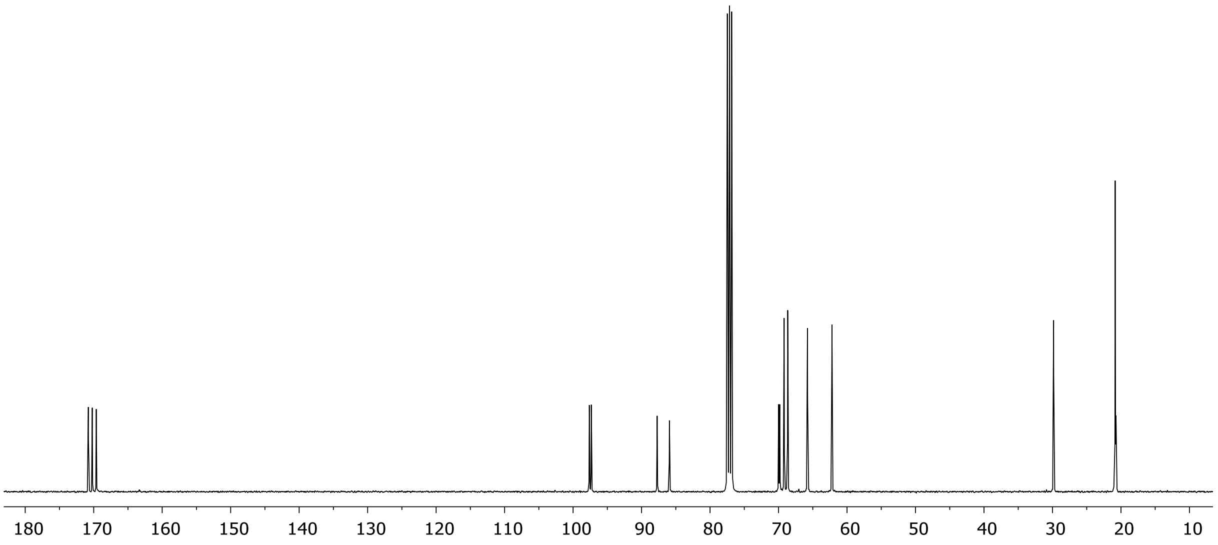

^{13}C-NMR spectrum of **22** (101 MHz, $CDCl_3$)

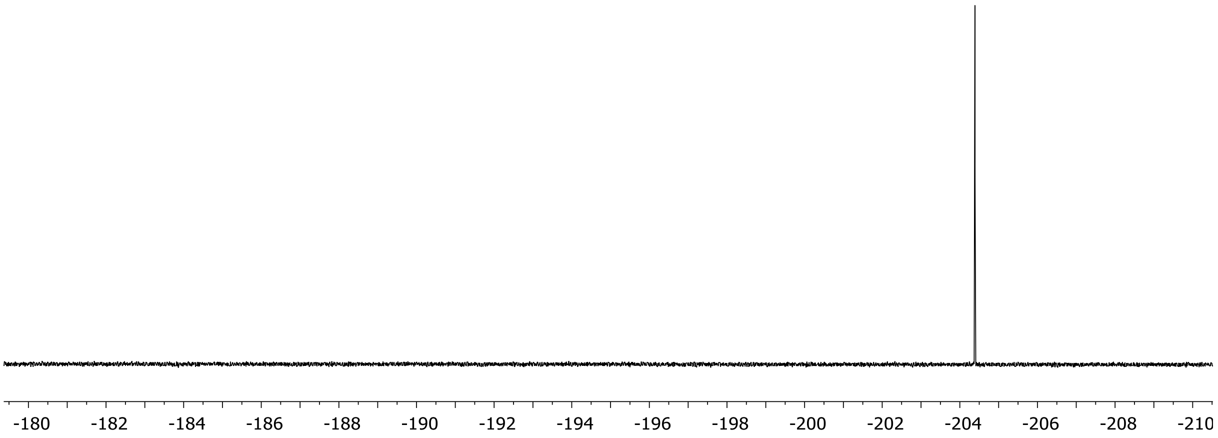

^{19}F-NMR spectrum of **22** (376 MHz, $CDCl_3$)

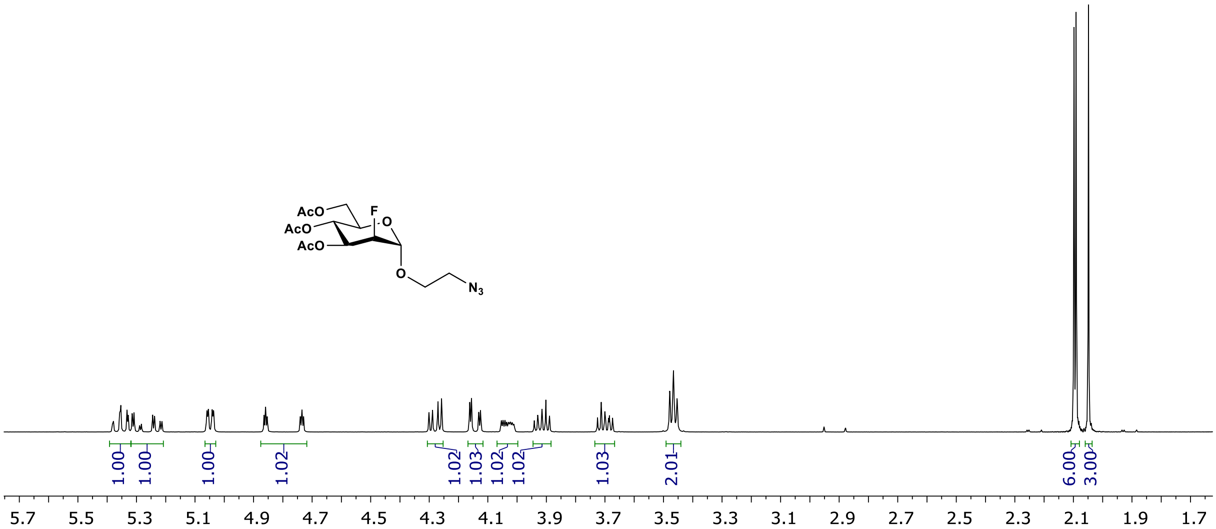

^{1}H-NMR spectrum of **13** (400 MHz, $CDCl_3$)

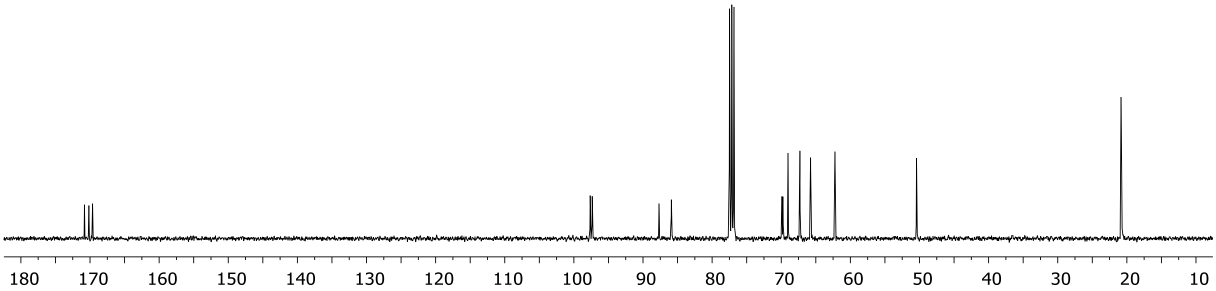

^{13}C-NMR spectrum of **13** (101 MHz, $CDCl_3$)

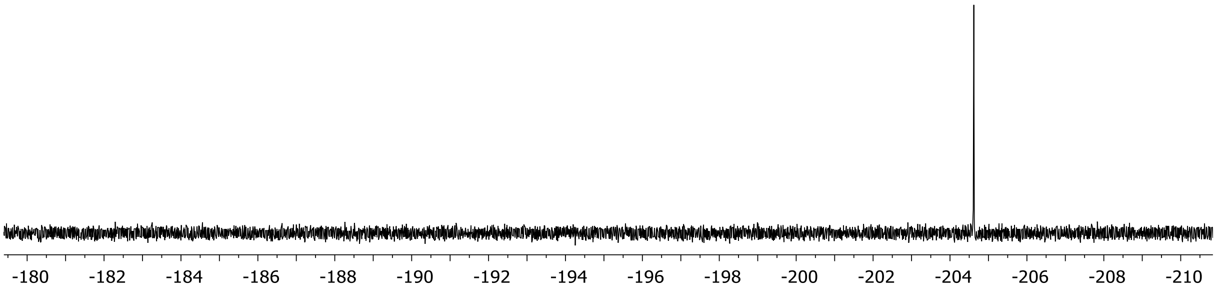

^{19}F-NMR spectrum of **13** (376 MHz, $CDCl_3$)

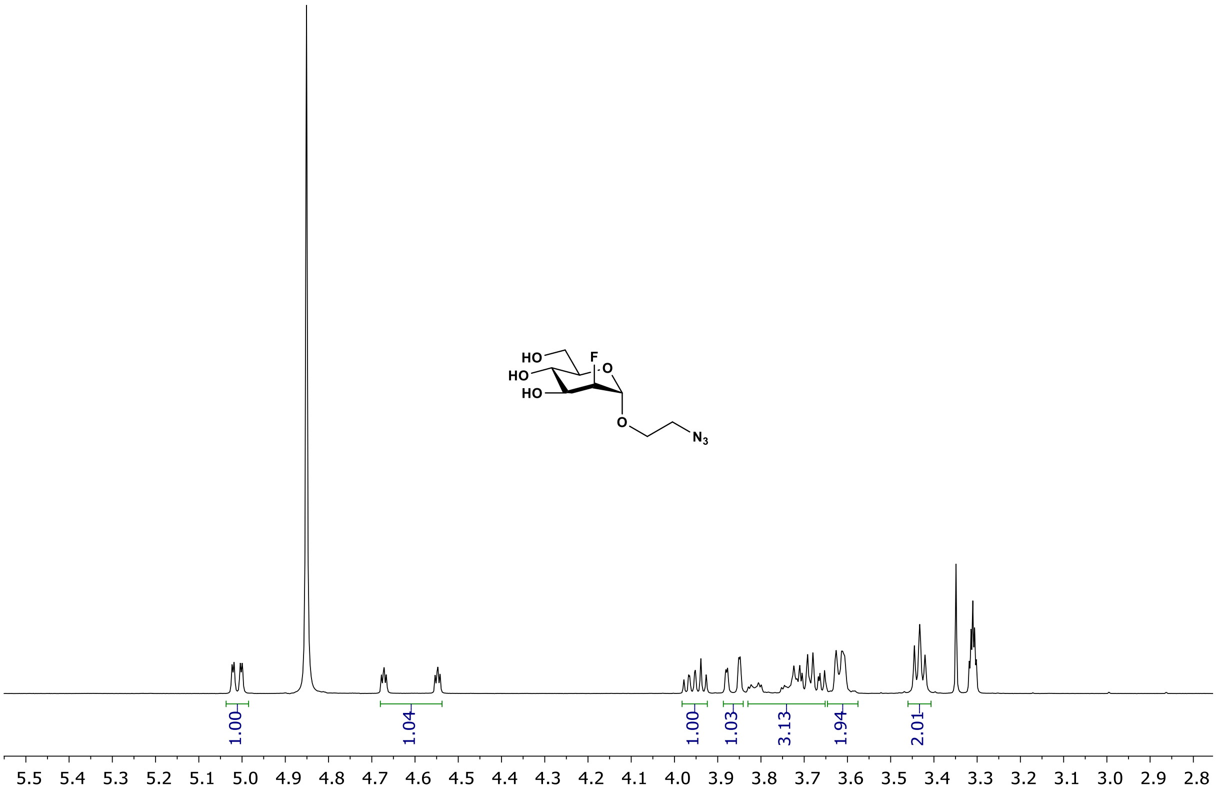

^{1}H-NMR spectrum of **23** (400 MHz, CD_3OD)

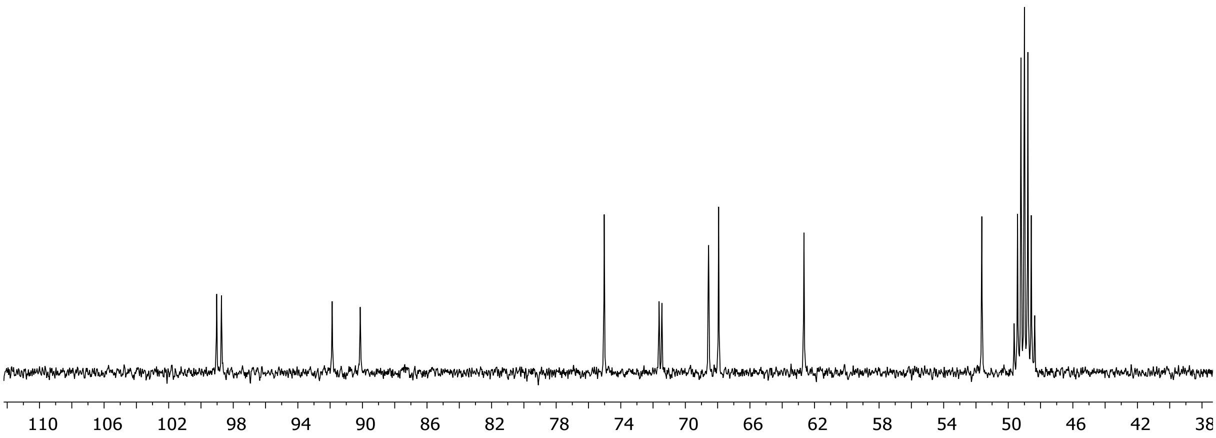

^{13}C-NMR spectrum of **23** (101 MHz, CD_3OD)

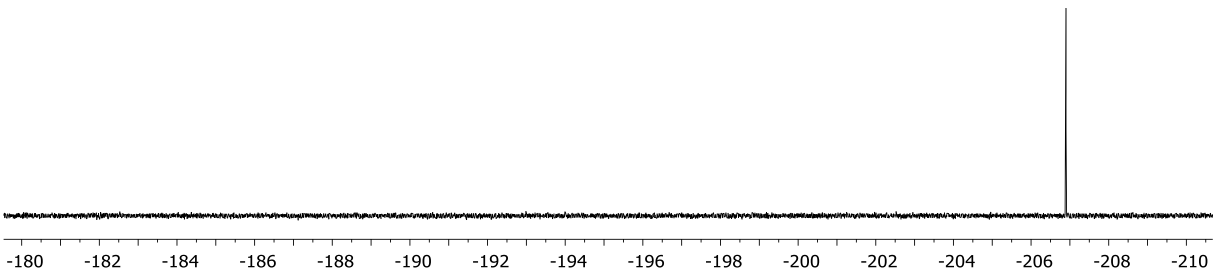

^{19}F-NMR spectrum of **23** (376 MHz, CD_3OD)

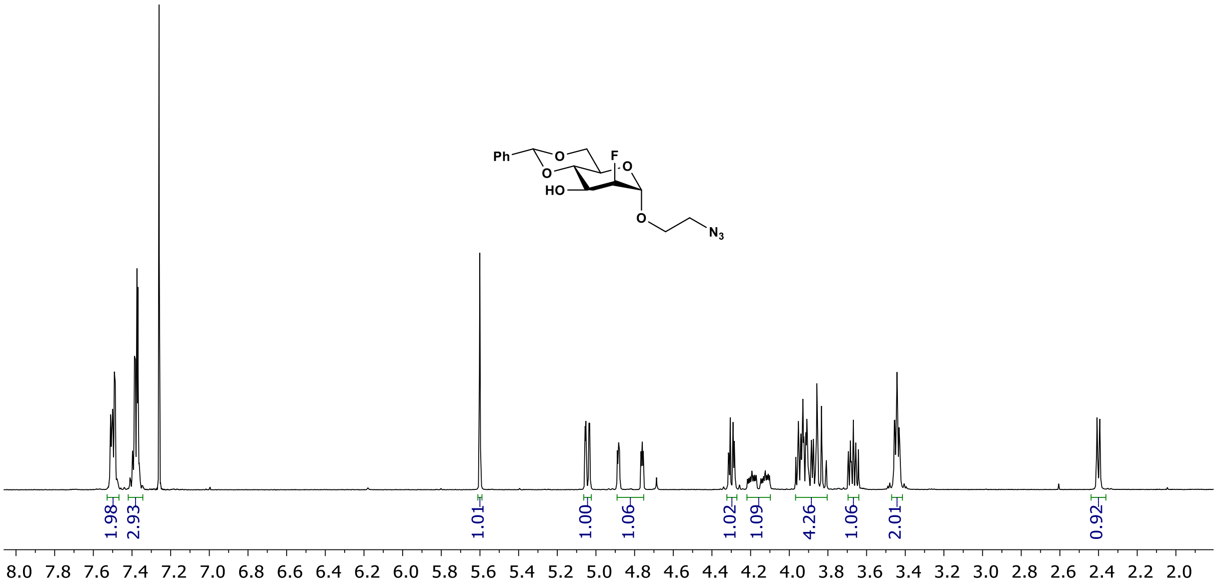

^{1}H-NMR spectrum of **14** (400 MHz, $CDCl_3$)

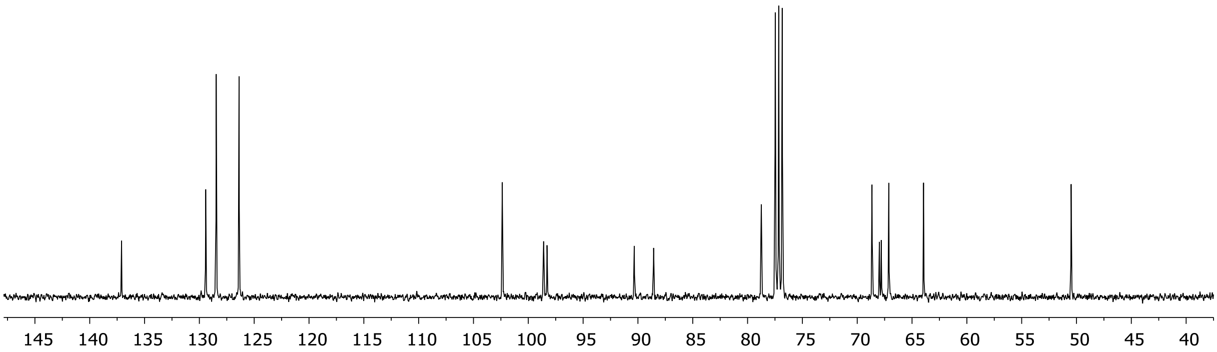

^{13}C-NMR spectrum of **14** (101 MHz, $CDCl_3$)

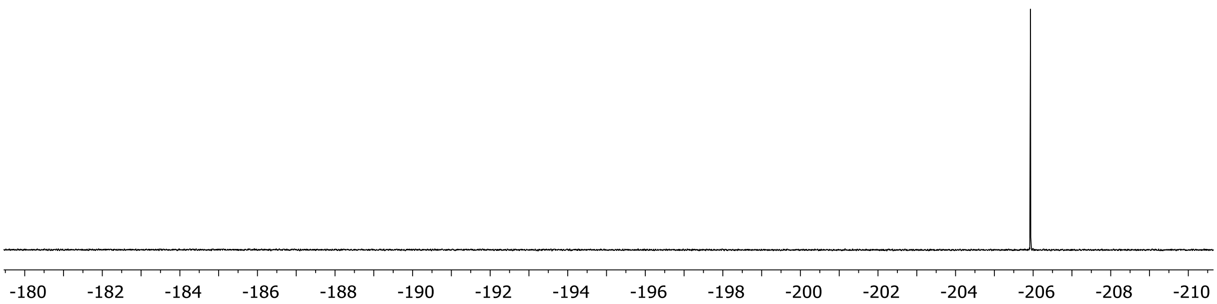

^{19}F-NMR spectrum of **14** (376 MHz, $CDCl_3$)

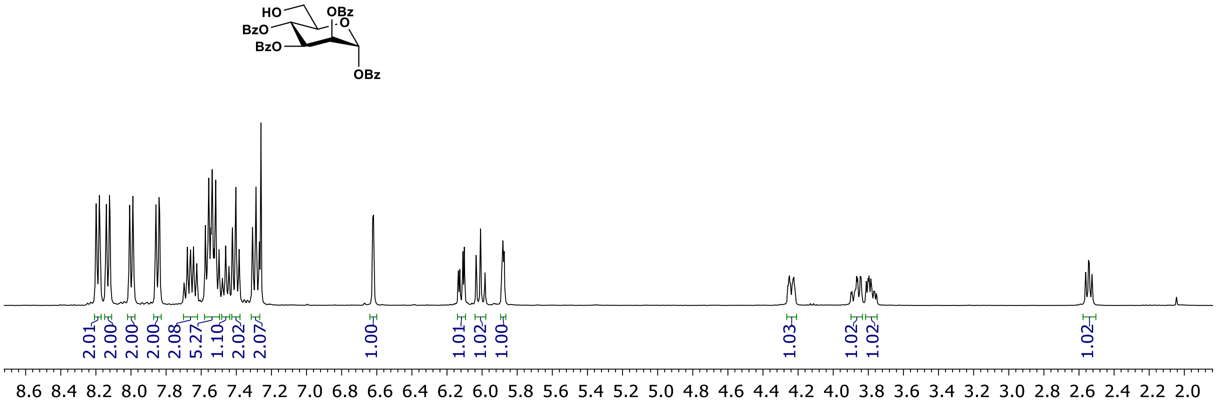

^{1}H-NMR spectrum of **24** (400 MHz, $CDCl_3$)

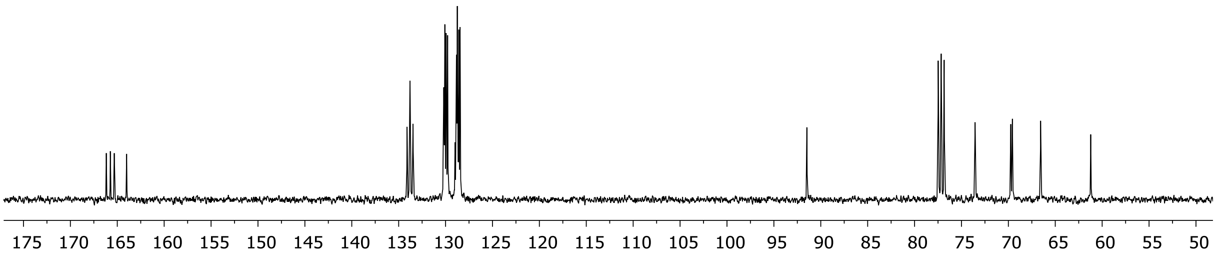

^{13}C-NMR spectrum of **24** (101 MHz, $CDCl_3$)

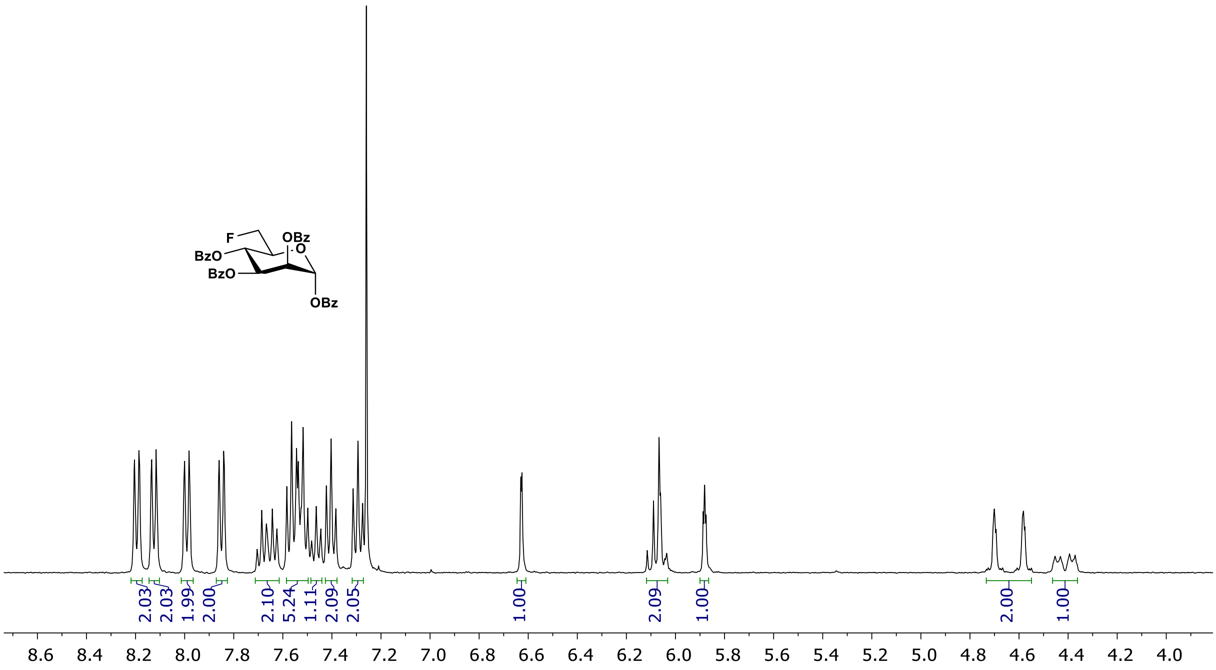

^{1}H-NMR spectrum of **15** (400 MHz, $CDCl_3$)

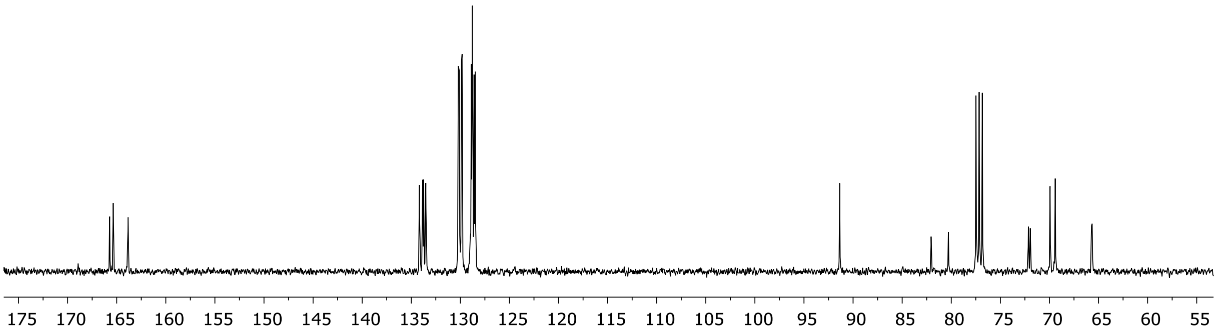

^{13}C-NMR spectrum of **15** (101 MHz, $CDCl_3$)

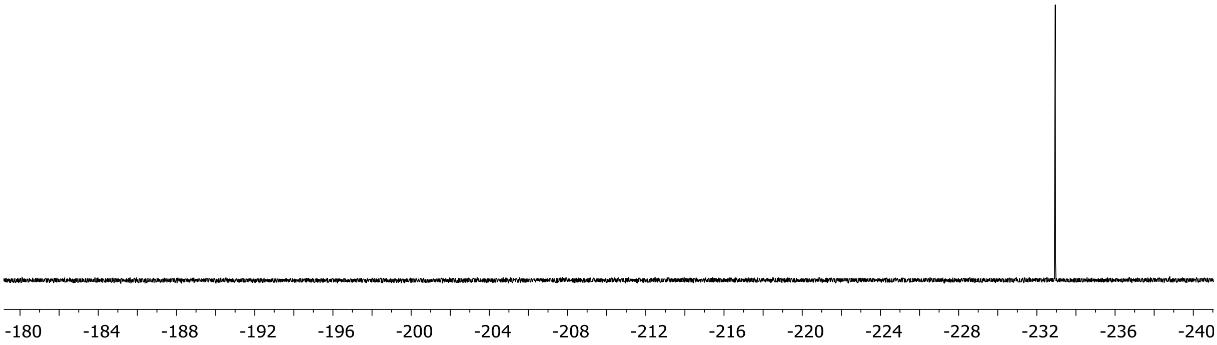

^{19}F-NMR spectrum of **15** (376 MHz, $CDCl_3$)

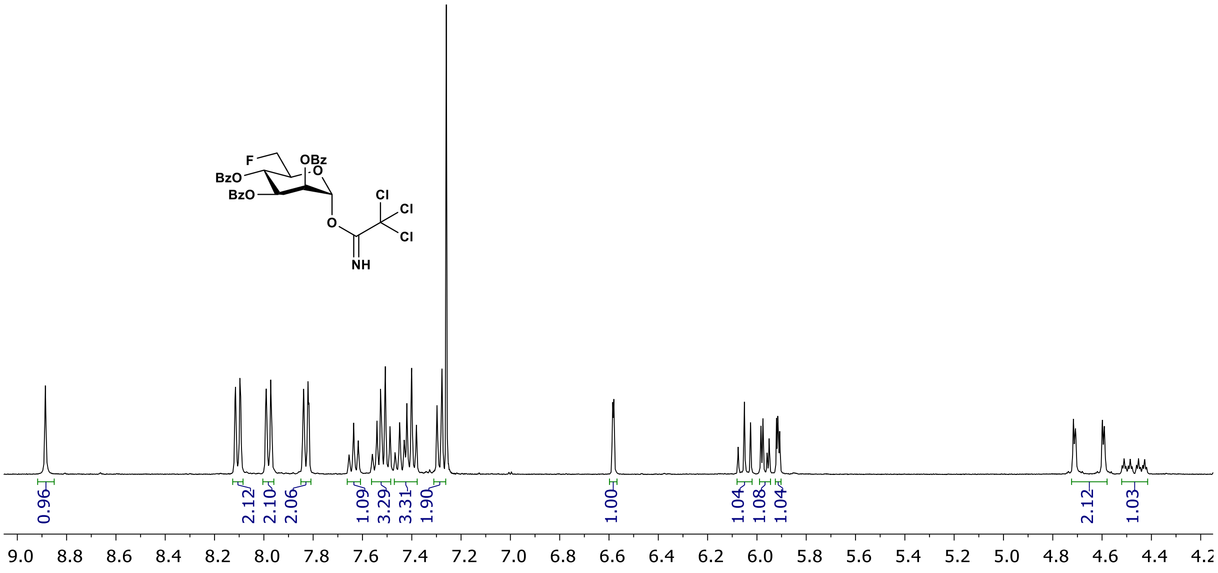

^{1}H-NMR spectrum of **16** (400 MHz, $CDCl_3$)

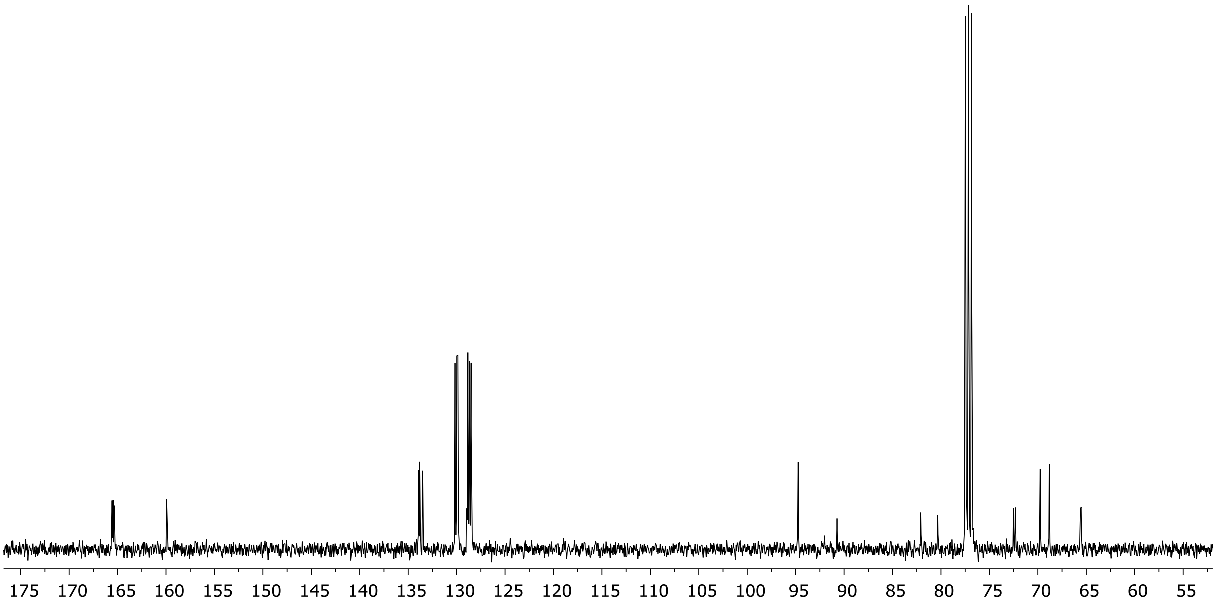

^{13}C-NMR spectrum of **16** (101 MHz, $CDCl_3$)

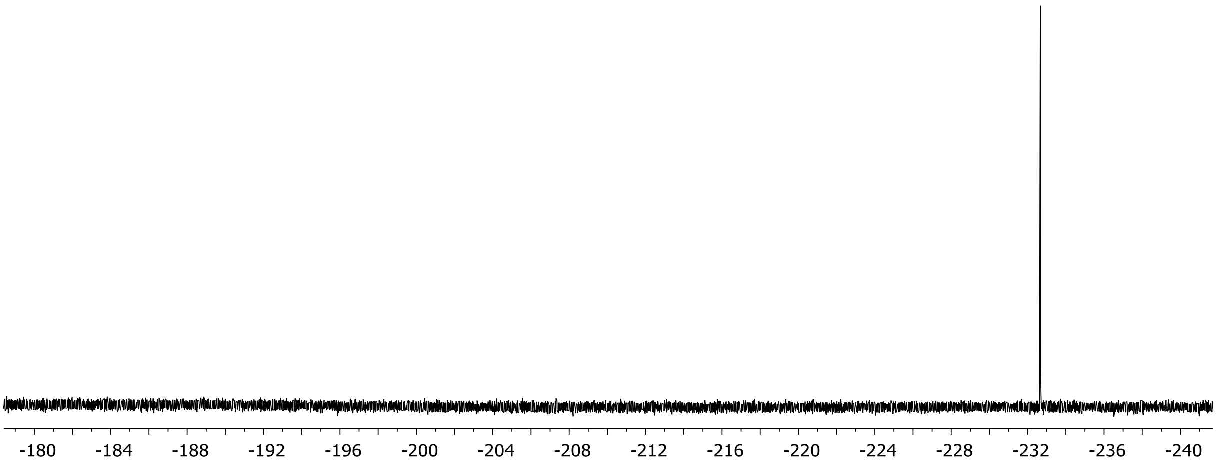

^{19}F-NMR spectrum of **16** (376 MHz, $CDCl_3$)

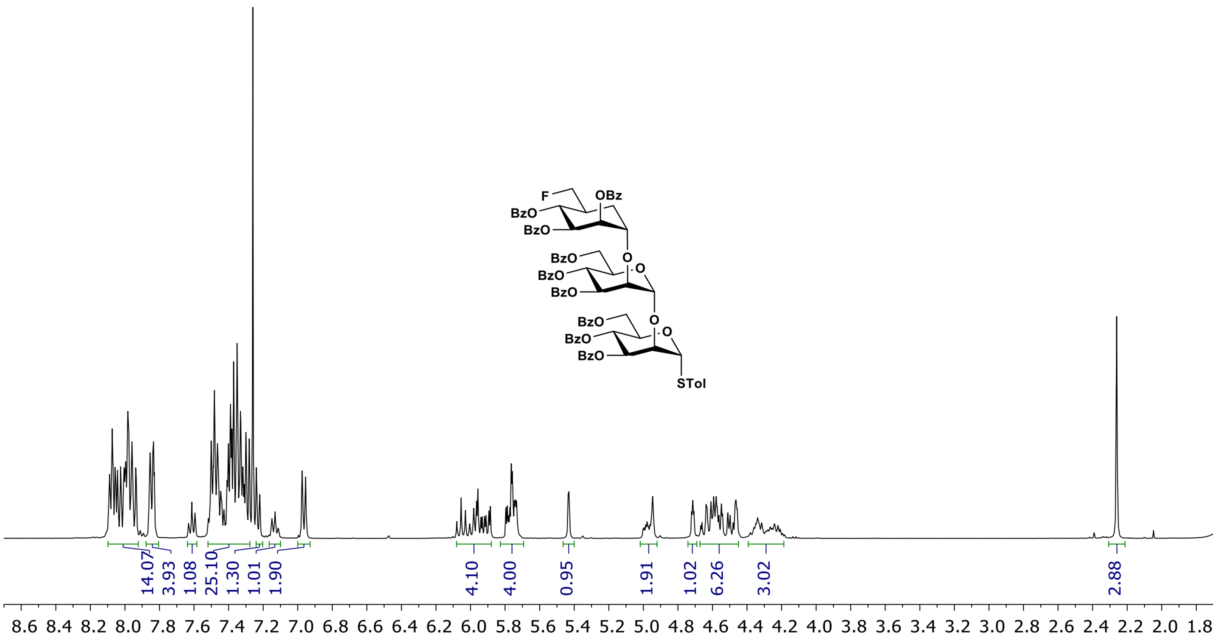

^{1}H-NMR spectrum of **18** (400 MHz, $CDCl_3$)

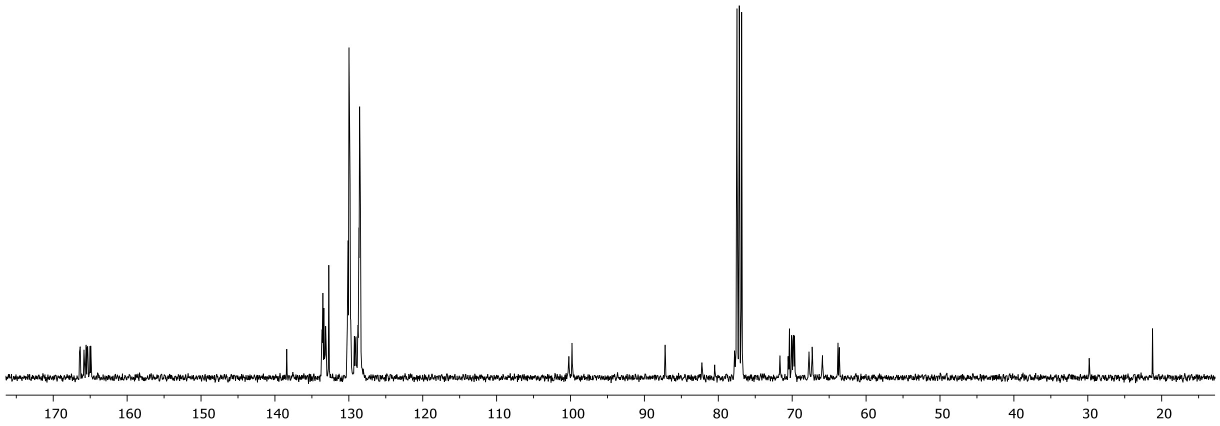

^{13}C-NMR spectrum of **18** (101 MHz, $CDCl_3$)

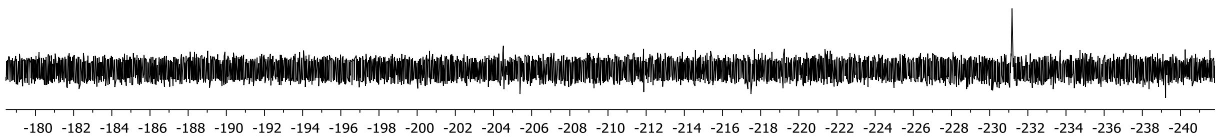

^{19}F-NMR spectrum of **18** (376 MHz, $CDCl_3$)

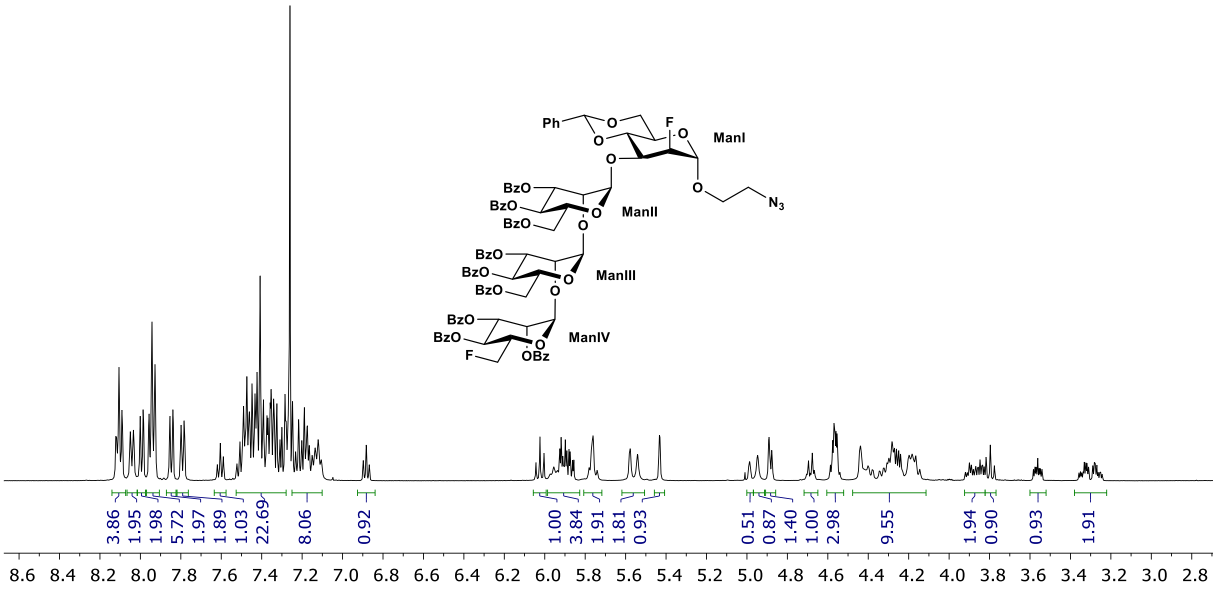

^{1}H-NMR spectrum of **19** (400 MHz, $CDCl_3$)

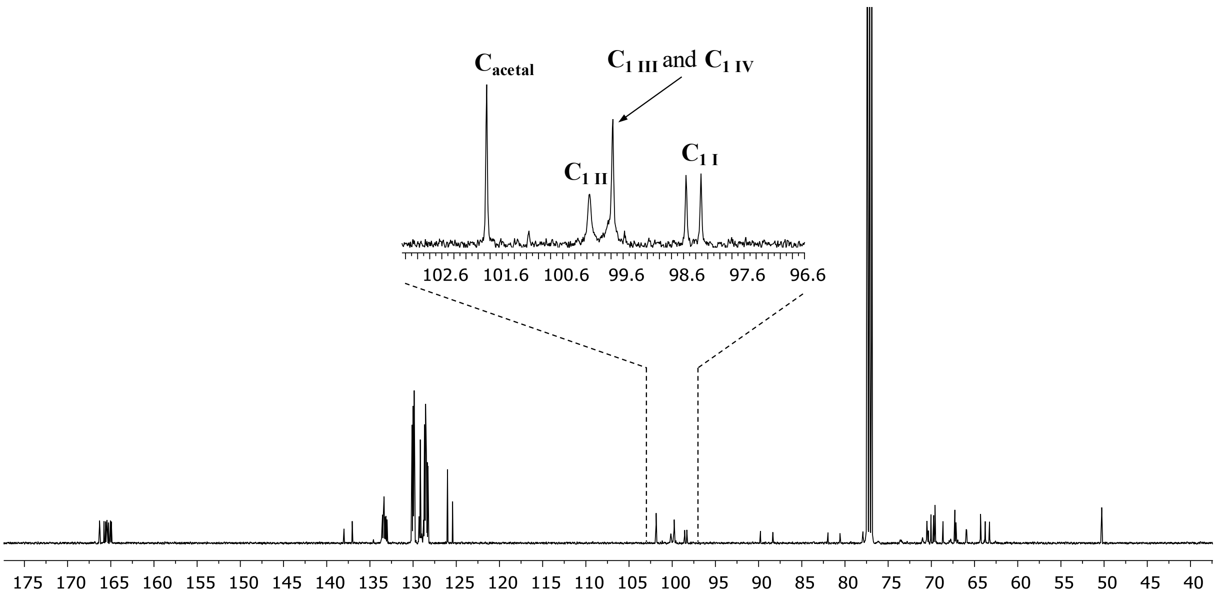

^{13}C-NMR spectrum of **19** (101 MHz, $CDCl_3$)

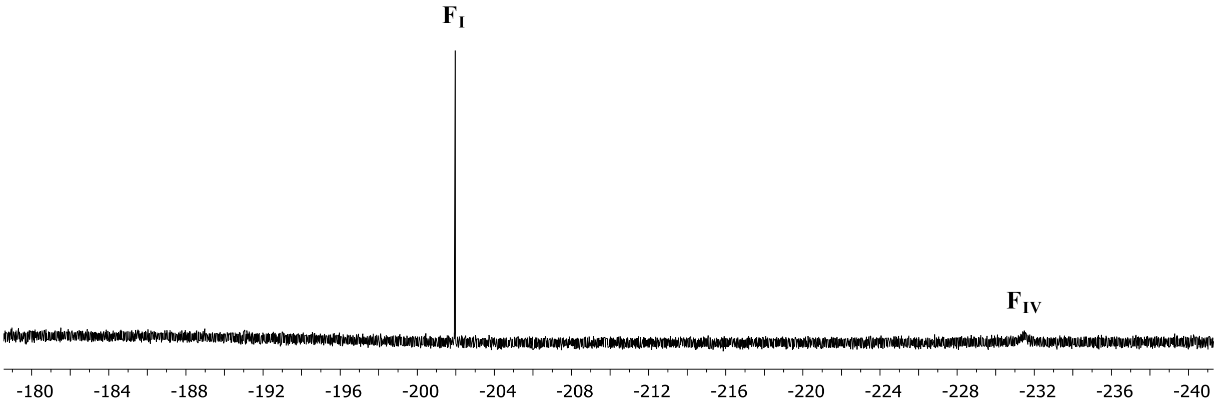

^{19}F-NMR spectrum of **19** (376 MHz, $CDCl_3$)

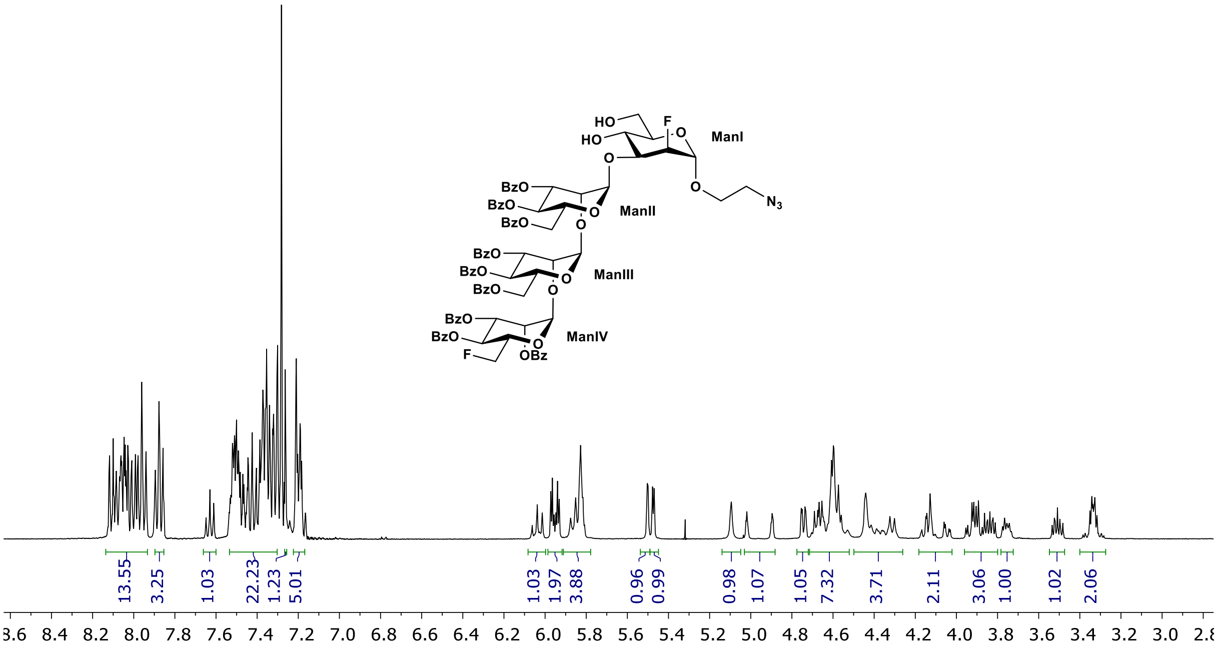

^{1}H-NMR spectrum of **20** (400 MHz, $CDCl_3$)

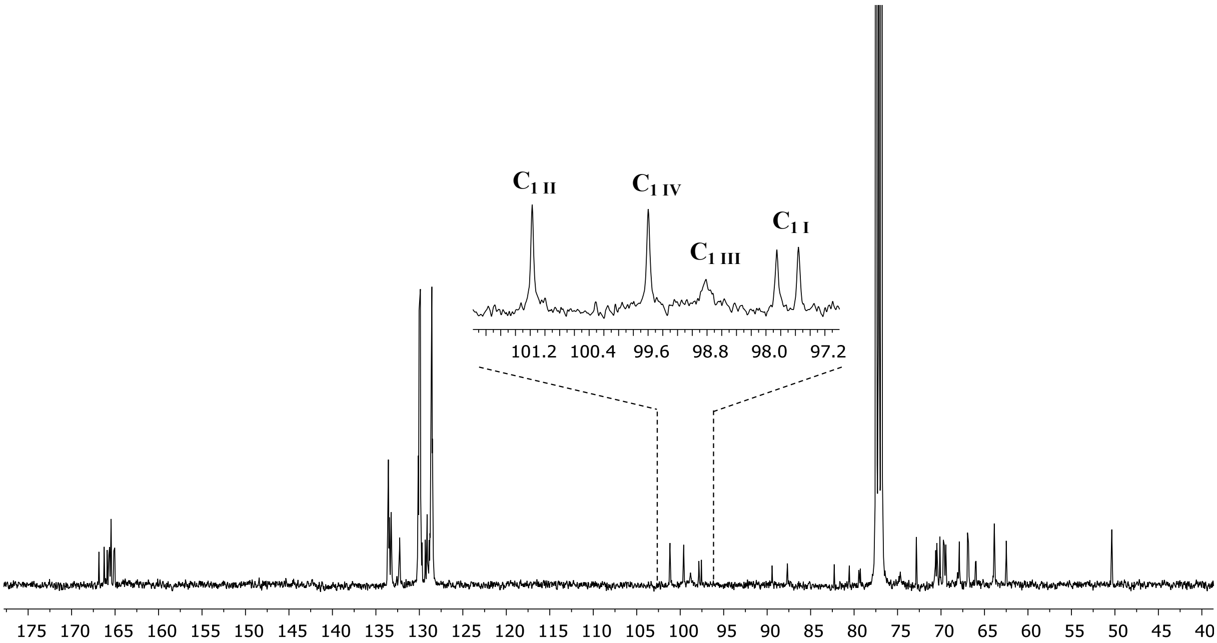

^{13}C-NMR spectrum of **20** (101 MHz, $CDCl_3$)

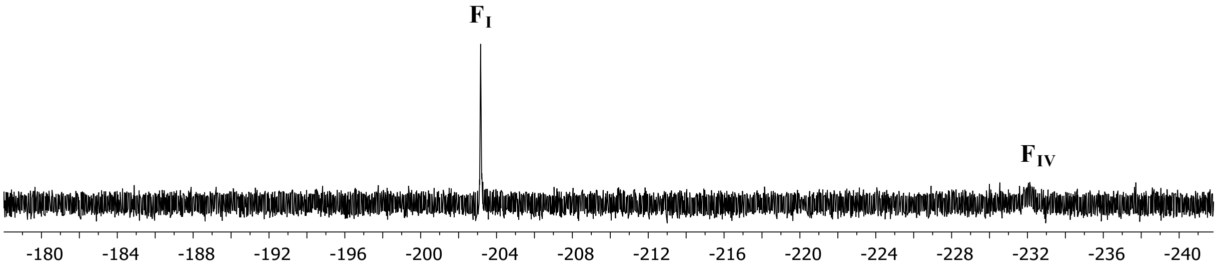

^{19}F-NMR spectrum of **20** (376 MHz, $CDCl_3$)

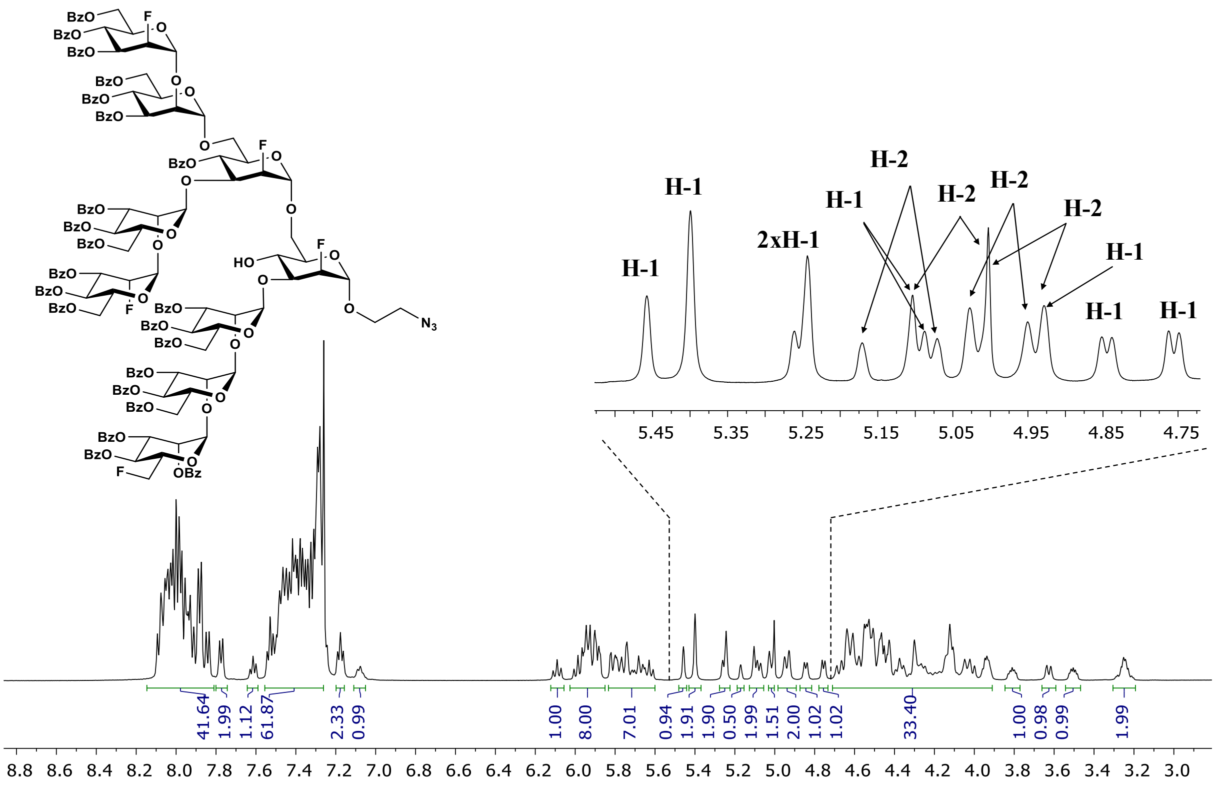

^{1}H-NMR spectrum of **21** (500 MHz, $CDCl_3$)

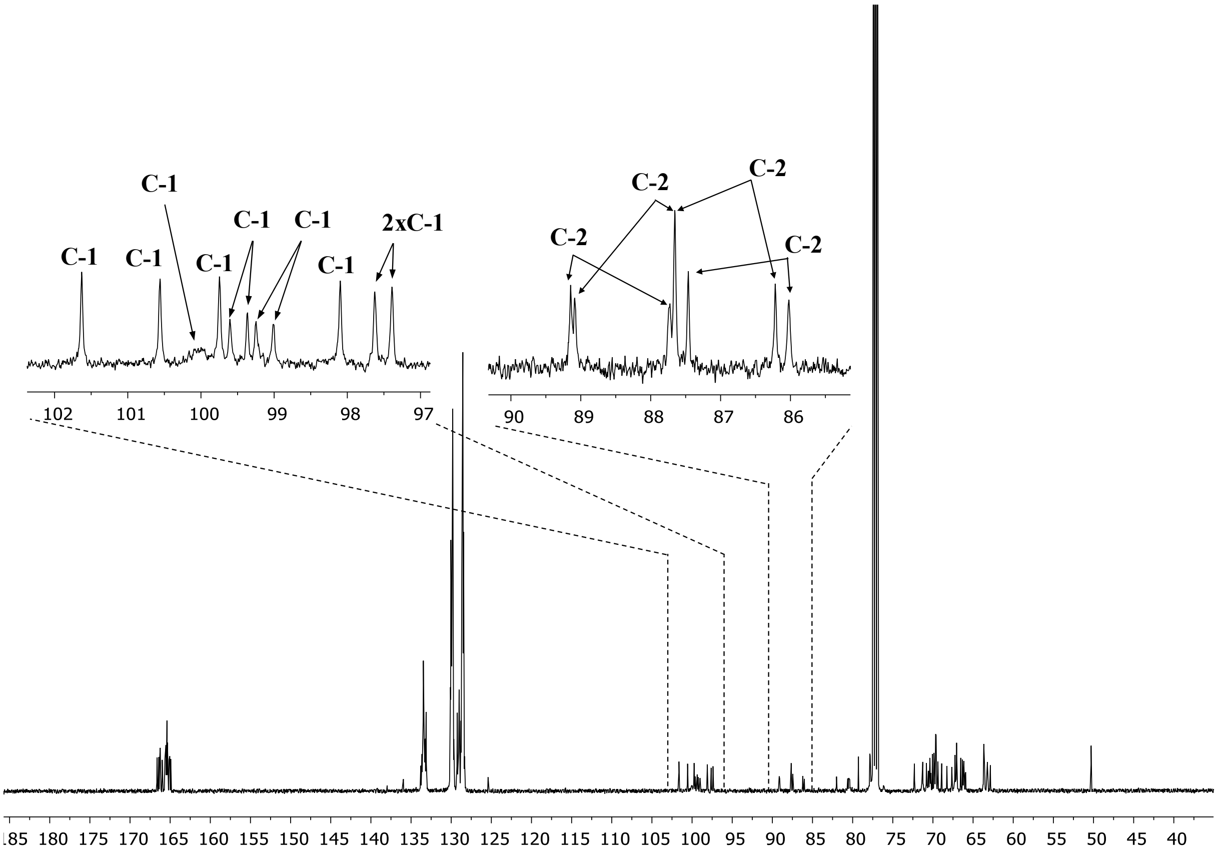

^{13}C-NMR spectrum of **21** (126 MHz, $CDCl_3$)

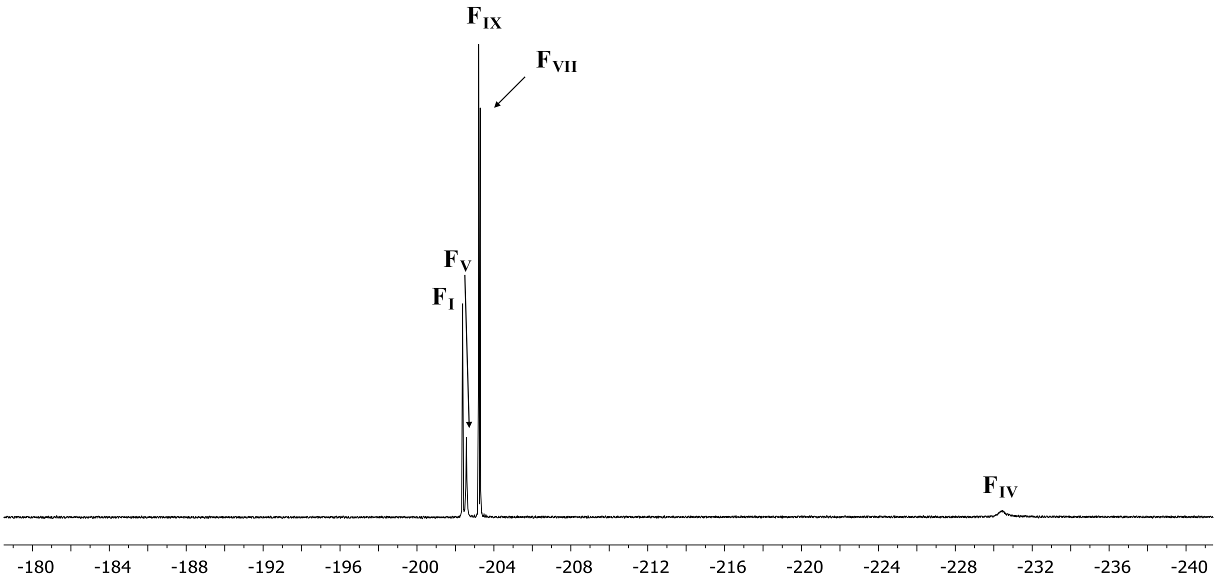

^{19}F-NMR spectrum of **21** (471 MHz, $CDCl_3$)

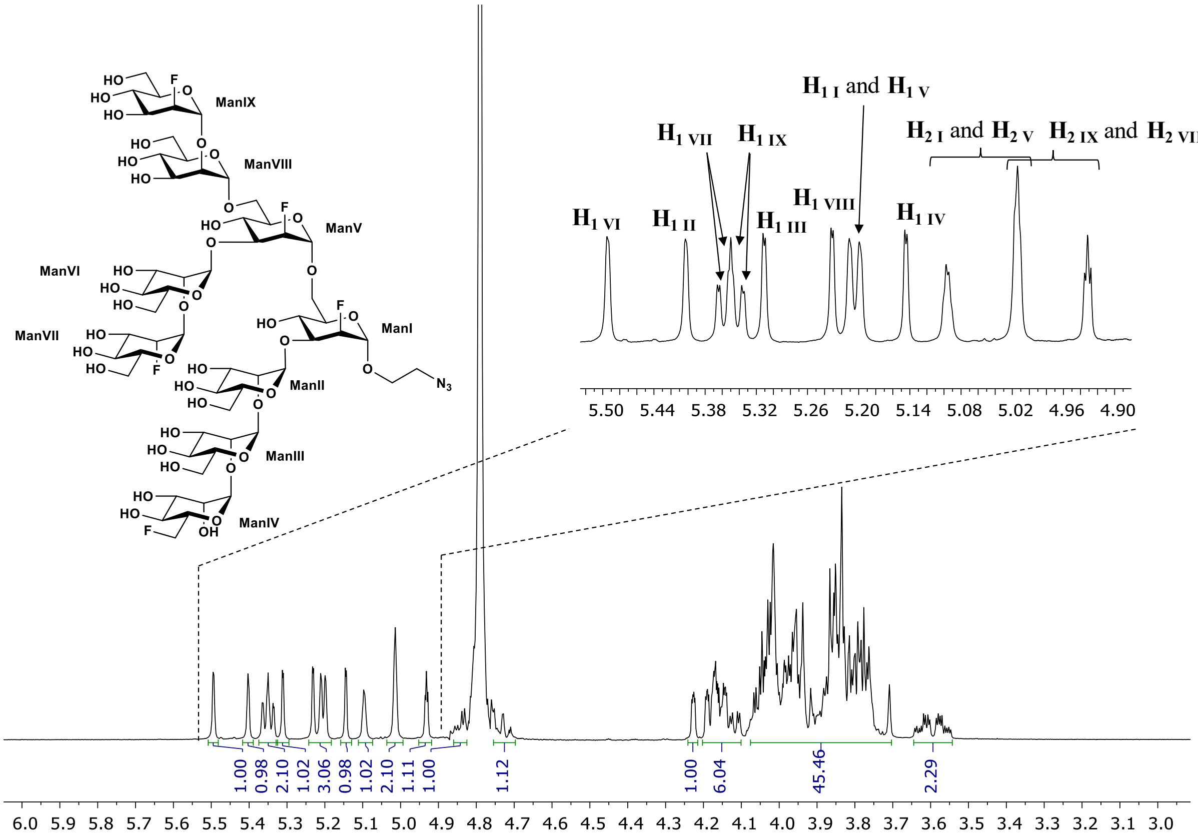

^{1}H-NMR spectrum of **1** (600 MHz, D_2O)

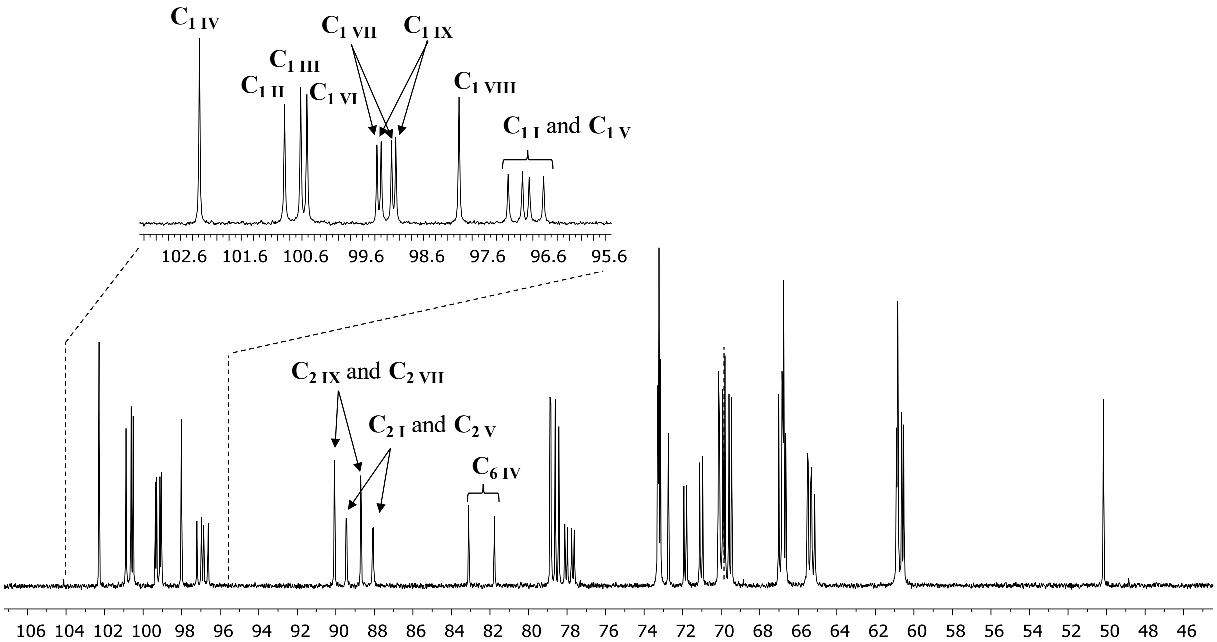

^{13}C-NMR spectrum of **1** (126 MHz, D_2O)

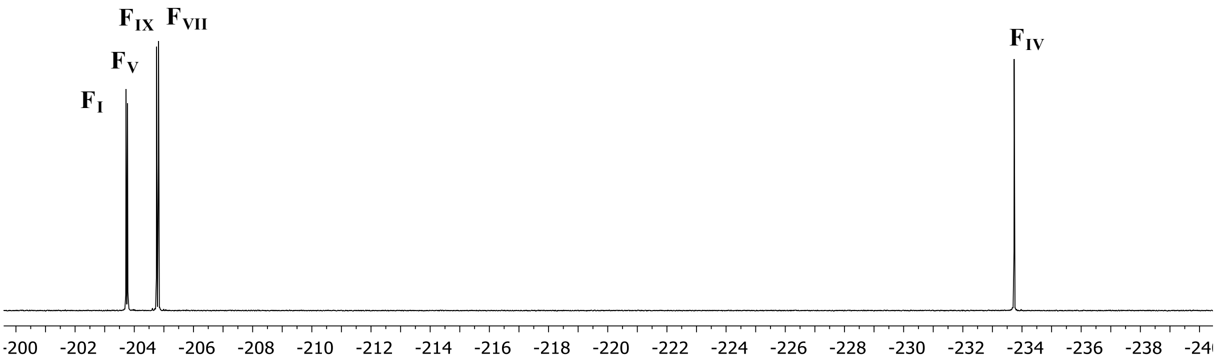

^{19}F-NMR spectrum of **1** (565 MHz, D_2O)

4. DC-SIGN sample preparation.

DC-SIGN Extracellular domain used for the NMR experiment is corresponding to residue 66 to 404. It has been produced in *E. coli* as inclusion bodies, refolded and purified as previously described in Tabarani *et al.*[4] Functionality, tetrameric oligomeric status and homogeneity of DC-SIGN ECD have been controlled through the purification procedure involving mannose affinity and size exclusion chromatography steps.

5. NMR experiments with DC-SIGN

NMR spectroscopy experiments were performed on a 600 MHz on a Bruker Avance III spectrometer equipped with a cryoprobe QCI Cryo 5mm (1H/19F 15N/13C) for ^{1}H, ^{15}N, ^{13}C, and ^{19}F with ^{2}H decoupling. NMR sample was prepared in 500 µL in buffer D_2O (150 mM NaCl, 4 mM $CaCl_2$, 25 mM *d*-Tris, pD 8), and with 50 µM DC-SIGN. The concentration of the ligand was 2 mM.

All experiments were carried out at 30 °C using the same sample. NOESY and tr-NOESY experiments were performed with mixing time 300 and 600 ms, respectively, with 48 scans. For 1D STD NMR a train of Gaussian shaped pulses of 50 ms and 25 ms spin-lock pulse prior to acquisition were used.[5] All the spectra were acquired with a spectral width of 12 kHz and 32K data points. Saturation times of 0.25, 0.5, 0.75, 1, 1.5, 2, 3, 4 and 5 s were used to obtain the STD build-up curves. Number of scans was 128 for 0.25, 0.5 and 0.75 s, 64 scans for 1 and 1.5 s, 32 scans for 2, 3, 4 and 5 s. The on-resonance frequency was set to 0.3 ppm and the off-resonance frequency was 40 ppm. For 2D STD-TOCSYreF 90º PC9 pulses of 15.7 ms length were used and the TOCSY mixing time was 60 ms. All the spectra were acquired with a spectral width of 28.4 kHz in the ^{19}F dimension using 8 K data points and 1.8 kHz in the ^{1}H dimension using 128 data points. Saturation times of 0.5, 1, 2 and 3 s were used to obtain the STD build-up curves. Number of scans was 32 for 0.5 and 1 s, 16 scans for 2 and 3 s. The on-resonance frequency was set to 0.3 ppm, and the off-resonance frequency was -30 ppm.

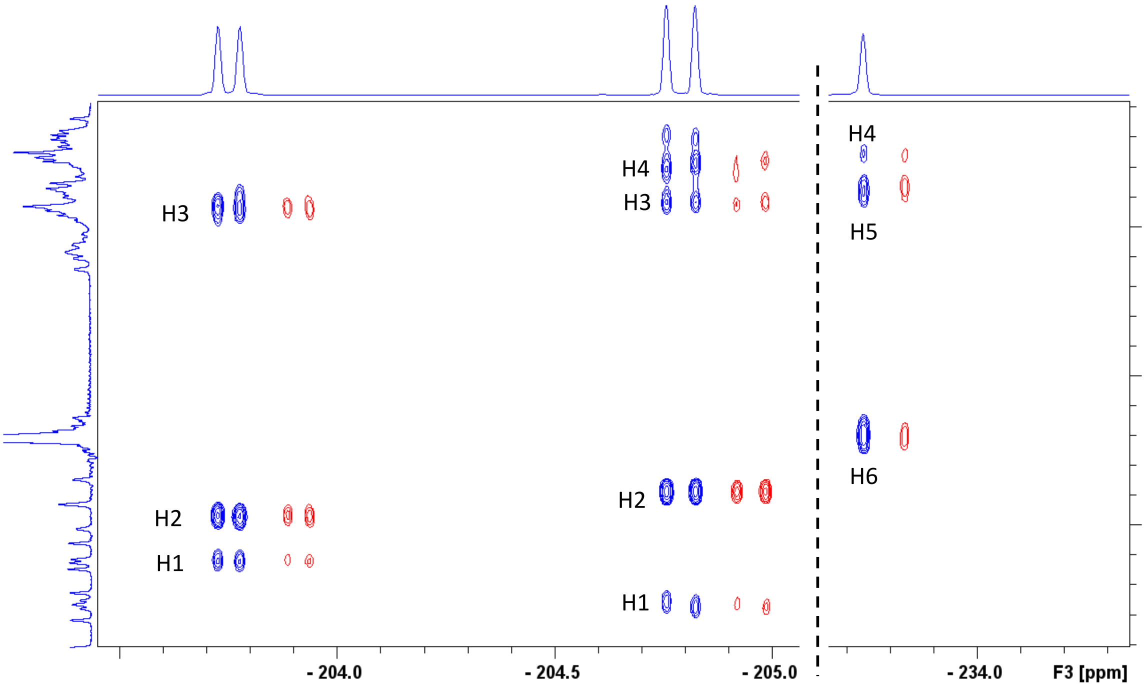

Figure S1. 2D-TOCSYreF off-resonance spectrum (blue) and 2D STD-TOCSYreF peaks at 3 s saturation time (red). ^{19}F NMR frequencies are given in the abscissa axis whereas ^{1}H NMR frequencies are in the ordinate axis. STD cross peaks are shifted to high field in the ^{19}F dimension to facilitate visual inspection.

To get the binding epitope from the STD NMR data and to minimize any T_1 relaxation bias, the STD build-up curves were fitted to the equation $STD(t_{sat}) = STD_{max}(1 - e^{(-ksat \cdot tsat)})$, calculating the initial growth rate STD_0 factor as $STD_{max} \cdot k_{sat}$ and then, the epitope was obtained by normalization of the whole set of initial slopes against the highest value, and expressing the result in percentage.[6-7]

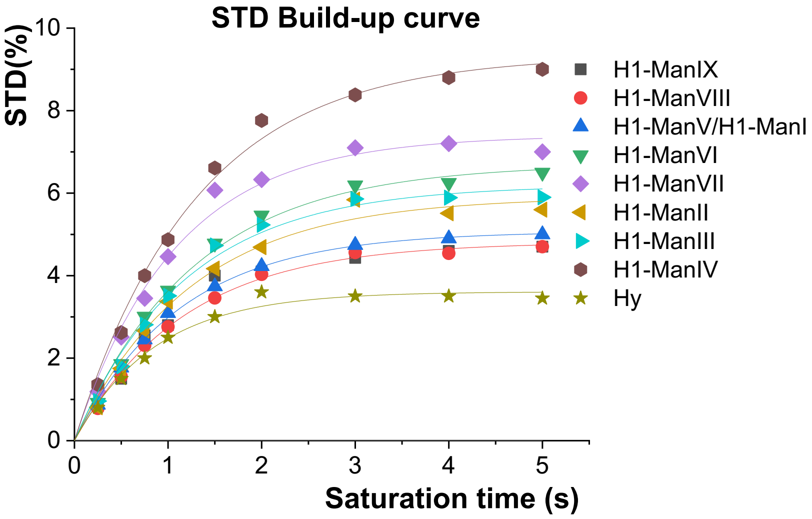

Figure S2. Binding of fluorinated Man$_9$ oligosaccharide to DC-SIGN studied by 1D ^{1}H STD NMR. STD NMR build-up curves. Temperature 30°C; saturation frequency 0.3 ppm.

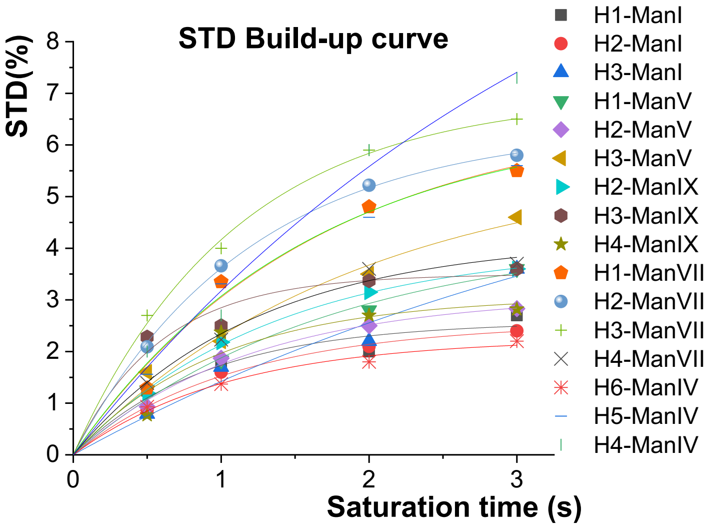

Figure S3. Binding of fluorinated Man₉ oligosaccharide to DC-SIGN studied by 2D ^{1}H,^{19}F STD-TOCSYreF NMR. Build-up curves. Temperature 30°C; saturation frequency 0.3 ppm.

Man_9-Arm	Proton - Residue	STD_0 (s^{-1})	$STD_{res\text{-}average}$ (s^{-1})	$STD_{relative}$ (%)
D3	H2-ManIX	3,02	4,01	84
	H3-ManIX	5,88		
	H4-ManIX	3,14		
Central	H1-ManV	2,47	2,67	56
	H2-ManV	2,59		
	H3-ManV	2,95		
D2	H1-ManVII	4,08	4,80	100
	H2-ManVII	5,34		
	H3-ManVII	6,35		
	H4-ManVII	3,44		
Reducing Ring	H1-ManI	2,87	2,26	47
	H2-ManI	2,32		
	H3-ManI	1,59		
D1	H6-ManIV	2,14	3,30	69
	H5-ManIV	4,14		
	H4-ManIV	3,63		

Table S1. Initial slope values (STD_0) obtained from the 2D STD-TOCSYreF NMR experiment and calculated residue-averaged relative STD values

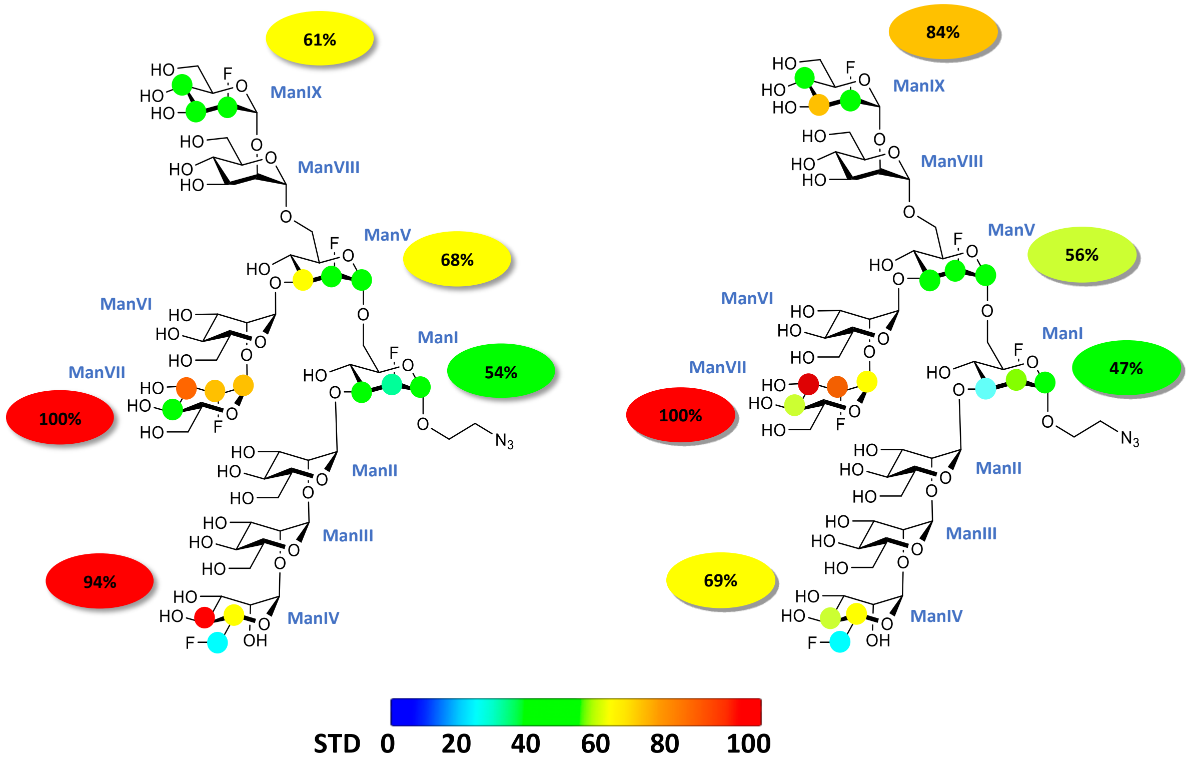

Figure S4. Comparison of the binding epitope mapping for the interaction of Man₉ oligosaccharide with DC-SIGN obtained by (Left) an analysis of a 2D ^{1}H,^{19}F STD-TOCSYreF NMR experiment at a single saturation time (3 s), and (Right) an analysis of initial slopes of 2D ^{1}H,^{19}F STD-TOCSYreF build-up curves. Temperature 30°C; saturation frequency 0.3 ppm. At one single 3 s saturation time analysis, there is an evident overestimation of the contribution to binding of residues ManIV, ManV, and ManIX, which can be ascribed to slower relaxation of protons on those residues.

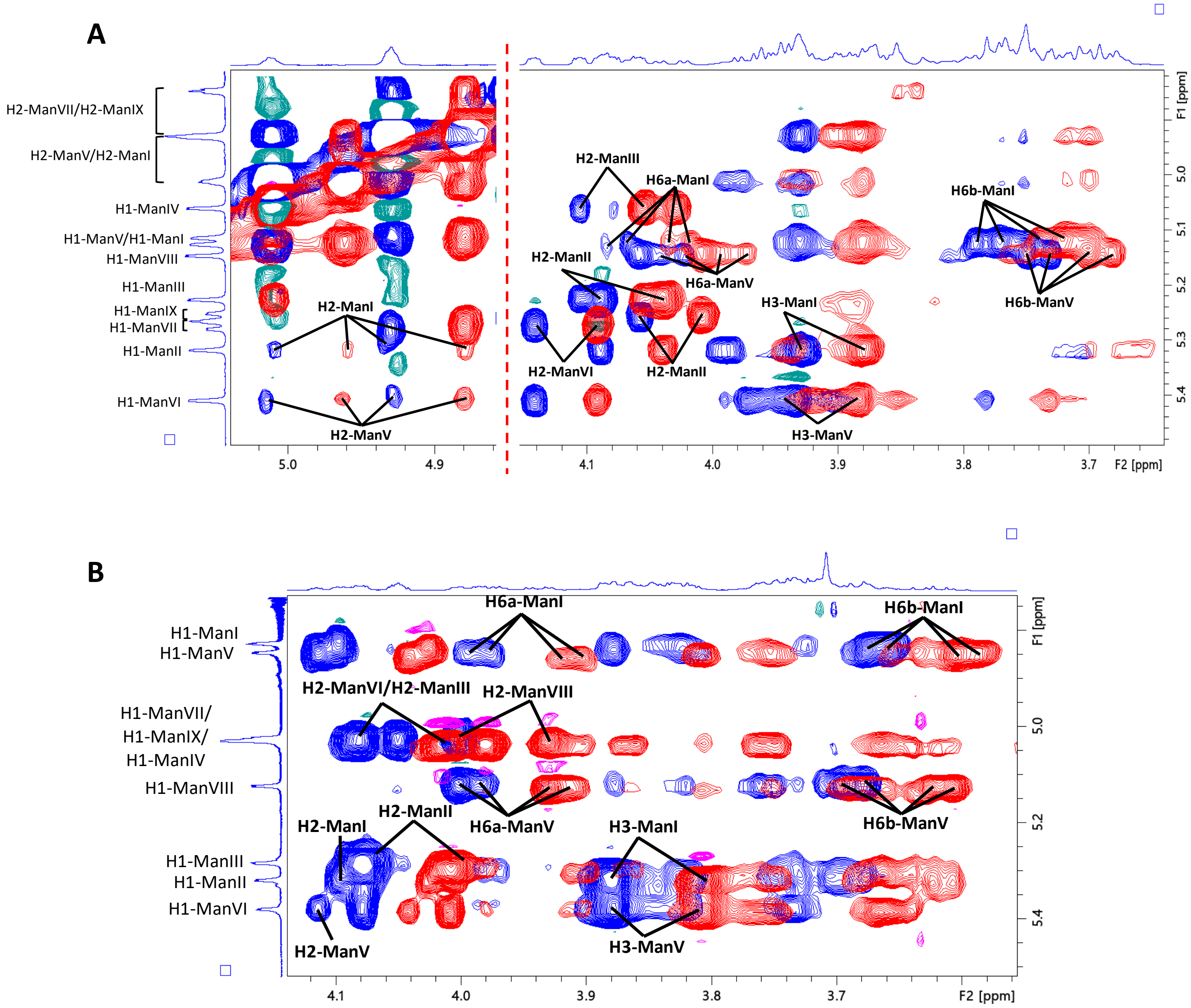

Figure S5. A) 2D NOESY and tr-NOESY spectra of F-Man$_9$. In red: tr-NOESY experiment (protein-ligand sample). In blue: NOESY experiment (ligand sample). B) 2D NOESY and tr-NOESY spectra of the non-fluorinated Man$_9$. In red: tr-NOESY experiment. In blue: NOESY experiment. In both cases, the red spectrum (protein-ligand sample) has been shifted to the right so that the signals can be clearly identified.

A

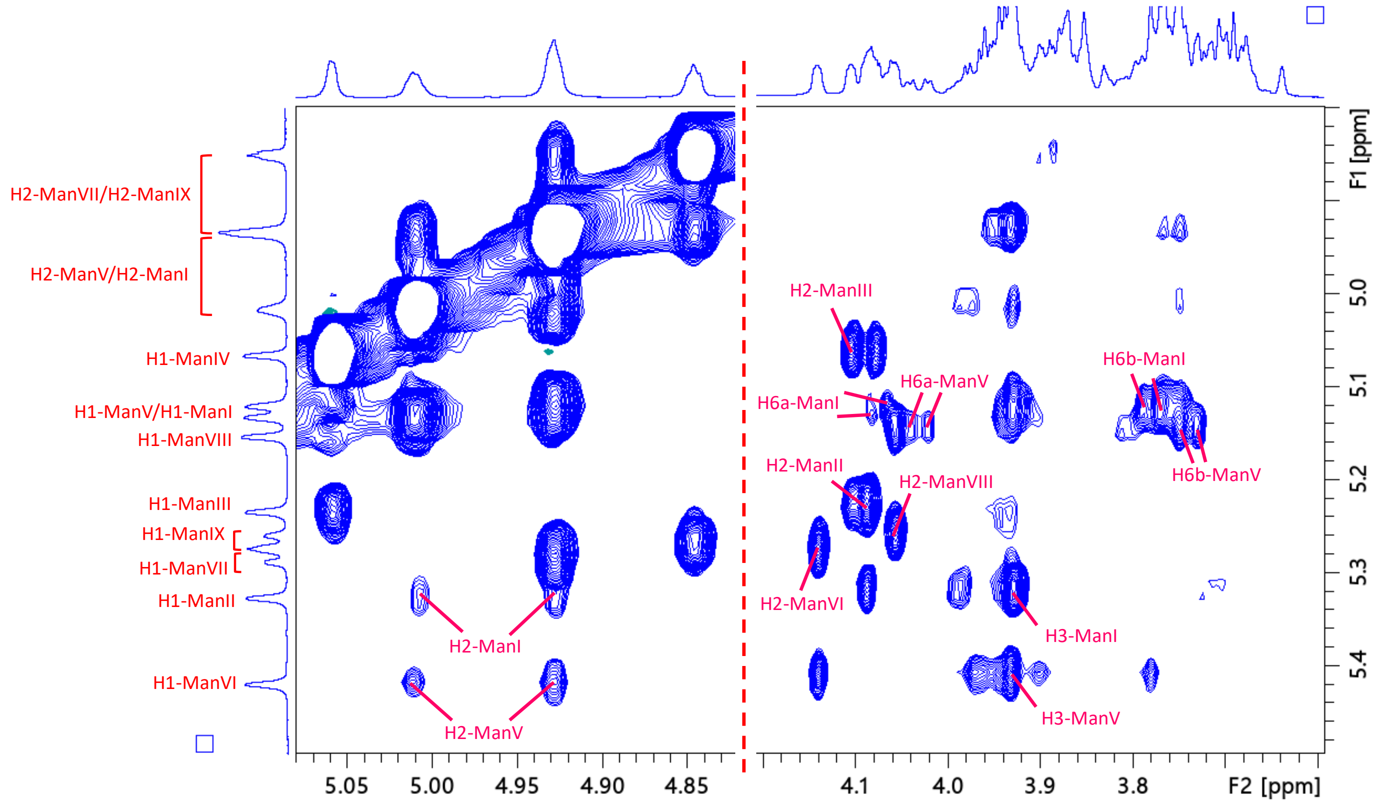
H2-ManVII/H2-ManIX
H2-ManV/H2-ManI
H1-ManIV
H1-ManV/H1-ManI
H1-ManVIII
H1-ManIII
H1-ManIX
H1-ManVII
H1-ManII
H1-ManVI
H2-ManI
H2-ManV
H2-ManIII
H6a-ManI
H6a-ManV
H6b-ManI
H6b-ManV
H2-ManII
H2-ManVIII
H2-ManVI
H3-ManI
H3-ManV
5.05
5.00
4.95
4.90
4.85
4.1
4.0
3.9
3.8
F2 [ppm]
F1 [ppm]
5.0
5.1
5.2
5.3
5.4

B

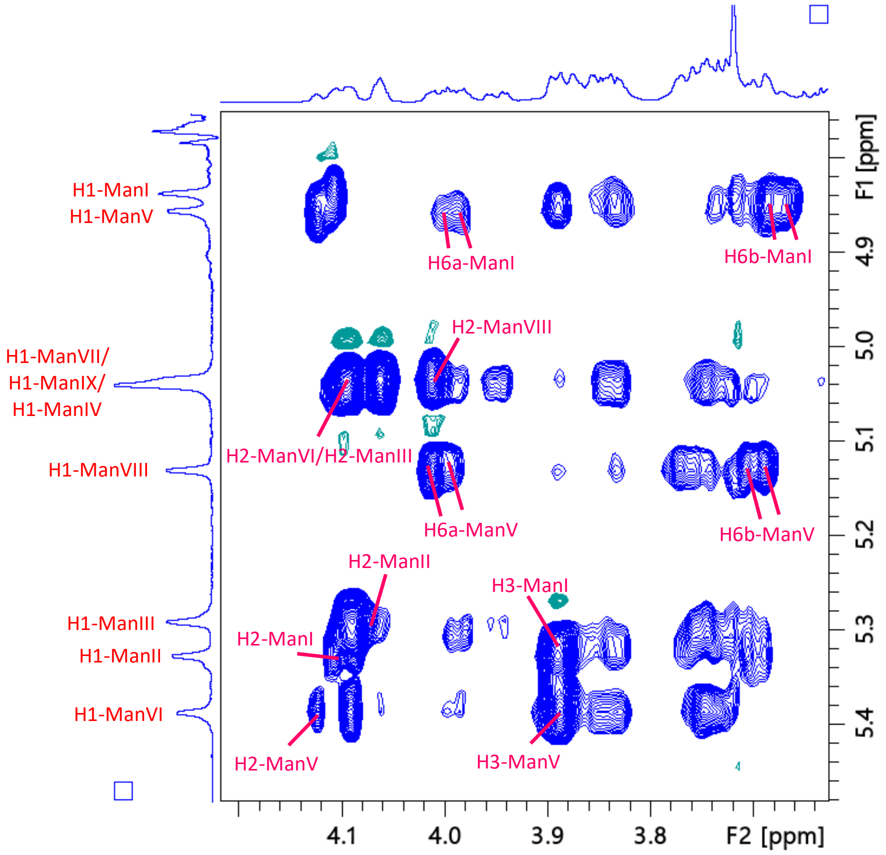
H1-ManI
H1-ManV
H6a-ManI
H6b-ManI
H1-ManVII/
H1-ManIX/
H1-ManIV
H2-ManVIII
H1-ManVIII
H2-ManVI/H2-ManIII
H6a-ManV
H6b-ManV
H2-ManII
H3-ManI
H1-ManIII
H1-ManII
H2-ManI
H1-ManVI
H2-ManV
H3-ManV
4.1
4.0
3.9
3.8
F2 [ppm]
F1 [ppm]
4.9
5.0
5.1
5.2
5.3
5.4

C

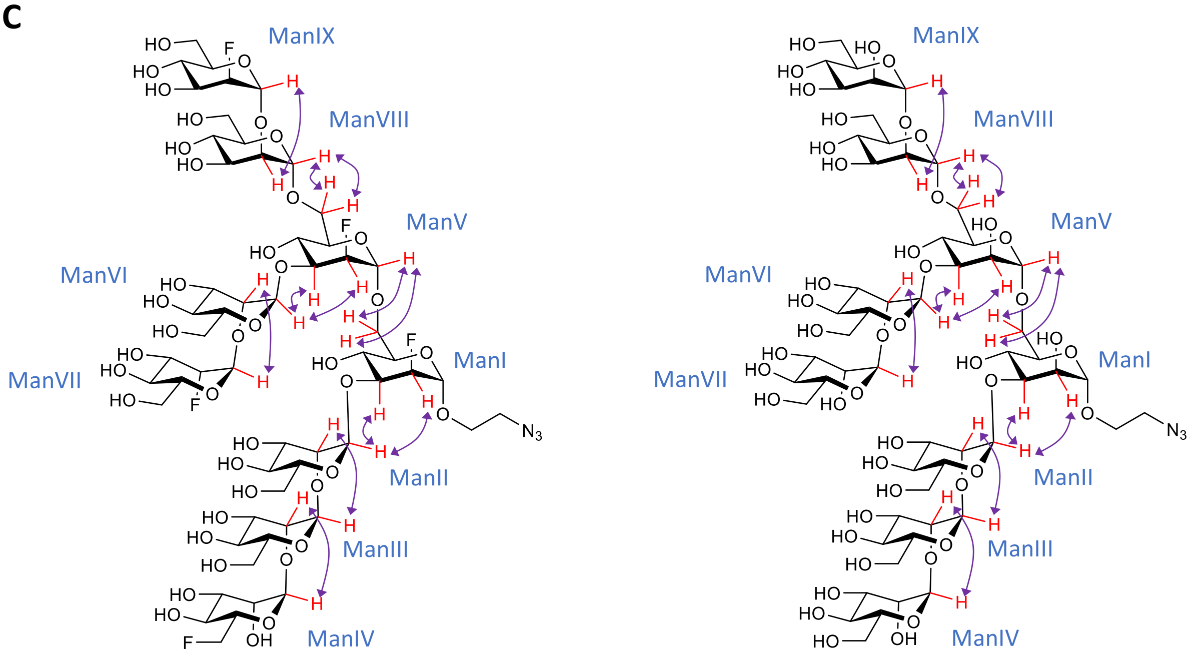

Figure S6. A) 2D tr-NOESY spectrum of F-Man₉. Conformationally key strongest NOEs are indicated. B) 2D tr-NOESY spectrum of the non-fluorinated Man₉. Conformationally key strongest NOEs are indicated. C) Structures of fluorinated and non-fluorinated Man₉ oligosaccharides indicating the observed conformationally key strongest NOEs (purple arrows). The similarity in the patterns of observed interglycosidic NOEs strongly supports similar conformational behaviour of both oligosaccharides.

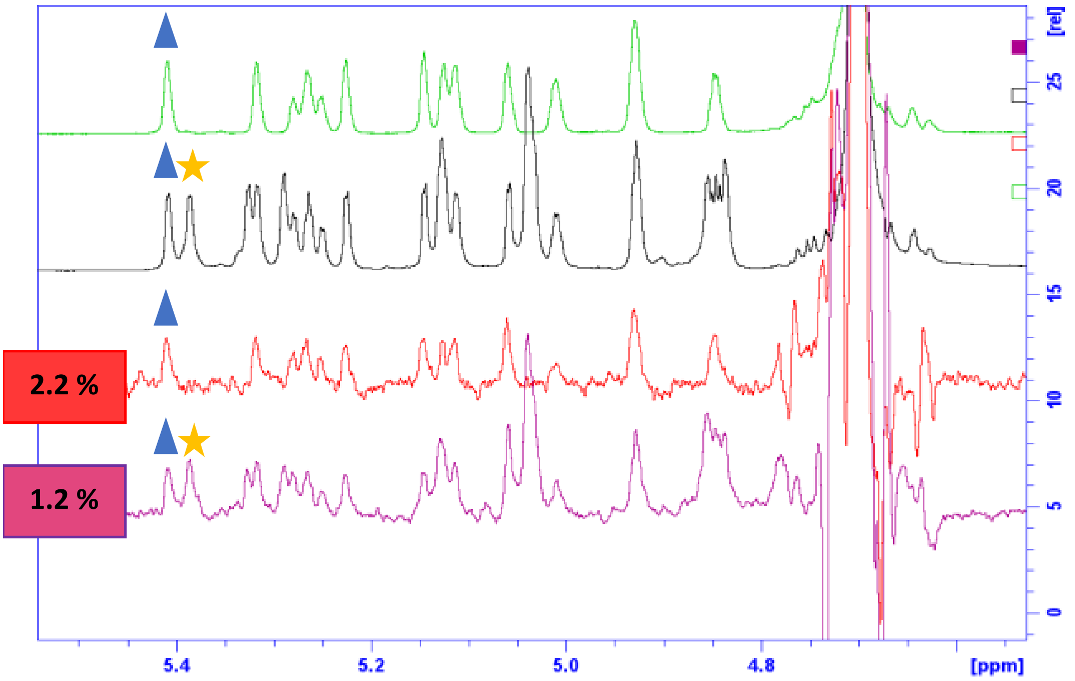

Figure S7. STD NMR competition study between fluorinated and natural Man_9 for DC-SIGN. The green spectrum corresponds to the ^{1}H NMR spectrum of F-Man_9 with DC-SIGN. The black spectrum corresponds to the ^{1}H NMR spectrum of the same F-Man_9 sample with addition of an equimolar concentration of the natural Man_9 in the presence of DC-SIGN. The red spectrum corresponds to the STD NMR spectrum of F-Man_9 with DC-SIGN. The purple spectrum corresponds to the STD NMR spectrum of the F-Man_9 sample with an equimolar concentration Man_9. The blue triangle represents the monitored isolated anomeric proton of F-Man_9. The yellow star represents an anomeric proton of natural Man_9. Upon addition of Man_9 the STD response of F-Man_9 decreased about 50%, supporting same affinity for both F-Man_9 and Man_9 ligands for DC-SIGN.

6. Molecular dynamics

A total of six MD simulations were run, three for each the natural and fluorinated Man_9 ligands. The three simulations of each ligand correspond to three different starting structures where the residue adjacent to the non-reducing terminal of each of the nonamannoside branches (D1, D2 and D3, corresponding to ManIII, ManVI, and ManVIII, respectively) coordinate the Ca^{2+} ion of DC-SIGN CRD.

Input preparation and equilibration

The initial coordinates of free Man_9 were built from the coordinates of $Man_9GlcNAc_2$ obtained by NMR analysis as reported by Woods *et al*,[8] followed by (i) manual removal of both GlcNAc residues, and (ii) substitution of the terminal hydroxyl group at the reducing end mannose residue by a O-CH_2-CH_2-N_3 group (in axial configuration), using *Pymol*. Then, the free ^{19}F-Man_9 model was generated from the Man_9 structure by manually substituting the specific hydroxyl groups by fluorine atoms with *Pymol*.[9]

The six starting protein-ligand 3D models for the D1, D2 and D3 Ca^{2+}-coordinated orientations were built by superimposition of the non-reducing and adjacent mannose residues of each branch to the alike residues of the Man_6 ligand bound to DC-SIGN CRD in the PDB structure 2IT5.[10] It should be noted that this PDB structure shows two possible orientations for Man_6, which are the so-called major and minor orientations. For ligand superimposition, we resorted to the experimental ^{1}H STD NMR data (see Figure 1 of the main text), which clearly indicated that for the nonamannoside only a bound conformation with a "minor" orientation of the non-reducing dimannoside on each arm is in agreement with

the experimental binding epitope. The STD NMR data indicated that the anomeric protons of both the non-reducing and adjacent residues point towards the protein surface, whereas they would be solvent exposed in the "major" orientation. No significant steric clashes were observed between the oligosaccharides and the protein in any of the orientations.

The MD simulation setup and equilibration were performed with the BioExcel Building Blocks (*BioBB*) library.[11] The ligands were parametrized and minimized using the *acpype* and *babel* modules, respectively, of BioBB (*biobb_chemistry.acpype* and *biobb_chemistry.babel*). The minimization of the ligands was performed with the steepest descent method and the GAFF force field. The topology of the complexes were generated with the *biobb_amber.leap* module, using the ff14SB force field[12] for the protein and GAFF[13] for the ligand. Subsequently, they were minimized with the *biobb_amber.sander* module using first positional restraints of 50 kcal/mol·$Å^2$ on the protein heavy atoms and, secondly, positional restraints of 500 kcal/mol·$Å^2$ on the ligand to avoid potential changes in ligand orientation due to protein repulsion. Then, each protein-ligand complex was immersed in a TIP3P[14] truncated octahedron water box with a distance from the protein to the box edge of 9.0 Å and Periodic Boundary Conditions, followed by the addition of a 150 mM concentration of NaCl. This gave rise to MD simulation systems of ~ 27,000 atoms. Each solvated system was minimized using the steepest descent protocol and applying positional restraints of 15 kcal/mol·$Å^2$ to the ligand, followed by heating up to 300 K over 2500 steps applying the Langevin thermostat[15] with a collision frequency of 1 ps^{-1} and positional restraints on the ligand of 10 kcal/mol·$Å^2$ (the *biobb_amber.sander* module was used). Next, each system was subjected to NVT followed by NPT equilibration of 100 ps each. A non-bonded interactions cutoff of 10.0 Å, the SHAKE algorithm for constraining the length of bonds involving hydrogen atoms, the Langevin thermostat[15] with a collision frequency of 5 ps^{-1}, and smooth positional restraints on the ligand (5 and 2.5 kcal/mol·$Å^2$ for NVT and NPT, respectively) were employed. During the NPT equilibration, a pressure of 1 bar was kept constant using isotropic position scaling with a pressure relaxation time of 2 ps.

MD production run

A 500-ns of MD production run was carried out for each complex on a AMD-Ryzen 4xGPU 3070 Computing Cluster using the *pmemd.cuda* module of AMBER 20.[16] The production dynamics was performed at a constant temperature of 300 K, by applying the Langevin thermostat[15] with a collision frequency of 1 ps^{-1}, and a constant pressure of 1 bar (using isotropic position scaling with a pressure relaxation time of 1 ps). A non-bonded interactions cutoff of 9.0 Å, periodic boundary conditions (PBC),[17] and the Particle Mesh Ewald method[18] (PME) to account for the long range electrostatic effect were employed. The SHAKE algorithm[19-20] was also employed, thus allowing 2 fs between time steps. Trajectory coordinates were saved every 100 picoseconds.

MD analysis: protein-branch contacts

Firstly, the six MD trajectories were subjected to visual inspection to check that the starting Ca^{2+} coordination was maintained throughout the simulation. The analysis of the trajectories was performed using the *cpptraj* module (version *4.25.6*) of AMBER 20.[16] The first 50 ns of MD trajectory was disregarded for the analysis since the protein backbone was still equilibrating during the first nanoseconds of production run. To carry out the analysis of the total number contacts established by each high mannose branch to the protein we used the *nativecontacts* command, using a distance cutoff of 4 Å. Only protons at the non-reducing mannose residue of each branch were considered in the analysis since these are the residues that we could characterized by STD TOCSYreF experiments (see Figure 2 of the main text). From the protein side, only protein protons accounting for the experimental STD NMR conditions, which relies on interproton protein-ligand transfer of magnetization, were considered in the analysis.

Following this contacts analysis, the MD-derived "epitopes" shown in Figure 3 were obtained by addition of the total number of contacts established by each branch with the protein in each of the three Ca^{2+}-coordinating models, and subsequently normalized against the highest one.

7. References

1. Axer, A.; Jumde, R. P.; Adam, S.; Faust, A.; Schafers, M.; Fobker, M.; Koehnke, J.; Hirsch, A. K. H.; Gilmour, R., Enhancing glycan stability via site-selective fluorination: modulating substrate orientation by molecular design. *Chem Sci* **2020,** *12* (4), 1286-1294.
2. Reina, J. J.; Di Maio, A.; Ramos-Soriano, J.; Figueiredo, R. C.; Rojo, J., Rapid and efficient synthesis of α(1–2)mannobiosides. *Org. Biomol. Chem.* **2016,** *14* (10), 2873-2882.
3. Zihlmann, P.; Silbermann, M.; Sharpe, T.; Jiang, X.; Mühlethaler, T.; Jakob, R. P.; Rabbani, S.; Sager, C. P.; Frei, P.; Pang, L.; Maier, T.; Ernst, B., KinITC—One Method Supports both Thermodynamic and Kinetic SARs as Exemplified on FimH Antagonists. *Chemistry – A European Journal* **2018,** *24* (49), 13049-13057.
4. Tabarani, G.; Thépaut, M.; Stroebel, D.; Ebel, C.; Vivès, C.; Vachette, P.; Durand, D.; Fieschi, F., DC-SIGN neck domain is a pH-sensor controlling oligomerization: SAXS and hydrodynamic studies of extracellular domain. *The Journal of biological chemistry* **2009,** *284* (32), 21229-21240.
5. Mayer, M.; Meyer, B., Characterization of Ligand Binding by Saturation Transfer Difference NMR Spectroscopy. *Angew Chem Int Ed Engl* **1999,** *38* (12), 1784-1788.
6. Mayer, M.; Meyer, B., Group epitope mapping by saturation transfer difference NMR to identify segments of a ligand in direct contact with a protein receptor. *J Am Chem Soc* **2001,** *123* (25), 6108-17.
7. Mayer, M.; James, T. L., NMR-Based Characterization of Phenothiazines as a RNA Binding Scaffold. *Journal of the American Chemical Society* **2004,** *126* (13), 4453-4460.
8. Woods, R. J.; Pathiaseril, A.; Wormald, M. R.; Edge, C. J.; Dwek, R. A., The high degree of internal flexibility observed for an oligomannose oligosaccharide does not alter the overall topology of the molecule. *Eur J Biochem* **1998,** *258* (2), 372-86.
9. Schrödinger, LLC. The {PyMOL} Molecular Graphics System, Version 1.8; 2015.
10. Feinberg, H.; Castelli, R.; Drickamer, K.; Seeberger, P. H.; Weis, W. I., Multiple Modes of Binding Enhance the Affinity of DC-SIGN for High Mannose *N*-Linked Glycans Found on Viral Glycoproteins. *J. Biol. Chem.* **2007,** *282* (6), 4202-4209.
11. Andrio, P.; Hospital, A.; Conejero, J.; Jorda, L.; Del Pino, M.; Codo, L.; Soiland-Reyes, S.; Goble, C.; Lezzi, D.; Badia, R. M.; Orozco, M.; Gelpi, J. L., BioExcel Building Blocks, a software library for interoperable biomolecular simulation workflows. *Sci Data* **2019,** *6* (1), 169.
12. Maier, J. A.; Martinez, C.; Kasavajhala, K.; Wickstrom, L.; Hauser, K. E.; Simmerling, C., ff14SB: Improving the Accuracy of Protein Side Chain and Backbone Parameters from ff99SB. *J Chem Theory Comput* **2015,** *11* (8), 3696-713.

13. Wang, J.; Wolf, R. M.; Caldwell, J. W.; Kollman, P. A.; Case, D. A., Development and testing of a general amber force field. *J Comput Chem* **2004,** *25* (9), 1157-74.
14. Jorgensen, W. L.; Chandrasekhar, J.; Madura, J. D.; Impey, R. W.; Klein, M. L., Comparison of simple potential functions for simulating liquid water. *The Journal of Chemical Physics* **1983,** *79* (2), 926-935.
15. Izaguirre, J. A.; Catarello, D. P.; Wozniak, J. M.; Skeel, R. D., Langevin stabilization of molecular dynamics. *The Journal of Chemical Physics* **2001,** *114* (5), 2090-2098.
16. D.A. Case, K. B., I.Y. Ben-Shalom, S.R. Brozell, D.S. Cerutti, T.E. Cheatham, III, V.W.D. Cruzeiro, T.A. Darden, R.E. Duke, G. Giambasu, M.K. Gilson, H. Gohlke, A.W. Goetz,R Harris, S. Izadi, S.A. Izmailov, K. Kasavajhala, A. Kovalenko, R. Krasny, T. Kurtzman, T.S. Lee, S. LeGrand, P. Li, C. Lin, J. Liu, T. Luchko, R. Luo, V. Man, K.M. Merz, Y. Miao, O. Mikhailovskii, G. Monard, H. Nguyen, A. Onufriev, F. Pan, S. Pantano, R. Qi, D.R. Roe, A. Roitberg, C. Sagui, S. Schott-Verdugo, J. Shen, C.L. Simmerling, N.R. Skrynnikov, J. Smith, J. Swails, R.C. Walker, J. Wang, L. Wilson, R.M. Wolf, X. Wu, Y. Xiong, Y. Xue, D.M. York and P.A. Kollman, AMBER 2020. **2020, University of California, San Francisco**.
17. York, D. M.; Darden, T. A.; Pedersen, L. G., The effect of long-range electrostatic interactions in simulations of macromolecular crystals: A comparison of the Ewald and truncated list methods. *The Journal of Chemical Physics* **1993,** *99* (10), 8345-8348.
18. Petersen, H. G., Accuracy and efficiency of the particle mesh Ewald method. *The Journal of Chemical Physics* **1995,** *103* (9), 3668-3679.
19. Ryckaert, J.-P.; Ciccotti, G.; Berendsen, H. J. C., Numerical integration of the cartesian equations of motion of a system with constraints: molecular dynamics of n-alkanes. *Journal of Computational Physics* **1977,** *23* (3), 327-341.
20. Miyamoto, S.; Kollman, P. A., Settle: An analytical version of the SHAKE and RATTLE algorithm for rigid water models. *Journal of Computational Chemistry* **1992,** *13* (8), 952-962.
